# Optimization and Stability of Cell–Polymer Hybrids Obtained by “Clicking” Synthetic Polymers to Metabolically Labeled Cell Surface Glycans

**DOI:** 10.1021/acs.biomac.9b00478

**Published:** 2019-06-15

**Authors:** Ruben M. F. Tomás, Matthew I. Gibson

**Affiliations:** † Department of Chemistry, University of Warwick, Coventry CV4 7AL, United Kingdom; ‡ Warwick Medical School, University of Warwick, Coventry CV4 7AL, United Kingdom

## Abstract

Re-engineering of mammalian cell surfaces with polymers enables the introduction of functionality including imaging agents, drug cargoes or antibodies for cell-based therapies, without resorting to genetic techniques. Glycan metabolic labeling has been reported as a tool for engineering cell surface glycans with synthetic polymers through the installation of biorthogonal handles, such as azides. Quantitative assessment of this approach and the robustness of the engineered coatings has yet to be explored. Here, we graft poly(hydroxyethyl acrylamide) onto azido-labeled cell surface glycans using strain-promoted azide–alkyne “click” cycloaddition and, using a combination of flow cytometry and confocal microscopy, evaluate the various parameters controlling the outcome of this “grafting to” process. In all cases, homogeneous cell coatings were formed with >95% of the treated cells being covalently modified, superior to nonspecific “grafting to” approaches. Controllable grafting densities could be achieved through modulation of polymer chain length and/or concentration, with longer polymers having lower densities. Cell surface bound polymers were retained for at least 72 h, persisting through several mitotic divisions during this period. Furthermore, we postulate that glycan/membrane recycling is slowed by the steric bulk of the polymers, demonstrating robustness and stability even during normal biological processes. This cytocompatible, versatile and simple approach shows potential for re-engineering of cell surfaces with new functionality for future use in cell tracking or cell-based therapies.

## Introduction

Cell-based therapies have expanded the repertoire of tools in modern medicine providing an arsenal of treatments in addition to conventional drugs or protein-based therapies. Chimeric antigen receptor (CAR)-T cell therapies have rapidly emerged as a potential treatment for multiple hematological malignancies through the introduction of cancer targeting receptors on T-cell surfaces.^1–3^ However, viral vector transduction of T-cells, the process which randomly inserts the CAR transgenes into the genome, presents risks of insertional oncogenesis and gene silencing. Manufacturing practicality is also a concern due to production and quality control costs along with safety and ethical concerns.^4,5^ Gene knock-in can mitigate some of these caveats; however, the efficiency of this procedure is 20%, compared to 68% for retroviruses, and off-target mutagenesis is still a concern.^6,7^ Thus, unmodified cells require significant purification and separation. Genetic techniques are also not easily adaptable to introduce nonbiotic components such as drugs, tracking modalities, and non-natural amino acids.^8^ Hence, there is considerable opportunity to explore synthetic tools to adapt cell surfaces both in medicine and bioscience.

Re-engineering of mammalian cell surfaces with synthetic polymers is emerging as an approach to enable rapid, simple and versatile chemical remodeling of cells to introduce non-natural functionality. Masking of cell surface antigens of erythrocytes and islet cells has been widely explored using polymer coatings to improve blood transfusions,^9–11^ reduce malaria parasite binding,^12^ and prevent the rejection of islet transplants.^13^ Enhancement of islet transplants has also been achieved by controlling the immediate blood mediated inflammatory reaction through the addition of natural polymers, including heparin,^14^ thrombomodulin^15^ and urokinase.^16^ Additionally, cell–cell interactions can be controlled through the addition of biotin/avidin interactions and hybridization of oligoDNA demonstrating potential future roles in inducing stem cell differentiation for cell-based therapies.^17^

Despite cell surface engineering with natural and synthetic polymers presenting many potential roles in cell-based therapies, challenges arise in the formation of effective, clinical-translatable methods. Amphiphilic polymers, such as alkylated poly(vinyl alcohol) or lipid-based poly(ethylene glycol),^18,19^ allow direct passive insertion into the lipid bilayer membranes with minimal impact to membrane integrity and structures. Glycocalyx remodeling using lipid-based glycoconjugates has been demonstrated to be a powerful tool to introduce specific glycan epitopes to the cell-surface, allowing mediation of multiple biological processes. Bertozzi et al. demonstrated that introducing lipid-based Siglec-7 allows immunomodulation of the innate immune system, preventing natural-killer mediated killing of allogeneic and xenogenic primary cells.^20^ Rat cortical neurons engineered with lipid-terminated chondroitin sulfate glycosaminoglycans (GAGs) have been used to enhance nerve growth factor-mediated signaling and promote neural outgrowth.^21^ Additionally, neural differentiation of embryonic stem cells has been achieved through membrane incorporation of neoproteoglycans.^22^ However, dissociation of lipid-based polymers occurs in under 24 h, with reports of a 50% drop within 8 h,^22^ due to intrinsic membrane turnover processes.^21,23,24^ Thus, biological applications are limited to this time frame and the nonspecific nature of lipid insertion methods.

Additional noncovalent approaches include electrostatic deposition of polycations onto the negatively charged membrane. These approaches dramatically and rapidly reduce cell viability (<1 h), severely damaging the cell membrane even when contact is minimized with the incorporation of polyethylene glycol (PEG) chains.^25–27^ Cell membrane proteins remain one of the most exploited sites for antibody conjugation in immunotherapy, especially tyrosine and selenocysteine residues.^28^ However, biocompatibility of protein conjugation approaches varies due to mammalian cell sensitivity to cell surface modification. Hawker et al. demonstrated that covalent conjugation of chain transfer agent (CTA) initiators for “grafting-from” approaches to membrane proteins resulted in extensive mechanical stress leading to cell death.^29^ Limitations of grafting-from approaches arise due to denaturing of proteins and side-reactions during the polymerization with protein side-chain functional groups. Furthermore, most protein conjugation approaches only last 24–48 h^18^ and will nonspecifically bind to any cell type. Existing site-specific amino acid modifications mainly rely on the use of cytotoxic heavy metal catalysts.^30–35^ A successful polymer conjugation approach should be simple, robust, biorthogonal and biocompatible.

Cell surface glycans are desirable binding sites for synthetic and natural polymer conjugation due to their high abundance and major structural, metabolic and recognition roles in biology.^36–38^ Bertozzi and co-workers have pioneered the use of metabolic glycan labeling to introduce non-natural functionality into cell-surface glycans.^39,40^ For example, peracetylated *N*-azidoacetylmannosamine (Ac_4_ManNAz) can be used to “hijack” the sialic acid biosynthetic pathway to incorporate azides on surface sialic acids. Metabolic oligosaccharide engineering has already proven potential usefulness in cancer immune therapies by offering biorthogonal handles for natural polymer conjugation. Shi et al. demonstrated that metabolically labeled human peripheral blood mononuclear cells modified with alkynyl-PEG-*β*-cyclodextrin and photoswitchable azobenzene-MUC1 aptamers could be used to target epithelial cancer cells (MCF-7) controllably, forming a T-cells-cancer cell assembly.^41^ Furthermore, Wang et al. have demonstrated that covalent conjugation of silica nanoconjugates to metabolically labeled cell surface glycans can promote uptake for potential drug delivery systems.^42^ Selective labeling of cells with azido sugars has also been achieved via liposomal delivery targeting overexpressed surface receptors and *in vivo* using “caged” azido sugars which label cells possessing cancer-overexpressed enzymes.^43,44^ Tomás and Gibson have demonstrated that telechelic polymers generated by reversible activation fragmentation transfer (RAFT) polymerization can be used to install polymers rapidly and simply onto metabolically labeled cells, exclusively at the cell surface.^45^ Cell surface grafting using this approach demonstrated several advantages to conventional methods including cytocompatibility, biorthogonality, and selectivity. However, optimization and quantitative assessment of the efficiency, robustness, and stability of metabolic oligosaccharide engineering for synthetic polymer conjugation remains to be explored.

Here, we present a detailed flow cytometry-based study on the optimization and ability to label and graft synthetic polymers to living cells using azido handles installed onto cell surface glycans. This study reveals a high degree of selective cell labeling versus nonspecific conjugation methods, with more than 95% of cells being covalently labeled. Polymers persisted on the cell surface through multiple cell division processes demonstrating robustness without negatively impacting cell function. These results provide a guide to simple, robust and biorthogonal re-engineering of cell surfaces to explore their biomedical and biotechnological impact.

## Experimental Section

### Materials

Ammonium fluoroborate (NH_4_BF_4_), dodecanethiol, tripotassium phosphate (K_3_PO_4_), 2-bromo-2-methylpropionic acid, dichloromethane (DCM), hydrochloric acid (HCl), magnesium sulfate (MgSO_4_), hexane, 4-(dimethylamino) pyridine, *N*-(3-(dimethylamino)propyl)-*N*′-ethylcarbodiimide hydrochloride, penta-fluorophenol (PFP), sodium bicarbonate (NaHCO_3_), sodium chloride (NaCl), *N*-hydroxyethyl acrylamide (HEA), 4,4′-azobis(4-cyanovaleric acid) (ACVA), toluene, methanol, mesitylene, dibenzocyclooctyne-amine (DBCO-NH_2_), propyl amine, paraformaldehyde, phosphate-buffered saline preformulated tablets, dimethylformamide, *N*-(5-fluoresceinyl)maleimide, calcium chloride (CaCl), and magnesium chloride (MgCl) were purchased from Sigma-Aldrich Co Ltd. (Gillingham, UK) and used without further purification. Dulbecco phosphate buffered saline (DPBS), *N*-azidoacetylmannosamine-tetraacylated (Ac_4_ManNAz), and BD FACSFlow Sheath Fluid were purchased from Fisher Scientific (Loughborough, UK). *α*2-3,6,8,9 Neuraminidase A (316 000 units·mg^−1^) was purchased from New England BioLabs (UK) LTD (Hitchin).

### Physical and Analytical Methods

#### Nuclear Magnetic Resonance (NMR) Spectroscopy

NMR spectra of PFP-DMP (in CDCl_3_), PFP-pHEA_*n*_, and DBCO-pHEA_*n*_ (in MeOD) were recorded on a Bruker HD-300 spectrometer at 298 K. Chemical shifts were reported as *δ* in parts per million (ppm) relative to residual nondeuterated solvent resonances (CDCl_3_^1^H: *δ* = 7.26 ppm; ^13^C *δ* = 77.16. CD_3_OD ^1^H: *δ* = 3.51 and 4.87 ppm; ^13^C *δ* = 47.59 ppm). Polymer compositions and monomer conversions were determined using spectra obtained. Bruker Topspin 3.5 Software was used to process and export spectra.

#### Size Exclusion Chromatography (SEC)

An Agilent Infinity II MDS instrument equipped with differential refractive index (DRI), viscometry (VS), dual-angle light scatter (LS) and variable wavelength UV detectors (set at 309 nm) was used for all SEC analysis. The system was fitted with 2× PLgel Mixed D columns (300 × 7.5 mm) and a PLgel 5 *μ*m guard column. The eluent utilized was DMF with 5 mmol NH_4_BF_4_ additive at a flow rate of 1.0 mL·min^−1^ at 50°C. Poly(methyl methacrylate) standards (Agilent EasyVials) were used for calibration between 955 000 and 550 g·mol^−1^. Samples were prepared in the mobile phase and passed through a nylon membrane with 0.22 *μ*m pore size prior to injection. Agilent GPC/SEC Software was used to determine experimental molar mass (*M*_nSEC_), experimental molecular weight (*M*_wSEC_) and dispersity (*Đ*) of synthesized polymers.

#### Infrared (IR) Spectroscopy

Fourier-transform infrared (FTIR) spectra were acquired using a Jasco FTIR-4200 (Type A) spectrometer equipped with a PIKE MIRacleTM Single Reflection Horizontal Accessory possessing a ZnSe single reflection crystal plate (1.8 mm surface dimensions), rotating high-pressure clamp (applying maximum pressure), stainless steel crystal plate mount, and 45° angle of incidence. Analysis of dried crushed samples was completed following purging the setup with nitrogen for 30 min. Scans (100) were obtained between 4000 and 400 cm^−1^ with a resolution of 4 cm^−1^. Gain, aperture, scan speed, and filter were all set to auto. Standard source and chamber were used along with a triglycine sulfate (TGS) detector.

#### Fluorimetry

Fluorescence emission spectra were obtained using a Jasco FP-6500 fluorimeter equipped with a DC-powered 150 W xenon lamp and holographic grating with 1800 grooves mm 1 modified Rowland mount. Excitation and emission bandwidths were set to 3 nm with a response of 1 s and sensitivity set to medium. The scanning range was set from 450 to 550 nm, with an excitation wavelength selected at 494 nm, all with an accuracy of ±1.5 nm and reproducibility of ±0.3 nm. A scanning speed of 100 nm·min^−1^ was chosen and a data pitch of 1 nm.

#### Confocal Microscopy

Confocal imaging was completed using a Zeiss LSM 880 inverted microscope with 63× oil immersion objective lenses, equipped with three photomultiplier detectors (GaAsP, multialkali and BiG.2) and multichannel spectral imaging with an ultrasensitive GASP detector. The UV and VIS Laser Modules allowed selection of excitation lasers with wavelengths of 561 nm (Cy3) and 488 nm (fluorescein). Zeiss ZEN (blue edition) 2.3 lite was utilized for image collection and processing. All other imaging was completed using an Olympus CX41 microscope equipped with a UIS-2 20*x*/0.45/∞/0-2/FN22 lens (Olympus Ltd., Southend on sea, U.K.) and a Canon EOS 500D SLR digital camera. Nuclear staining was completed using NucBlue Fixed Cell ReadyProbes Reagent (Fisher Scientific); samples were fixed with 4% paraformaldehyde and sealed with ProLong Gold Antifade Mountant (Fisher Scientific).

#### Flow Cytometry

Flow cytometry was performed on a BD Influx cell sorter (BD Biosciences) running BD FACS Sortware software and equipped with 355-, 488-, 561-, and 642 nm lasers and detecting up to 24 parameters (21 fluorescence channels, two forward scatter channels, and one side scatter). Sample analysis required the use of the 488 nm excitation laser and 530/30 nm filter for fluorescein measurements and 561 nm excitation laser and 593/30 filter for Cy3 measurements. A 100 *μ*m nozzle was fitted, operating at a pressure of 20 psi (sheath) and 21.5 psi (sample). Stream and laser alignment was performed using BD Sphero Rainbow Calibration Particles (8 Peaks 3.0–3.4 *μ*m), and all sample measurements consisted of a minimum of 50 000 recorded events. Cell detachment was completed utilizing Accutase solution (Fisher Scientific) and passed through Fisherbrand Sterile Cell Strainers (Fisher Scientific) to ensure single cell analysis. Voltage settings applied ensured that untreated control cells appeared at low fluorescence emission intensities (FITC) or to ensure all treatments were present within the detection range (Cy3). FlowJo X 10.0.7r2 (Tree Star, Ashland, USA) was used for all statistical analysis and plotting of flow cytometry data.

### Polymer Synthesis

#### Polymerization of Hydroxyethyl Acrylamide (HEA) with Pentafluorophenyl 2-Dodecylthiocarbono-thioylthio)-2-methylpropionic acid (PFP-DMP)

This procedure was adapted from Gibson and co-workers.^45,58,59^ HEA (1.00 g, 8.69 mmol), PFP-DMP, and 4,4′-azobis(4-cyanovaleric acid) (ACVA) were dissolved in a 50:50 toluene:methanol solution (8 mL) at ratios presented in Table 1 to obtain 5 degrees of polymerization (DP). Mesitylene (150 *μ*L) was used as an internal reference and an aliquot was taken in CDCl_3_ for NMR analysis. The reaction mixture was stirred under N_2_ for 30 min at RT and a further 90 min at 70°C. An aliquot of the postreaction mixture was taken for NMR analysis in MeOD, allowing percentage conversion calculations. The polymer was reprecipitated into diethyl ether from methanol three times, yielding a yellow polymer product. The resulting product was dried under vacuum and an aliquot was taken for NMR analysis in MeOD. NMR percentage conversion and SEC results are presented in Table 1. ^1^H and ^19^F NMR and IR data for all DP are located in the Supporting Information,

#### Functionalization of p(HEA)-PFP_n_ with Dibenzocyclooctyne-Amine (DBCO-NH_2_) and Reduction of Thiocarbonate Moiety

PFP-p(HEA)_*n*_ (0.20 g, 1 equiv), and DBCO-NH_2_ (2 equiv) were stirred in methanol (3 mL) for 16 h. Subsequent addition of propylamine (1.5 equiv) for 2 h was used to ensure complete reduction of the thiocarbonate moiety to a thiol group. The polymer was reprecipitated into diethyl ether from methanol three times, yielding a white polymer product. The resulting product was dried under vacuum and DMF SEC analysis was completed. An aliquot was also taken for NMR analysis in MeOD. ^1^H and ^19^F NMR, SEC, and IR data for all DP are located in the Supporting Information.

#### Fluorophore Labeled DBCO-p(HEA)_*n*_

DBCO-p(HEA)_*n*_ (0.10 g, 1 equiv) and *N*-(5-fluoresceinyl)maleimide (1.3 equiv) were dissolved in DMF (1.279 mL), degassed, and left to stir for 24 h. The yellow mixture was reprecipitated into diethyl ether from methanol three times, yielding a yellow fluorescent polymer product. Fluorescein conjugation was confirmed using fluorimetry following exhaustive dialysis (Supporting Information).

### Cell Culture and Treatment

#### Cell Culture

Human Caucasian lung carcinoma cells (adenocarcinomic human alveolar basal epithelial, A549) were obtained from European Collection of Authenticated Cell Cultures (Public Health England, UK) and grown in 175 cm^2^ Nunc cell culture flasks (ThermoFisher, Rugby, UK). Ham’s F-12K (Kaighn’s) Medium (F-12K) (Gibco, Paisley, UK) was supplemented with 10% USA-origin fetal bovine serum (FBS) purchased from Sigma-Aldrich (Dorset, UK), 100 units/mL penicillin, 100 *μ*g·mL^−1^ streptomycin, and 250 ng·mL^−1^ amphotericin B (PSA) (HyClone, Cramlington, UK). A549 cells were incubated in a humidified atmosphere of 95% air and 5% CO_2_ at 37°C. General maintenance of the cell line was completed by passaging every 7 days or before reaching 90% confluency and renewing culture medium every 3–4 days. Cells were dissociated using a balanced salt solution containing trypsin (0.25%) and EDTA (1 mM) (Gibsco) and reseeded at a density of 1.87 × 10^5^ cells per 175 cm^2^ cell culture flasks.

#### Resazurin Viability Assay

To determine the effects of Ac_4_ManNAz on cell viability, aA549 cells were plated in a 12 well plate at a density of 2 × 10^4^ cells·mL^−1^ with media supplemented with varying concentrations of Ac_4_ManNAz (10–150 *μ*M) and incubated in a humidified atmosphere of 95% air and 5% CO_2_ at 37°C for 96 h. Alamar blue reagent (10% v/v in cell media) was added to both Ac_4_ManNAz treated and untreated (control) cells. Absorbance measurements were obtained at 570 and 600 nm using a BioTek Synergy HT microplate reader to monitor the reduction of resazurin to resorufin by viable cells. Cells were incubated for 4 h at 37°C and 5% CO_2_ with readings obtained every 30 min/1 h. Total cell viability was reported relative to control cells grown solely in cell culture media alone. Cytotoxicity of DBCO-p(HEA)_*n*_ was assessed using a similar protocol: first A549 cells were incubated with Ac_4_ManNAz (40 *μ*M, 96 h) and DBCO-p(HEA)_*n*_ (0.156–10 mg·mL^−1^, 2.5 h) and Alamar blue reagent was added 24 h later (10% v/v in cell media).

#### Quantitative Assessment of Ac_4_ManNAz Concentrations

A549 cells were plated in a 12 well plate at a density of 2 × 10^5^ cells·mL^−1^ with media supplemented with varying concentrations of Ac_4_ManNAz (10–150 *μ*M) and incubated in a humidified atmosphere of 95% air and 5% CO_2_ at 37°C for 96 h. Following this, cells were incubated with DBCO-Cy3 (50 *μ*M, 2.5 h), in cell media, and imaged using an Olympus CX41 microscope. Cell detachment was completed utilizing Accutase solution, and samples were submitted for flow cytometry.

#### Confocal Imaging of Ac_4_ManNAz Concentrations

A549 cells were seeded in a 12 well plate containing coverslips at a density of 2 × 10^5^ cells·mL^−1^ with media supplemented with varying concentrations of Ac_4_ManNAz (10, 50, and 100 *μ*M) and incubated in a humidified atmosphere of 95% air and 5% CO_2_ at 37°C for 96 h. Following this, cells were incubated with DBCO-Cy3 (50 *μ*M, 2.5 h), in fresh cell media, and stained with NucBlue Live Cell ReadyProbes Reagent. Confocal images were obtained of fixed samples after mounting the coverslips onto glass slides using ProLong Gold Antifade Mountant.

#### Quantitative Assessment of DBCO-pHEA_n_-Fl Concentrations and Effects over Time

A549 cells were plated in a 12 well plate at a density of 2 × 10^5^ cells·mL^−1^ with media supplemented with Ac_4_ManNAz (40 *μ*M) and incubated in a humidified atmosphere of 95% air and 5% CO_2_ at 37°C for 96 h. Following this, cells were incubated with DBCO-pHEA_*n*_-Fl polymers (1.25–20 mg·mL^−1^, 2.5 h), to determine the optimum working concentration, washed three times with DPBS and imaged using an Olympus CX41 microscope. Cell detachment was completed utilizing Accutase solution, and samples were submitted for flow cytometry. Following this, cells incubated with Ac_4_ManNAz (40 *μ*M) and DBCO-pHEA_*n*_-Fl polymers (5 and 10 mg·mL^−1^, 2.5 h) were analyzed 0–72 h postconjugation to establish polymer loss over time. Cells treated solely with DBCO-pHEA_*n*_-Fl polymers (5 and 10 mg·mL^−1^, 2.5 h) were also analyzed using flow cytometry at 0 and 8 h time points to observe nonspecific binding/uptake and its effects over time.

#### Confocal Imaging of DBCO-pHEA_n_-Fl Treated A549 Cells

A549 cells were seeded in 8 well Nunc Lab-Tek II Chamber Slides (Fisher Scientific) at a density of 6.25 × 10^4^ cell·mL^−1^ (1.25k cells per well) and incubated with media supplemented with Ac_4_ManNAz (40 *μ*M) in a humidified atmosphere of 95% air and 5% CO_2_at 37°C for 96 h. Following three washes with DPBS, cells were incubated with DBCO-pHEA_*n*_-Fl polymers (10 mg·mL^−1^, 2.5 h) in cell media and imaged 0–72 h postconjugation. To complete this, cells were stained with NucBlue Live Cell ReadyProbes Reagent, fixed with 4% paraformaldehyde and sealed with ProLong Gold Antifade Mountant. Cells treated solely with DBCO-pHEA_*n*_-Fl polymers (10 mg·mL^−1^, 2.5 h) were also imaged to observe nonspecific binding.

#### Neuraminidase Assay

A549 cells were plated in a 12 well plate at a density of 2 × 10^5^ cells·mL^−1^ with media supplemented with Ac_4_ManNAz (40 *μ*M) and incubated in a humidified atmosphere of 95% air and 5% CO_2_ at 37°C for 96 h. Following this, cells were incubated with *α*2-3,6,8,9 Neuraminidase A (25 units·mL^−1^, 1.5 h) in DPBS either before or after treatment with DBCO-pHEA_*n*_-Fl polymers (10 mg·mL^−1^, 2.5 h) in cell media. Finally, treated cells were washed three times with DPBS and imaged using an Olympus CX41 microscope. Cell detachment was completed utilizing Accutase solution, and samples were submitted for flow cytometry analysis.

## Results and Discussion

**Figure 1:**
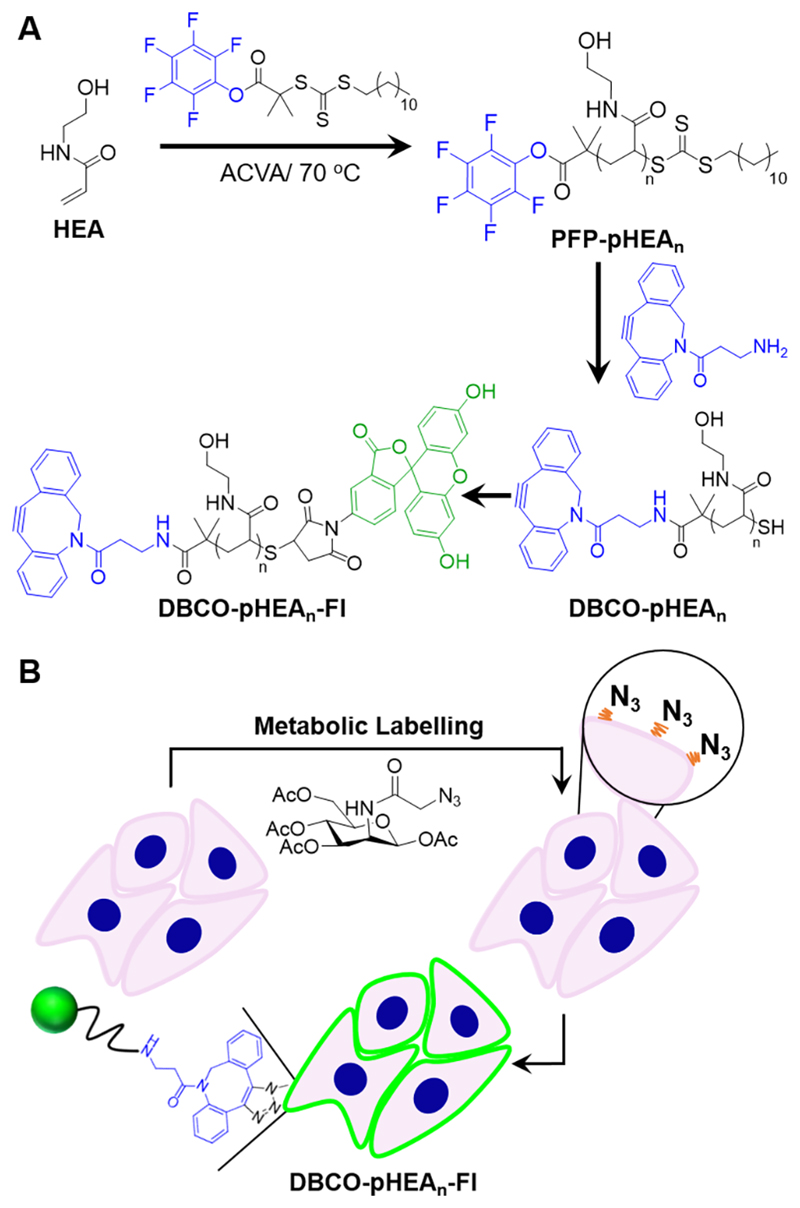
Synthetic and cell conjugation concept. (A) Synthesis of telechelic poly(*N*-hydroxyethyl acrylamide) by RAFT polymerization; (B) DBCO-pHEA*_n_*-Fl cell surface conjugation via metabolic labeling using Ac_4_ManNAz, adapted with permission from ref 45. Copyright 2018 American Chemical Society.

RAFT polymerization was used to synthesize well-defined telechelic poly(hydroxyethyl acrylamide) (pHEA) polymers bearing a pentafluorophenyl (PFP) ester and masked thiol (trithiocarbonate) end groups, Figure 1A.^46^ A library of PFP-pHEA_*n*_ polymers ranging in size (DP50–DP150) was synthesized by varying the monomer/RAFT agent ratio, Table 1. Polymers were characterized by size exclusion chromatography (SEC), ^1^H, ^13^C and ^19^F NMR, and infrared spectroscopy (IR), Table 1 and Supporting Information. Low dispersities were obtained (<1.2) in all cases and retention of the PFP group was confirmed by ^19^F NMR and IR. To introduce the biorthogonal azide-reactive functionality, PFP displacement was achieved using dibenzocyclooctyne-amine (DBCO-NH_2_).^47^ Complete PFP removal was confirmed by ^19^F NMR and IR spectroscopy (Supporting Information). To ensure complete trithiocarbonate cleavage, which is partially cleaved by DBCO-NH_2_, excess propyl amine was added revealing a thiol which was subsequently coupled to fluorescein maleimide. Thus, capitalizing on both end groups to introduce dual functionality for cell membrane attachment and imaging capabilities. Polymer/dye conjugation was confirmed using UV–vis spectroscopy and also UV–vis coupled to SEC. No free dye was observed postdialysis (Supporting Information).

**Figure 2:**
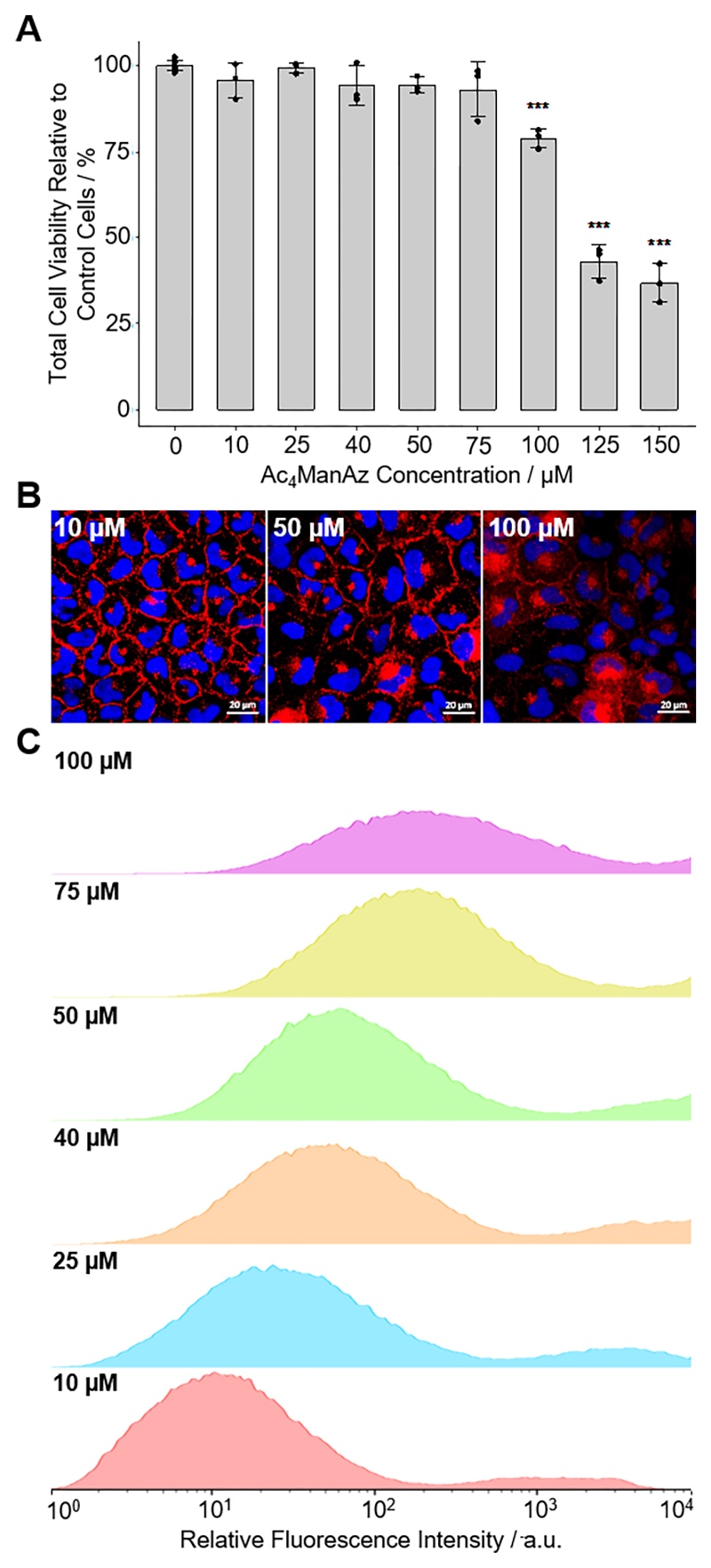
A549 cells incubated with Ac_4_ManNAz (10–150 *μ*M, 96 h) were tested for (A) cell viability using the resazurin reduction assay (*n* = 3). Cells treated with Ac_4_ManNAz (10–100 *μ*M, 96 h) and DBCO-Cy3 (50 *μ*M, 2.5 h) were analyzed using (B) confocal microscopy and (C) flow cytometry to visualize the extent of cell surface labeling. Scale bar = 20 *μ*m. ***p ≤ 0.001.

A549 (adenocarcinomic human alveolar basal epithelial) cells were incubated with Ac_4_ManNAz (peracetylated N-azidoacetylmannosamine) at varying concentrations (10–150 *μ*M), for 96 h, to enable optimization of this step in the bioconjugation workflow. Cell viability was assessed using the resazurin reduction assay, revealing an average cell viability of 95% when using less than 75 *μ*M of Ac_4_ManNAz, Figure 2A. Significant reduction in cell viability was observed at concentrations above 100 *μ*M, which was also confirmed by images showing decreased cell count and changes to cell morphology (Supporting Information). Chen et al.,^48^ confirmed Ac_4_ManNAz induces cytotoxicity of A549 cells but at higher concentrations (>500 *μ*M) through the accumulation of intracellular acetic acid and pH decrease, resulting from hydrolysis by intracellular unspecific esterases. Thus, the cytotoxicity of Ac_4_ManNAz at lower concentrations found in this study is likely due to the DMSO solvent used to solubilize the glycan, known to affect cell swelling, mechanisms, and volume changes.^49^ A resazurin reduction assay of A549 cells incubated with DMSO at exact concentrations used to solubilize Ac_4_ManNAz, for 96 h, confirmed these findings, Supporting Information.

Successful metabolic labeling of cell surface glycans was visualized using DBCO-Cyanine3 (Cy3), a fluorescent label, through strain-promoted copper-free azide–alkyne cyclo-addition, Figure 2B. Confocal imaging revealed cell surface bound fluorescence, as expected, along with some intracellular staining at higher Ac_4_ManNAz concentrations. Intracellular staining was likely due to nonspecific uptake of DBCO-Cy3 resulting in labeling of azide-mannose in the cytosol. Small hydrophobic molecule probes need to be dissolved in DMSO, which promotes this cell uptake and is one of their main disadvantages for cell tracking and labeling. Wang et al.^43^ demonstrated passive uptake of DBCO-Cy5 can occur within 30 min and worsens with increasing conjugation times. Flow cytometry confirmed these findings, revealing heterogeneous labeling of cells through broadening of the fluorescence intensity distribution, with large populations of over- and under-labeled cells, Figure 2C. Finally, confocal imaging and flow cytometry revealed insignificant differences between fluorescence emission intensity of cells treated with 40 or 50 *μ*M Ac_4_ManNAz (96 h) and DBCO-Cy3 (50 *μ*M, 2.5 h). Thus, to promote sufficient cell surface coverage of azido groups on surface bound sialic acids, and to minimize the reduction of major cellular function, A549 cells were treated with 40 *μ*M Ac_4_ManNAz for 96 h throughout this study.

**Figure 3:**
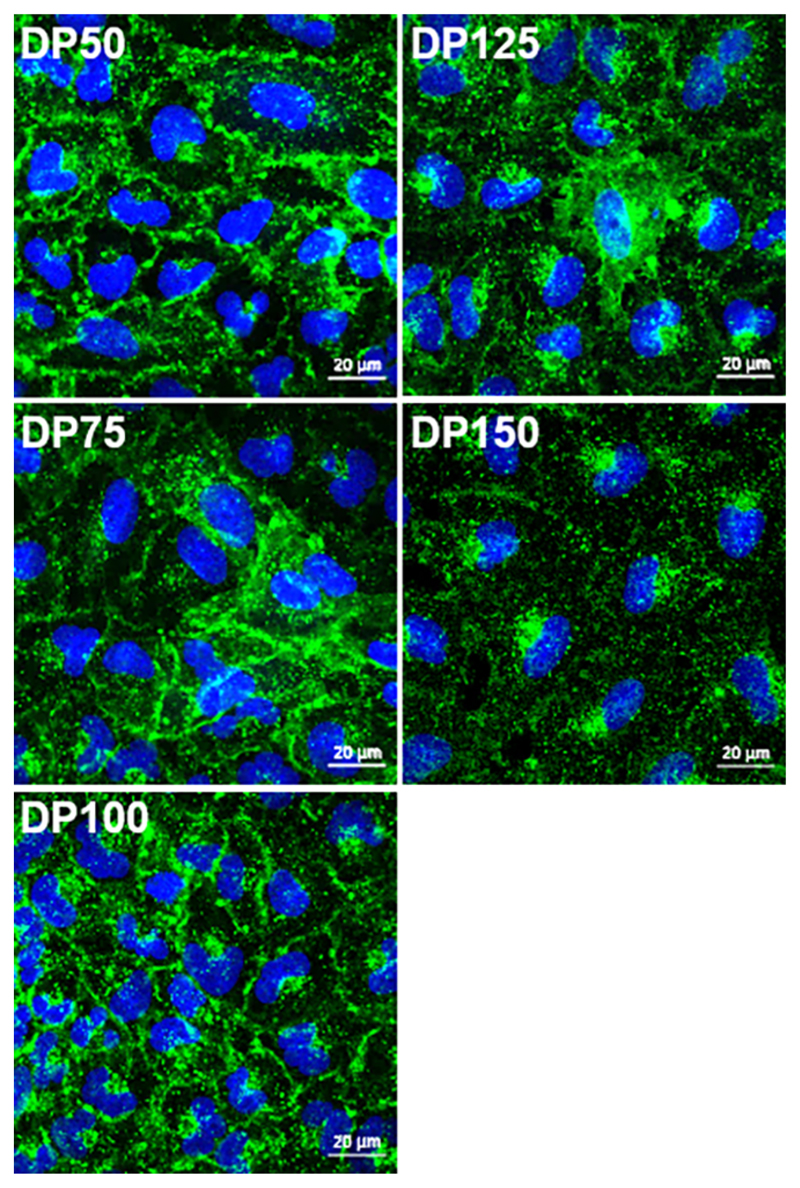
Confocal images ofA549 cells following incubation with Ac_4_ManNAz (40 *μ*M, 96 h) and DBCO-pHEA*_n_*-Fl (10 mg·mL^−1^, 2.5 h). Scale bar = 20 *μ*m.

The library of DBCO-pHEA_*n*_-Fl polymers generated was tested for azide-reactive functionality on metabolically labeled cell surface glycans at a range of concentrations (1.25–20 mg·mL^−1^), Figure 1B, enabling a complete map of grafting to be explored as a function of molecular weight, which has never been previously quantified. First, to confirm surface labeling, A549 cells were imaged following incubation with Ac_4_ManNAz (40 *μ*M, 96 h) and DBCO-pHEA_*n*_-Fl (10 mg·mL^−1^, 2.5 h), Figure 3. Cell surface bound polymers were clearly visualized through the highly fluorescent fluorescein moieties, especially for shorter polymers (DP50–DP100). Reduced grafting densities were apparent when using higher molecular weight polymers as cell surface fluorescence intensity decreased along with overall coverage. These results were attributed to lower polymer absolute (molar) concentrations and potentially steric hindrance. Remarkably, polymer coatings resided on cells undergoing proliferation suggesting potential long-term stability and was later further investigated to quantify the robustness of these modifications. Similarly to DBCO-Cy3 labeled cells, DBCO-pHEA_*n*_-Fl resulted in some localized intracellular fluorescence which was unexpected due to the polymers hydrophilic properties, avoiding the use of DMSO. Thus, an endocytosis pathway could not be ruled out. Confocal images of Ac_4_ManNAz untreated cells but still treated with DBCO-pHEA_*n*_-Fl revealed minimal internal fluorescence (Supporting Information) suggesting that endocytosis relies on polymer binding to cell surface bound azido sialic glycans. Furthermore, the majority of localized fluorescence is near or overlapping the nucleus where CMP-Neu5Az is synthesized.^50^ Thus, polymer uptake is likely caused by the sialic acid salvage pathway. During this glycan recycling process, cell surface bound sialoglycoconjugates are liberated by one of four neuraminidases and internalized via endocytosis where they are transported to lysosomes to obtain terminal sialic acid residues, converted to CMP-sialic acid, modified by the Golgi apparatus and reincorporated into the glycocalyx.^51^ This mechanism of glycan recycling provides an explanation for the localized fluorescence in the cytoplasm, its proximity to the nucleus in Ac_4_ManNAz treated cells and low nonspecific uptake in Ac_4_ManNAz untreated cells. Yamagishi et al.^52^ demonstrated that DBCO-based fluorescein dyes result in labeling of intracellular azido sugar localized in the cytosol; however, these are later (12 h) incorporated into the glycocalyx via the endoplasmic reticulum, to generate glycoproteins. Thus, we postulate that some of the intracellular azido glycan’s labeled with DBCO-pHEA_*n*_-Fl may be reincorporated into the glycocalyx.

**Figure 4:**
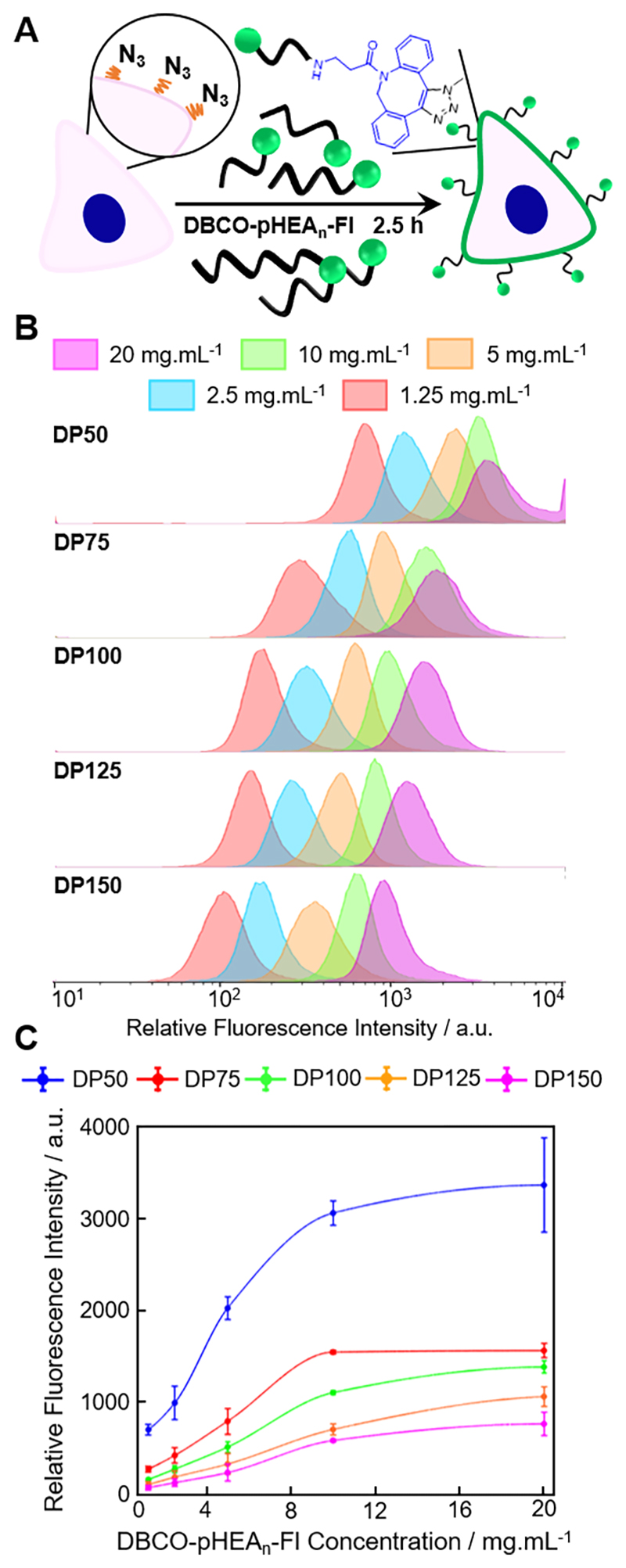
(A) A549 cells were treated with Ac_4_ManNAz (40 *μ*M, 96 h) and DBCO-pHEA*_n_*-Fl (1.25–20 mg·mL^−1^, 2.5 h) varying in length. (B) Flow cytometry analysis was used to investigate dose- and molecular weight-dependence of cell surface grafting with (C) average fluorescence intensity values plotted to reveal overall increasing trend (*n* = 3).

Polymer dose- and molecular weight-dependence on this newly explored grafting to cell surface approach have yet to be fully quantified. Thus, to provide quantitative insight into cell surface grafting densities, A549 cells, treated with Ac_4_ManNAz (40 *μ*M, 96 h) and DBCO-pHEA_*n*_-Fl (1.25–20 mg·mL^−1^, 2.5 h), were analyzed using flow cytometry, Figure 4. DP50 polymers resulted in significantly higher grafting densities at all concentrations, with a decreasing trend in grafting density noted with increasing polymer length, as expected. Despite this, all polymer lengths demonstrated sufficient cell surface coverage providing a rapid and versatile polymer grafting approach that can be fine-tuned for specific applications where molecular weight dependence has functional importance (i.e., linkers to prevent cytotoxicity of cell surface bound charged species,^53^ enhancing the activity of surface bound enzymes,^54^ attachment of large biomolecules).^55^

Confocal microscopy (Figure 3) suggested limited binding of DP150 polymers compared to DP50; however, at equal mass there are fewer end-groups (and hence fluorescein moieties). Thus, microscopy alone does not provide a complete picture. A comparison between flow cytometry results obtained of DP50 and DP100 and DP75 and DP150 polymers, at 10 mg·mL^−1^ (i.e., half molar absolute concentrations), revealed decreases in fluorescence emission intensities of 59% and 62%, respectively. Thus, proving full control of grafting density can be achieved through adjustment of polymer chain length and concentration. We postulate that the slight difference to the expected theoretical result (50%) is due to steric hindrance resulting in slower reaction kinetics, especially at lower concentrations, and less labeling of sialic acids embedded deep within the glycocalyx or proximal to already labeled sialic acids. Hawker et al.^29^ confirmed that grafting-to approaches using PEG-NHS on yeast cells suffer from heterogeneous labeling, especially at higher concentrations. Furthermore, CTA-modified yeast cells used in their cell surface-initiated polymerization grafting-from approach, resulted in fluorescence emission intensities spanning over 4 orders of magnitude. However, flow cytometry revealed that labeling of cells with DBCO-pHEA_*n*_-Fl was fairly homogeneous at all concentrations and fitted a narrow Gaussian distribution (Figure 4B), also showing a significant advantage to the small molecule probe used which resulted in large population of cells with different labeling intensities. Thus, biomolecules can be incorporated onto the surface of cells, instead of fluorescein, to provide a homogeneous population of re-engineered cells for cell-based therapies. Fluorescence emission intensities began to plateau at polymer concentrations above 10 mg·mL^−1^ for all polymer lengths. Furthermore, DP50 concentrations above 10 mg·mL^−1^ resulted in grafting densities beyond the detection range of flow cytometry. Therefore, this was the optimum maximum concentration for all further studies.

Finally, resazurin cell viability assays were completed on DBCO-pHEA_*n*_ treated A549 cells revealing an insignificant decrease in cell viability respective to polymer length (DP50–150) and concentration (0.078–10 mg·mL^−1^) (Supporting Information), demonstrating an advantage to several cytotoxic covalent conjugation^29^ and electrostatic approaches previously reported.^17^ Furthermore, cell morphology and counts appeared unaffected by polymer treatment, Supporting Information. Cytotoxicity was minimized through the use of highly water-soluble polymers and rapid kinetics of strained alkyne–azide reactions allowing conjugation in cell media, removing risks of starvation and thus exertion of unnecessary cellular stress.

**Figure 5:**
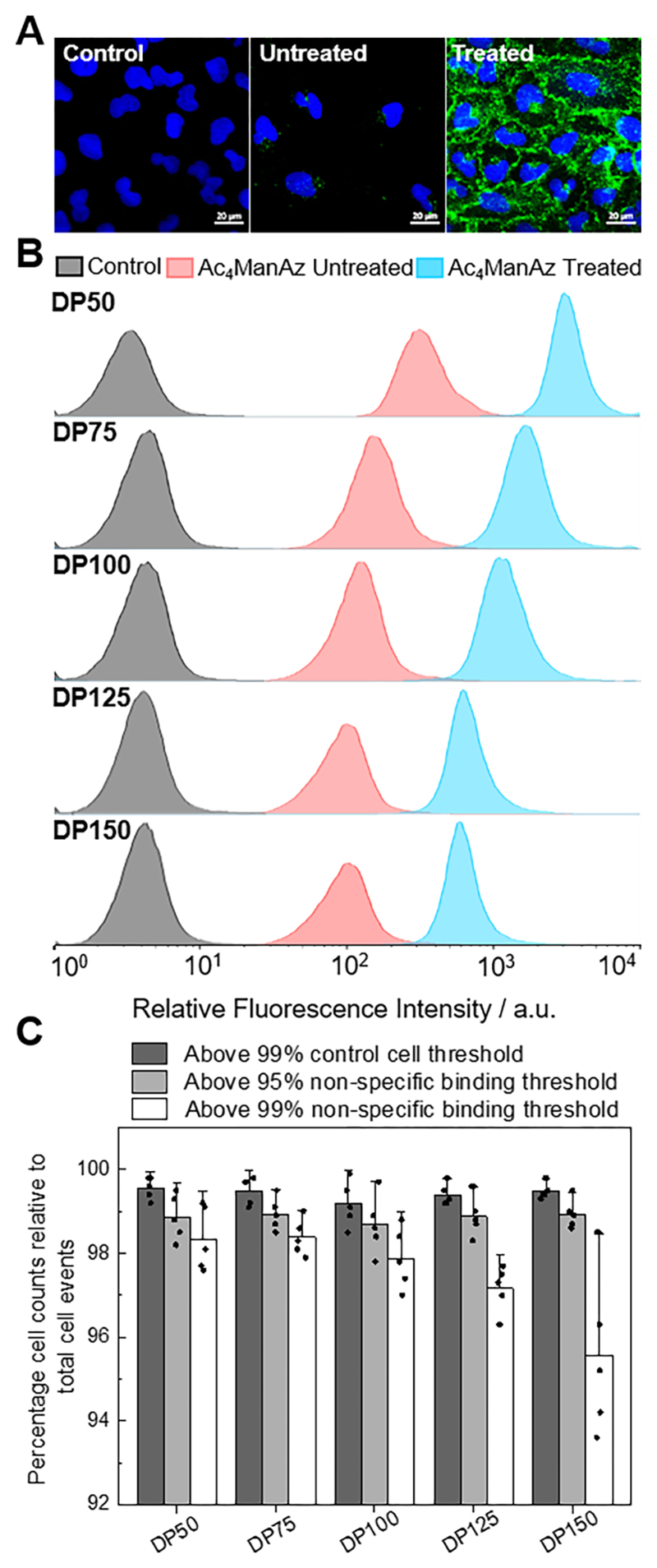
(A) Confocal images of A549 cells untreated or treated with Ac_4_ManNAz (40 *μ*M, 96 h) and treated with DBCO-pHEA_50_-Fl (10 mg·mL^−1^, 2.5 h), along with completely untreated control cells for comparison (see Supporting Information for all polymer lengths). (B) Flow cytometry analysis of all treatments was completed to quantify nonspecific binding and (C) the portion of azido–polymer treated cells with fluorescence emission intensity values above 99% of control cells and 95% or 99% of untreated Ac_4_ManNAz cells was reported (*n* = 5). Scale bar = 20 *μ*m.

The above results demonstrate controllable, dose-dependent cell surface binding of the polymers. However, for translational applications, it was crucial to quantify the extent of nonspecific binding or uptake especially as large, hydrophobic DBCO end-groups may interact or insert into the cell membrane. Confocal images of cells untreated with Ac_4_ManNAz but still treated with DBCO-pHEA_*n*_-Fl (10 mg·mL^−1^, 2.5 h) illustrated minimal nonspecific binding of all polymers (Supporting Information), even at lower polymer chain lengths (DP50), Figure 5A. To investigate this further, flow cytometry was used to quantify the fluorescence intensity of cells treated and untreated with Ac_4_ManNAz (40 *μ*M, 96 h) and incubated with DBCO-pHEA_*n*_-Fl (10 mg·mL^−1^, 2.5 h). Minimal overlap was found between cell fluorescence emission distributions of azido treated and untreated cells, confirming that DBCO-pHEA_*n*_-Fl induces minimal nonspecific binding even in the presence of various biological matrices. A significant difference was also found between the average fluorescence intensity values of azido treated and untreated cells, Supporting Information. To emphasize further the selectivity of this approach, a gating strategy was applied to determine the percentage of polymer-azido conjugated cells with fluorescence emission intensity above that of 99% of control cells (completely untreated) and 99% of azido untreated cells (polymer treated). Irrespective of polymer chain length, over 99% of azido treated cells possessed fluorescence emission intensity values above control cells and over 95% above untreated cells. Thus, demonstrating a highly efficient approach to covalently labeling cell surfaces, achieving far greater efficiency values than genetic modification techniques utilized in, e.g., CAR-T cell engineering. Previously reported values for the portion of cells labeled using other polymer conjugation methods are not available, so a more detailed comparison of conjugation efficiency could not be made. Nonspecific binding was more noticeable when treatments were completed with lower concentrations of DBCO-pHEA_*n*_-Fl (5 mg·mL^−1^, 2.5 h), especially at higher molecular weight polymers, due to lower specific grafting densities, Supporting Information.

**Figure 6:**
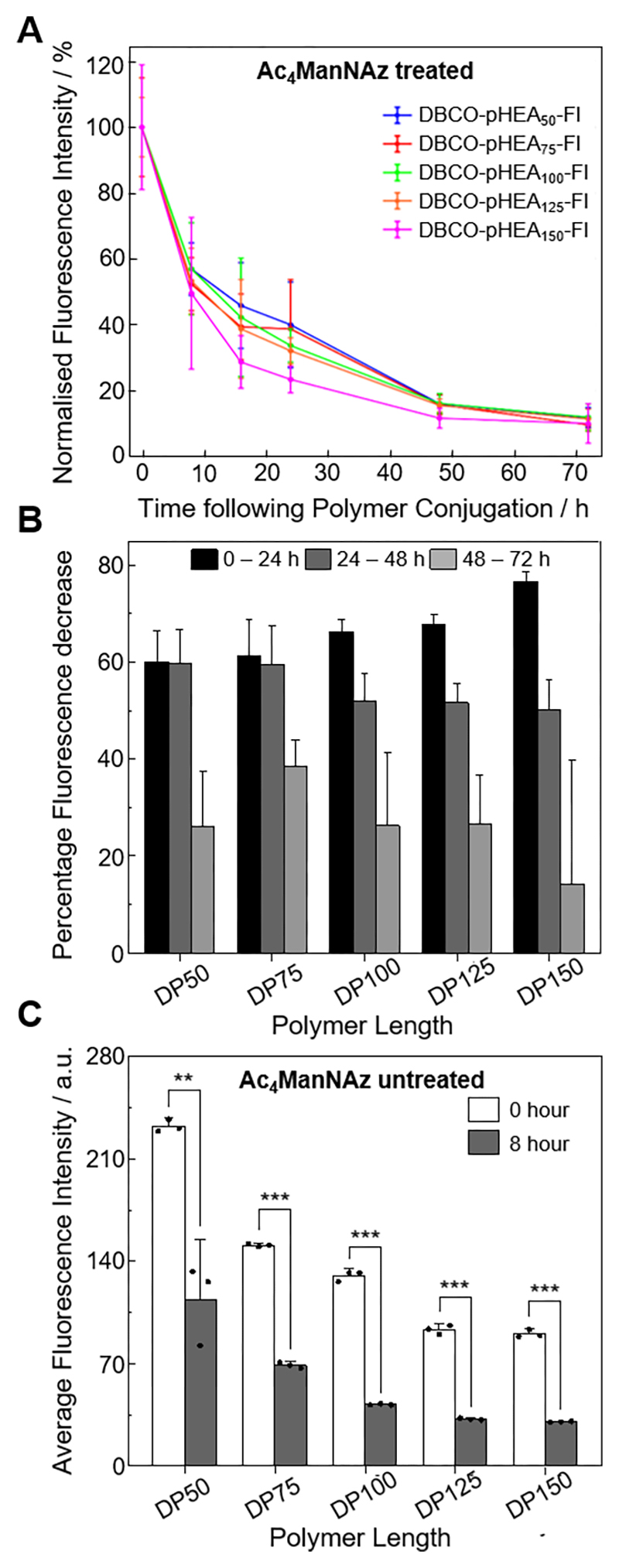
Polymer loss over time for A549 cells (A and B) treated or (C) untreated with Ac_4_ManNAz (40 *μ*M, 96 h) and treated with DBCO-pHEA_50_-Fl (10 mg·mL^−1^, 2.5 h) was quantified using flow cytometry. Fluorescence intensity of covalently bound polymer was normalized to allow direct comparison between polymer chain lengths and investigated over 72 h (*n* = 4). Decrease in nonspecifically bound polymer was investigated within the first 8 h (*n* = 3). **p ≤ 0.01. ***p ≤ 0.001.

Following confirmation that nearly all cells (>95%) were labeled using this approach, with minimal nonspecific binding, the duration of polymer attachment was investigated using flow cytometry, Figure 6A,B. A549 cells treated with both Ac_4_ManNAz (40 *μ*M, 96 h) and DBCO-pHEA_*n*_-Fl (10 mg· mL^−1^, 2.5 h) demonstrated cell surface binding for up to 72 h at all polymer chain lengths, with lower chain length polymers residing for longer due to higher grafting densities. Kang et al.^56^ confirmed that A549 cells treated with Ac_4_ManNAz result in cell surface bound azido modified sialoconjugates for 72 h, determined with a DBCO-Cy5 dye. Thus, polymer conjugation does not affect the ability for cells to retain cell surface glycans, demonstrating a completely biocompatible approach. Flow cytometry revealed extensive polymer loss within 24 h (60%–77%) of polymer conjugation, with increasing polymer length resulting in greater polymer loss. However, most nonspecifically bound polymer was detached within the first 8 h of conjugation which accounts for 6.5–13% loss in polymer, Figure 6C, which also scaled with increasing polymer length. Thus, a large majority of the polymer loss can be attributed to the *de novo* synthesis of glycans during cell division, as suggested by Baskin et al.^57^ and a small portion due to detachment of nonspecifically bound polymer. Additional mechanisms of covalently conjugated polymer loss include glycan recycling via cell surface neuraminidases confirmed by increased intracellular fluorescence in confocal images taken at 48 and 72 h time points, Supporting Information. Following 48 h postconjugation, polymer grafting density decreased by a further 50–60% in comparison to the 24 h time point, further emphasizing that cell division is most likely the main contributor to polymer loss. Less polymer loss was encountered following 72 h of incubation with high variability (14–38%) due to less polymer grafting of higher molecular weight polymer (i.e., less polymer to lose). However, the ability for polymer conjugation to persist through multiple cell division processes and other potential biological processes demonstrates a very robust approach. Polymer loss values were unaffected by polymer concentration (Supporting Information).

**Figure 7:**
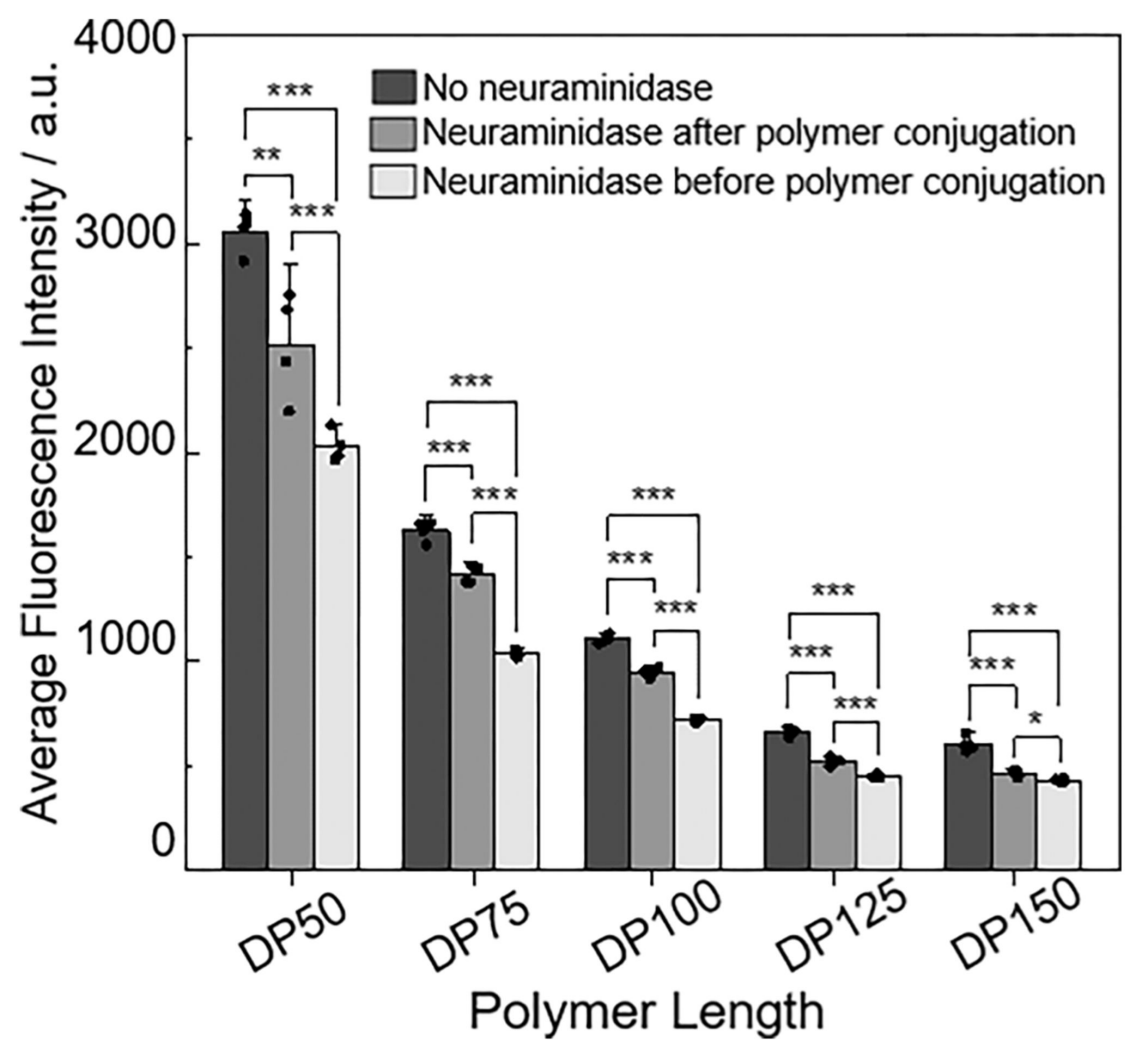
Average fluorescence intensity of *α*2-3,6,8,9 Neuraminidase A (25 *μ*·mL^−1^, 1.5 h) treated cells before or after polymer conjugation, determined using flow cytometry (*n* = 4). *p ≤ 0.05. **p ≤ 0.01. ***p ≤ 0.001.

To assess further the stability and robustness of this approach, azido labeled cells were treated with *α*2-3,6,8,9 Neuraminidase A (25 *μ*·mL^−1^, 1.5 h) either before or after polymer conjugation (Figure 7). This approach was designed to cleave linear and branched nonreducing terminal sialic acid residues from glycoproteins, glycopeptides, and oligosaccharides. Cells treated with neuraminidase before polymer conjugation resulted in 29–36% less polymer grafting, whereas neuraminidase treatment after polymer conjugation resulted in 13–24% loss of polymer. Thus, supporting the hypothesis that DBCO-pHEA_*n*_-Fl polymers target cell surface modified azido sialic acids. Enzymatic treatment following polymer conjugation resulted in less decrease in the overall fluorescence intensity, which is due to the steric bulk of the polymer on the sialic acids making it less accessible to the enzyme. Cells treated with higher molecular weight polymers showed an overall less decrease in fluorescence emission intensity, further supporting this claim. However, neuraminidase cleavage was apparent, confirming that glycan recycling is still possible and may be one of the mechanisms of polymer loss over time.

## Conclusions

Here we have reported a quantitative assessment into the grafting of synthetic polymers onto metabolically labeled cell surface glycans, using flow cytometry and confocal imaging. A library of poly(*N*-hydroxy acrylamide) bearing a strained alkyne (for copper-free azide/alkyne click) and a fluorescent reporter revealed controllable dose- and molecular weight-dependent grafting onto azide-labeled A549 cells. Cell viability was retained in all steps through the use of mild and rapid reaction conditions, a significant advantage to protein conjugation approaches. The resulting cells were covalently labeled with above 95% efficiency, resulting in narrow Gaussian-shaped fluorescence emission distributions, and demonstrating less heterogeneity compared to previously reported methods. The impact of polymer molecular weight was critically evaluated, with longer chains resulting in slightly lower grafting densities compared to shorter due to steric constraints. Covalently conjugated polymers were retained on the cells for 72 h, despite several cell division cycles, showing that the coatings are both robust and do not interfere with normal cell function. This, in particular, is an improvement over lipid-insertion methods where polymers are lost in under 24 h. Neuraminidase cleavage of cell surface sialic acids was used to prove the site of polymer conjugation. These results show that polymer attachment onto metabolically labeled glycans is a rapid, efficient, robust and versatile method to re-engineer mammalian cell surfaces without affecting cell function. The biorthogonal nature of this approach ensures that the conjugation site is controlled, as opposed to passive insertion methods or random grafting approaches targeting amines or thiols on the cells, and may be a practical alternative to genetic methods requiring only an azido (or another functional handle) bearing glycan. This methodology will enable the development of new polymer/cell hybrids and exploit the cell surface to embed tracking, therapeutic or recognition domains into polymers via a fully nongenetic approach, for future applications in the study of cell surface interactions and cell-based therapies.

## Supplementary Material

The Supporting Information is available free of charge on the ACS Publications website at DOI: 10.1021/acs.biomac.9b00478.

Experimental details, detailed polymer characterization, cytotoxicity measurements, additional flow cytometry graphs and confocal images (PDF)

Supporting information

## Figures and Tables

**Table 1:** SEC Results of PFP-pHEA*_n_*


Polymer	[M]: [CTA] (−)	% Conv.^a^	*M*_n(theo)_^b^ (g mol^−1^)	*M*_n(SEC)_^c^ (g mol^−1^)	*M*_W(SEC)_^c^ (g mol^−1^)	*Ð*^c^

PFP-pHEA_50_	50	98	6300	10200	12100	1.19
PFP-pHEA_75_	75	93	9100	13300	15800	1.19
PFP-pHEA_100_	100	95	12000	15200	18400	1.21
PFP-pHEA_125_	125	92	14900	17500	20500	1.17
PFP-pHEA_150_	150	94	17800	20200	24500	1.21

a Determined by ^1^H NMR against an internal mesitylene standard.

b Determined by the [M]:[CTA] ratio and conversion, assuming 100% CTA efficiency.

c Determined by SEC in DMF against PMMA standards.
