# Supplementary material for: Optimization and Stability of Cell–Polymer Hybrids Obtained by “Clicking” Synthetic Polymers to Metabolically Labeled Cell Surface Glycans
Source: Biomacromolecules. Author manuscript; Available in PMC 2019 Nov 5. (PMC6831485; doi:10.1021/acs.biomac.9b00478)
Supplement: Supporting information [file EMS84733-supplement-Supporting_information.pdf]

Supporting Information

Optimization and Stability of Cell-Polymer Hybrids Obtained by 'Clicking' Synthetic Polymers to Metabolically-Labeled Cell Surface Glycans

Ruben M. F. Tomás[a,c] and Matthew I. Gibson[a,b*]

[a] *Department of Chemistry, University of Warwick, Gibbet Hill Road, Coventry, CV4 7AL, UK;*

[b] *Warwick Medical School, University of Warwick, Gibbet Hill Road, Coventry, CV4 7AL, UK;*

[c]*MAS CDT, Senate House, University of Warwick, Coventry CV4 7AL*

*Corresponding Author m.i.gibson@warwick.ac.uk

1 Experimental

1.1 Materials

Ammonium fluoroborate (NH_4BF_4), dodecane thiol, tripotassium phosphate (K_3PO_4), 2-bromo-2-methylpropionic acid, dichloromethane (DCM), hydrochloric acid (HCl), magnesium sulphate ($MgSO_4$), hexane, 4-(dimethylamino) pyridine, *N*-(3-dimethylaminopropyl)-N′-ethylcarbodiimide hydrochloride, pentafluorophenol (PFP), sodium bicarbonate ($NaHCO_3$), sodium chloride (NaCl), *N*-hydroxyethyl acrylamide (HEA), 4,4′-azobis(4-cyanovaleric acid) (ACVA), toluene, methanol, mesitylene, dibenzocyclooctyne-amine (DBCO-NH_2), propyl amine, paraformaldehyde, phosphate-buffered saline preformulated tablets, dimethylformamide, N-(5-fluoresceinyl)maleimide, calcium chloride (CaCl), magnesium chloride (MgCl) and Accutase® solution were purchased from Sigma Aldrich Co Ltd (Gillingham, UK) and used without further purification.

Dulbecco phosphate buffered saline (DPBS), N-azidoacetylmannosamine-tetraacylated ($Ac_4ManNAz$), NucBlue® Fixed Cell ReadyProbes® Reagent, ProLong® Gold Antifade Mountant, Alamar blue, Nunc™ Lab-Tek™ II Chamber Slide™, Fisherbrand™ Sterile Cell Strainers, BD Sphero™ Rainbow Calibration Particles (8 Peaks 3.0-3.4 μm) and BD FACSFlow™ Sheath Fluid were purchased from Fisher Scientific (Loughborough, UK).

Human Caucasian lung carcinoma cells (A549) were obtained from European Collection of Authenticated Cell Cultures (Public Health England, UK) and grown in 175 cm^2 Nunc cell culture flasks (ThermoFisher, Rugby, UK). Ham's F-12K (Kaighn's) Medium (F-12K) (Gibco, Paisley, UK) was supplemented with 10% USA-origin fetal bovine serum (FBS) purchased from Sigma Aldrich (Dorset, UK), 100 units/mL penicillin, 100 μg/mL streptomycin, and 250 ng/mL amphotericin B (PSA) (HyClone, Cramlington, UK).

α 2-3,6,8,9 Neuraminidase A (316,000 units.mg^{-1}) purchased from NEW ENGLAND BioLabs (UK) LTD (Hitchin).

1.2 Physical and Analytical Methods

Size Exclusion Chromatography (SEC). An Agilent Infinity II MDS instrument equipped with differential refractive index (DRI), viscometry (VS), dual-angle light scatter (LS) and variable wavelength UV-Vis detectors (set at 309 nm or 494 nm) was used for all SEC analysis. The system was fitted with 2 x PLgel Mixed D columns (300 x 7.5 mm) and a PLgel 5 µm guard column. The eluent utilized was DMF with 5 mmol NH_4BF_4 additive at a flow rate of 1.0 mL.min^{-1} at 50 ^{o}C. Poly(methyl methacrylate) standards (Agilent EasyVials) were used for calibration between 955,000 – 550 g.mol^{-1}. Samples were prepared in the mobile phase and passed through a nylon membrane with 0.22 µm pore size prior to injection. Agilent GPC/SEC Software was used to determine experimental molar mass (MnSEC), experimental molecular weight (MwSEC) and dispersity (Đ) of synthesized polymers.

Nuclear Magnetic Resonance (NMR) Spectroscopy. ^{1}H, ^{13}C and ^{19}F NMR spectra were recorded on a Bruker HD-300 spectrometer at 293 K using deuterated solvents purchased from Sigma-Aldrich. Chemical shifts have been reported as δ in parts per million (ppm) relative to residual non-deuterated solvent resonances ($CDCl_3$ ^{1}H: δ = 7.26 ppm; ^{13}C δ = 77.16. CD_3OD ^{1}H: δ = 3.51 ppm and 4.87 ppm; ^{13}C δ = 47.59 ppm). Polymer compositions and monomer conversions were determined using spectra obtained. Bruker Topspin 3.5 Software was used to process and export spectra.

Infrared (IR) Spectroscopy. FTIR spectra were acquired using a Jasco FTIR-4200 (Type A) spectrometer equipped with a PIKE MIRacleTM Single Reflection Horizontal Accessory possessing a ZnSe single reflection crystal plate (1.8 mm surface dimensions), rotating high-pressure clamp, (applying maximum pressure), stainless steel crystal plate mount and 45° angle

of incidence. Analysis of dried crushed samples were completed following purging the setup with nitrogen for 30 minutes. Scans (100) were obtained between 4000 - 400 cm^{-1} with a resolution of 4 cm^{-1}. Gain, aperture, scan speed and filter were all set to auto. Standard source and chamber were used along with a triglycine sulfate (TGS) detector.

Fluorimetry. Fluorescence measurements were obtained using a Jasco FP-6500 fluorimeter equipped with a DC-powered 150 W Xenon lamp and holographic grating with 1800 grooves mm^{-1} modified Rowland mount. Excitation and emission bandwidths were set to 3 nm with a response of 1 sec and sensitivity set to medium. The scanning range was set from 450 to 550 nm, with an excitation wavelength selected at 494 nm, all with an accuracy of ±1.5 nm and reproducibility of ±0.3 nm. A scanning speed of 100 $nm.min^{-1}$ was chosen and data pitch of 1 nm.

UV-Vis Spectroscopy. An Agilent Technologies Cary 60 Variable Temperature UV-Vis spectrometer was used to record absorbance measurements between 300 and 800 nm at a scan rate of 600 $nm.min^{-1}$ and 1 nm data interval. Dual beam mode was used and the system was operated using Agilent software.

Confocal Microscopy. Confocal imaging was completed using a Zeiss LSM 880 inverted microscope with 100x, 63x, 40x and 20x oil immersion objective lenses, equipped with three photomultiplier detectors (GaAsP, multialkali and BiG.2) and multichannel spectral imaging with an ultra-sensitive GASP detector. The UV and VIS Laser Modules allowed selection of six lasers with wavelengths of 633, 594, 561, 543, 514, 488, 458, 405 and 355 nm. Zeiss ZEN (blue edition) 2.3 lite was utilized for image collection and processing. All other imaging was completed using an Olympus CX41 microscope equipped with a UIS-2 20x/0.45/∞/0−2/FN22 lens (Olympus Ltd., Southend on sea, U.K.) and a Canon EOS 500D SLR digital camera.

Flow Cytometry. Flow cytometry was performed on a BD Influx™ cell sorter (BD Biosciences) running BD FACS Sortware™ software and equipped with 355-, 488-, 561-, and 642-nm lasers and detecting up to 24 parameters (21 fluorescence channels, two forward scatter channels and one side scatter). Sample analysis required the use of the 488 nm excitation laser and 530/30 nm filter for fluorescein measurements and 561 nm excitation laser and 593/30 filter for Cy3 measurements. A 100 µm nozzle was fitted, operating at a pressure of 20 psi (sheath) and 21.5 psi (sample). Stream and laser alignment was performed using BD Sphero™ Rainbow Calibration Particles (8 Peaks 3.0-3.4 µm) and all sample measurements consisted of a minimum of 50,000 recorded events. Cell detachment was completed utilizing Accutase® solution (Fisher Scientific) and passed through Fisherbrand™ Sterile Cell Strainers (Fisher Scientific) to ensure single cell analysis. Voltage settings applied ensured that untreated control cells appeared at low fluorescence emission intensities (FITC) or to ensure all treatments were present within the detection range (Cy3). FlowJo X 10.0.7r2 (Tree Star, Ashland, USA) was used for all statistical analysis and plotting of flow cytometry data.

Statistical Analysis. Data were analyzed with a one-way analysis of variance (ANOVA) on ranks followed by a comparison of experimental groups with the appropriate control group (Tukey's post hoc test). R (R Foundation for Statistical Computing, Vienna, Austria) was used for the analyses.

1.3 General procedure for the synthesis of telechelic polymers[1–3]

Synthesis of 2-(dodecylthiocarbonothioylthio)-2-methylpropionic acid (DMP).

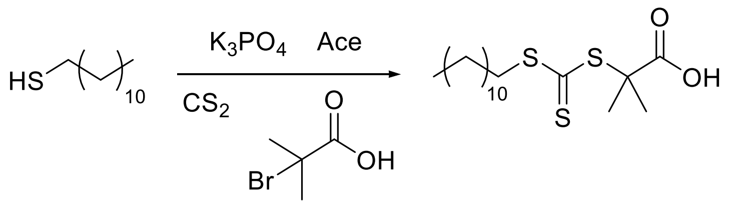

Figure S1. *Synthesis of 2-(Dodecylthiocarbonothioylthio)-2-methylpropionic acid (DMP).*

Dodecane thiol (4.00 g, 19.76 mmol) was added to a stirred suspension of K_3PO_4 (4.20 g, 19.76 mmol) in acetone, dropwise over 25 minutes. Addition of CS_2 (4.10 g, 53.85 mmol) yielded a bright yellow solution. 2-bromo-2-methylpropionic acid (3.00 g, 19.76 mmol) was added after 10 minutes of stirring. Precipitation of KBr was observed following 1 h of stirring. The solvent was removed in vacuo after 16 h and the orange residue was extracted into DCM (200 mL x 2) from HCl (1 M, 200 mL). The organic extract was washed with water (200 mL) and brine (200 mL) and dried over $MgSO_4$. The solvent was removed in vacuo and the resulting residue was dissolved in a minimal amount of hexane, heated to 65 ºC and left to recrystallize for 24 h. Subsequent removal of hexane was completed in vacuo to yield a bright yellow solid (2.78 g, 42.3%).

^{1}H NMR (300 MHz, $CDCl_3$) δ_{ppm}: 3.30 ppm (2H, t, $J_{12\text{-}11}$ = 7.73 Hz, H^{12}); 1.75 ppm (6H, br. s, H^{13}); 1.70 ppm (2H, m, H^{11}); 1.41 ppm (2H, m, H^{10}); 1.28 ppm (16H, br. s, $H^{2\text{-}9}$); 0.903 ppm (3H, t, $J_{1\text{-}2}$ = 6.88 Hz, H^1). *^{13}C NMR (300 MHz, $CDCl_3$) δ_{ppm}:* 220.84 ppm (C^{13}); 178.38 ppm (C^{16}); 37.09 ppm (C^{12}); 25.23 ppm (C^{15}); 27.82 ppm (C^{11}); 31.93 ppm, 29.64 ppm, 29.57 ppm, 29.46 ppm, 29.36 ppm, 29.12 ppm, 28.98 ppm, 22.71 ppm (C^2-C^{10}); 14.14 ppm (C^1). *^{19}F NMR (300 MHz, $CDCl_3$) δ_{ppm}:* No peaks present, as expected. *IR v /* cm^{-1}*:* 3253-2375 cm^{-1} (w, broad, carboxyl O-H stretch), 2917 cm^{-1} (m-s, CH_2 asym. stretch); 2849 cm^{-1} (m, CH_2 asym. stretch), 1712 cm^{-1} (m-s, C=O stretch (carboxylic acid)); 1281 cm^{-1} (m-s, C-O stretch); 1067 cm^{-1} (m-s, S-(C=S)-S stretch).

Synthesis of Pentafluorophenyl 2-Dodecylthiocarbonothioylthio)-2-methylpropionic acid (PFP-DMP).

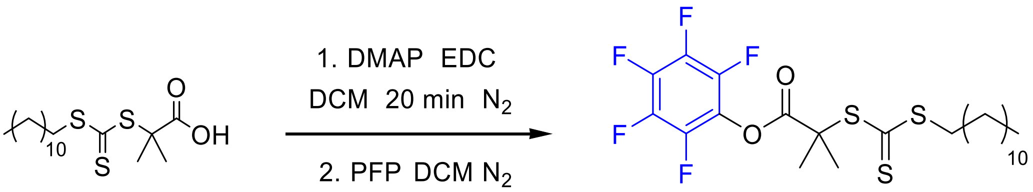

Figure S2. *Synthesis of Pentafluorophenyl 2-Dodecylthiocarbonothioylthio)-2-methylpropionic acid (PFP-DMP).*

DMP (2.00 g, 5.49 mmol), 4-(dimethylamino)pyridine (DMAP) (1.00 g, 2.05 mmol) and N-(3-Dimethylaminopropyl)-N′-ethylcarbodiimide hydrochloride (EDC) (1.56 g, 8.14 mmol) were stirred in 40 mL of DCM for 20 minutes under N_2. Pentafluorophenol (3.12 g, 16.95 mmol) dissolved in 5 mL DCM was added. The reaction was stirred at room temperature overnight. PFP-DMP isolation was achieved through successive washing of the reaction with 3 M HCl, 1 M $NaHCO_3$ and 0.5 M NaCl. The reaction was dried over $MgSO_4$, filtered and concentrated *in vacuo*.

^{1}H NMR (300 MHz, $CDCl_3$) δ_{ppm}: 3.34 (2H, t, $J^{12\text{-}11}$ = 7.26 Hz, H^{12}); 1.89 ppm (6H, s, H^{13}), 1.71 ppm (2H, q, J = 7.44 Hz, H^{11}), 1.42 ppm (2H, m, H^{10}), 1.28 ppm (16H, br. s, $H^{2\text{-}9}$), 0.903 ppm (3H, t, $J_{1\text{-}2}$ = 6.98 Hz, H^1). *^{13}C NMR (300 MHz, $CDCl_3$) δ_{ppm}:* 219.95 ppm (C^{13}), 169.65 ppm (C^{16}), 37.17 ppm (C^{12}), 27.83 ppm (C^{11}), 25.43 ppm (C^{15}), 31.92 ppm, 29.63 ppm, 29.56 ppm, 29.43 ppm, 29.35 ppm, 29.90 ppm, 28.91 ppm, 22.70 ppm (C^2-C^{10}), 14.11 ppm (C^1). *^{19}F NMR (300 MHz, $CDCl_3$) δ_{ppm}:* -151.53 ppm (2F, d, F^1, ortho), -157.75 ppm (1F, t, F^3, para), -162.35 ppm (2F, t, F^2, meta). *IR ν / cm^{-1}:* 2919 cm^{-1} (w, C-H asym. stretch); 2848 cm^{-1} (w, C-H asym. stretch); 1778 cm^{-1} (m-s, C=O stretch (ester)); 1749 cm^{-1} - 1556 cm^{-1} (w-m, ar. C-H bend); 1518 cm^{-1} (s, C-F stretch), 1082 cm^{-1} (s, C-O stretch); 1070 cm^{-1} (s, S-(C=S)-S stretch);

Polymerization of poly(hydroxylethyl acrylamide) pentafluorophenol ester

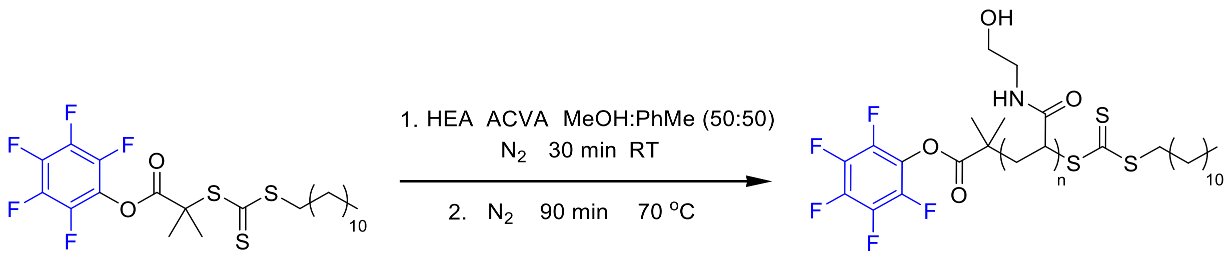

Figure S3. *Polymerisation of hydroxyethyl acrylamide using PFP-DMP.*

Hydroxyethyl acrylamide (HEA) (1.00 g, 8.69 mmol), PFP-DMP and 4,4′-azobis(4-cyanovaleric acid) (ACVA) were dissolved in a 50:50 toluene: methanol solution (8 mL) at ratios presented in Table S1 to obtain 5 degrees of polymerization (DP). Mesitylene (150 µL) was used as an internal reference and an aliquot was taken in $CDCl_3$ for NMR analysis. The reaction mixture was stirred under N_2 for 30 minutes at RT and a further 90 minutes at 70 °C. An aliquot of the post-reaction mixture was taken for NMR analysis in MeOD, allowing percentage conversion calculations. The polymer was reprecipitated into diethyl ether from methanol three times, yielding a yellow polymer product. The resulting product was dried under vacuum and an aliquot was taken for NMR analysis in MeOD. NMR percentage conversion and SEC results are presented in Table S1.

Table S1. *SEC results of PFP-pHEA$_n$.*

Polymer	[M]:[CTA] (−)	%Conv. [a]	$M_{n(theo)}$ [b] (g.mol^{-1})	$M_{n(SEC)}$ [c] (g.mol^{-1})	$M_{W(SEC)}$ [c] (g.mol^{-1})	$Đ$ [c]
PFP-pHEA$_{50}$	50	98.3	6300	10200	12100	1.19
PFP-pHEA$_{75}$	75	93.2	9200	13300	15800	1.19
PFP-pHEA$_{100}$	100	95.0	12000	17200	20800	1.21
PFP-pHEA$_{125}$	125	92.3	14900	17500	20500	1.17
PFP-pHEA$_{150}$	150	94.1	17800	20200	24500	1.21

[a] Determined by ^{1}H NMR against an internal mesitylene standard.
[b] Determined by the [M]:[CTA] ratio and conversion, assuming 100% CTA efficiency.
[c] Determined by SEC in DMF against PMMA standards.

PFP-pHEA$_{50}$: ^{1}H NMR (300 MHz, MeOD) δ_{ppm}: 3.66 ppm (br. s, HN-CH$_2$-**CH$_2$**-OH); 3.37 ppm (br. s, HN-**CH$_2$**-CH$_2$-OH); 3.13 ppm (br. s, backbone CH); 2.32-1.28 ppm (multiple br. s, backbone CH$_2$ and H$^{10-14}$); 1.20 ppm (16H, br. s, H$^{2-9}$); 0.924 ppm (3H, t, J_{1-2} = 6.88 Hz, H^1). *^{13}C NMR (300 MHz, MeOD) δ_{ppm}:* There are no clear distinct peaks, as the signal-to-noise ratio is poor. *^{19}F NMR (300 MHz, MeOD) δ_{ppm}:* -155.22 ppm (2F, m, F^1, ortho); -161.45 ppm (1F, m, F^3, para); -165.65 ppm (2F, m, F^2, meta). *IR v / cm^{-1}:* 3279 cm^{-1} (m-s, N-H stretch (secondary amine)); 3091 cm^{-1} (w, ar. C-H stretch); 2929 cm^{-1} (w, C-H asym. stretch); 2879 cm^{-1} (w, C-H asym. stretch); 1880 cm^{-1} - 1750 cm^{-1} (w (overtone), ar. C-H bend); 1643 cm^{-1} (s, C=O stretch); 1553 cm^{-1} (s, N-H bend); 1524 cm^{-1} (m, C-F stretch). 1062 cm^{-1} (s, C-O stretch); 1039 cm^{-1} (s, S-(C=S)-S stretch)

PFP-pHEA$_{75}$: ^{1}H NMR (300 MHz, MeOD) δ_{ppm}: 3.66 ppm (br. s, HN-CH$_2$-**CH$_2$**-OH); 3.37 ppm (br. s, HN-**CH$_2$**-CH$_2$-OH); 3.14 ppm (br. s, backbone CH); 2.34-1.27 ppm (multiple br. s, backbone CH$_2$ and H$^{10-14}$); 1.20 ppm (16H, t, H$^{2-9}$); 0.924 ppm (3H, t, J_{1-2} = 6.88 Hz, H^1). *^{13}C NMR (300 MHz, MeOD) δ_{ppm}:* There are no clear distinct peaks, as the signal-to-noise ratio is poor. *^{19}F NMR (300 MHz, MeOD) δ_{ppm}:* -155.25 ppm (2F, m, F^1, ortho); -161.50 ppm (1F, m, F^3, para); -165.65 ppm (2F, m, F^2, meta). *IR v / cm^{-1}:* 3283 cm^{-1} (m-s, N-H stretch (secondary amine)); 3090 cm^{-1} (w, ar. C-H stretch); 2928 cm^{-1} (w, C-H asym. stretch); 2876 cm^{-1} (w, C-H asym. stretch); 1641 cm^{-1} (s, C=O stretch); 1524 cm^{-1} (s, N-H bend); 1523 cm^{-1} (m, C-F stretch). 1062 cm^{-1} (s, C-O stretch); 1032 cm^{-1} (s, S-(C=S)-S stretch)

PFP-pHEA$_{100}$: ^{1}H NMR (300 MHz, MeOD) δ_{ppm}: 3.66 ppm (br. s, HN-CH$_2$-**CH$_2$**-OH); 3.37 ppm (br. s, HN-**CH$_2$**-CH$_2$-OH); 3.14 ppm (br. s, backbone CH); 2.32-1.27 ppm (multiple br. s, backbone CH$_2$ and H$^{10-14}$); 1.20 ppm (16H, t, J_{1-2} = 7.20 Hz, H$^{2-9}$); 0.924 ppm (3H, t, J_{1-2} = 6.77 Hz, H^1). *^{13}C NMR (300 MHz, MeOD) δ_{ppm}:* There are no clear distinct peaks, as the signal-to-noise ratio is poor. *^{19}F NMR (300 MHz, MeOD) δ_{ppm}:* -155.24 ppm (2F, m, F^1, ortho); -161.48 ppm (1F, m, F^3, para); -165.63 ppm (2F, m, F^2, meta). *IR v / cm^{-1}:* 3279 cm^{-1} (m-s, N-H stretch

(secondary amine)); 3091 cm^{-1} (w, ar. C-H stretch); 2932 cm^{-1} (w, C-H asym. stretch); 2871 cm^{-1} (w, C-H asym. stretch); 1635 cm^{-1} (s, C=O stretch); 1554 cm^{-1} (s, N-H bend); 1525 cm^{-1} (m, C-F stretch). 1061 cm^{-1} (s, C-O stretch); 1035 cm^{-1} (s, S-(C=S)-S stretch)

PFP-pHEA$_{125}$: ^{1}H NMR (300 MHz, MeOD) δ_{ppm}: 3.66 ppm (br. s, HN-CH_2-**CH_2**-OH); 3.37 ppm (br. s, HN-**CH_2**-CH_2-OH); 3.14 ppm (br. s, backbone CH); 2.31-1.29 ppm (multiple br. s, backbone CH_2 and H^{10-14}); 1.20 ppm (16H, t, J_{1-2} = 6.93 Hz, H^{2-9}); 0.923 ppm (3H, t, J_{1-2} = 6.93 Hz, H^{1}). *^{13}C NMR (300 MHz, MeOD) δ_{ppm}:* There are no clear distinct peaks, as the signal-to-noise ratio is poor. *^{19}F NMR (300 MHz, MeOD) δ_{ppm}:* -155.23 ppm (2F, m, F^{1}, ortho); -161.44 ppm (1F, m, F^{3}, para); -165.64 ppm (2F, m, F^{2}, meta). *IR v /* cm^{-1}*:* 3283 cm^{-1} (m-s, N-H stretch (secondary amine)); 3092 cm^{-1} (w, ar. C-H stretch); 2932 cm^{-1} (w, C-H asym. stretch); 2871 cm^{-1} (w, C-H asym. stretch); 1642 cm^{-1} (s, C=O stretch); 1554 cm^{-1} (s, N-H bend); 1524 cm^{-1} (m, C-F stretch). 1063 cm^{-1} (s, C-O stretch).

PFP-pHEA$_{150}$: ^{1}H NMR (300 MHz, MeOD) δ_{ppm}: 3.66 ppm (br. s, HN-CH_2-**CH_2**-OH); 3.37 ppm (br. s, HN-**CH_2**-CH_2-OH); 3.14 ppm (br. s, backbone CH); 2.34-1.27 ppm (multiple br. s, backbone CH_2 and H^{10-14}); 1.20 ppm (16H, t, J_{1-2} = 6.45 Hz, H^{2-9}); 0.923 ppm (3H, t, J_{1-2} = 6.93 Hz, H^{1}). *^{13}C NMR (300 MHz, MeOD) δ_{ppm}:* There are no clear distinct peaks, as the signal-to-noise ratio is poor. *^{19}F NMR (300 MHz, MeOD) δ_{ppm}:* -155.24 ppm (2F, m, F^{1}, ortho); -161.46 ppm (1F, m, F^{3}, para); -165.65 ppm (2F, m, F^{2}, meta). *IR v /* cm^{-1}*:* 3275 cm^{-1} (m-s, N-H stretch (secondary amine)); 3094 cm^{-1} (w, ar. C-H stretch); 2927 cm^{-1} (w, C-H asym. stretch); 2879 cm^{-1} (w, C-H asym. stretch); 1640 cm^{-1} (s, C=O stretch); 1551 cm^{-1} (s, N-H bend); 1527 cm^{-1} (m, C-F stretch). 1061 cm^{-1} (s, C-O stretch); 1034 cm^{-1} (s, S-(C=S)-S stretch).

Functionalization of p(HEA)-PFP$_n$ with DBCO-NH$_2$ and reduction of thiocarbonate moiety

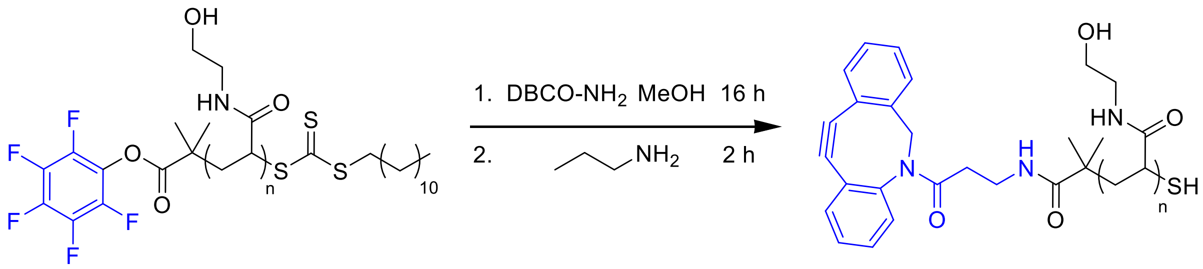

Figure S4. *Functionalisation of PFP-p(HEA) with DBCO-NH$_2$.*

PFP-p(HEA)$_n$ (0.20 g, 1 Eq), and dibenzocyclooctyne-amine (DBCO-NH$_2$; 2 Eq) were stirred in methanol (3 mL) for 16 h. Subsequent addition of propyl amine (1.5 eq) for 2 h was used to ensure complete reduction of the thiocarbonate moiety to a thiol group. The polymer was reprecipitated into diethyl ether from methanol three times, yielding a white polymer product. The resulting product was dried under vacuum and DMF SEC analysis was completed, Table S2. An aliquot was also taken for NMR analysis in MeOD.

Table S2. *SEC results of DBCO-pHEA$_n$.*

Polymer	$M_{n(theo)}$[a] (g.mol^{-1})	$M_{n(SEC)}$[b] (g.mol^{-1})	$M_{W(SEC)}$[b] (g.mol^{-1})	$Đ$[b]
DBCO-pHEA$_{50}$	6100	12500	15300	1.22
DBCO-pHEA$_{75}$	9000	19500	21300	1.09
DBCO-pHEA$_{100}$	11900	20000	25900	1.30
DBCO-pHEA$_{125}$	14800	20500	27200	1.33
DBCO-pHEA$_{150}$	17600	24100	30400	1.26

[a] Determined assuming 100% successful DBCO functionalization.
[b] Determined by SEC in DMF against PMMA standards.

DBCO-p(HEA)$_{50}$. ^{1}H NMR (300 MHz, MeOD) δ_{ppm}: 7.79 ppm – 7.03 ppm (br. m, aromatic DBCO CH); 3.66 ppm (br. s, HN-CH$_2$-**CH$_2$**-OH); 3.37 ppm (br. s, HN-**CH$_2$**-CH$_2$-OH); 3.14 ppm (br. s, backbone CH); 2.36-1.16 ppm (multiple br. s, backbone CH$_2$); 0.975 ppm (br. m,

CH_3). *^{13}C NMR (300 MHz, MeOD) δ_{ppm}:* There are no clear distinct peaks, as the signal-to-noise ratio is poor. *^{19}F NMR (300 MHz, MeOD) δ_{ppm}:* No peaks present, as expected. *IR v /* cm^{-1}*:* 3283 cm^{-1} (m-s, N-H stretch (secondary amine)); 3089 cm^{-1} (w, ar. C-H stretch); 2930 cm^{-1} (w, C-H asym. stretch); 2879 cm^{-1} (w, C-H asym. stretch); 1875 cm^{-1} - 1695 cm^{-1} (w (overtone), ar. C-H bend); 1644 cm^{-1} (s, C=O stretch); 1556 cm^{-1} (s, N-H bend)); 1253 cm^{-1} (w, DBCO C-N stretch); 1064 cm^{-1} (s, C-O stretch).

DBCO-p(HEA)$_{75}$. ^{1}H NMR (300 MHz, MeOD) δ_{ppm}: 7.80 ppm – 7.03 ppm (br. m, aromatic DBCO CH); 3.66 ppm (br. s, HN-CH_2-**CH_2**-OH); 3.33 ppm (br. s, HN-**CH_2**-CH_2-OH); 3.14 ppm (br. s, backbone CH); 2.36-1.21 ppm (multiple br. s, backbone CH_2); 0.971 ppm (br. m, CH_3). *^{13}C NMR (300 MHz, MeOD) δ_{ppm}:* There are no clear distinct peaks, as the signal-to-noise ratio is poor, of interest. *^{19}F NMR (300 MHz, MeOD) δ_{ppm}:* No peaks present, as expected. *IR v /* cm^{-1}*:* 3284 cm^{-1} (m-s, N-H stretch (secondary amine)); 3093 cm^{-1} (w, ar. C-H stretch); 2930 cm^{-1} (w, C-H asym. stretch); 2878 cm^{-1} (w, C-H asym. stretch); 1643 cm^{-1} (s, C=O stretch); 1555 cm^{-1} (s, N-H bend)); 1254 cm^{-1} (w, DBCO C-N stretch); 1063 cm^{-1} (s, C-O stretch).

DBCO-p(HEA)$_{100}$. ^{1}H NMR (300 MHz, MeOD) δ_{ppm}: 7.80 ppm – 7.03 ppm (br. m, aromatic DBCO CH); 3.66 ppm (br. s, HN-CH_2-**CH_2**-OH); 3.33 ppm (br. s, HN-**CH_2**-CH_2-OH); 3.14 ppm (br. s, backbone CH); 2.35-1.29 ppm (multiple br. s, backbone CH_2); 0.967 ppm (br. m, CH_3). *^{13}C NMR (300 MHz, MeOD) δ_{ppm}:* There are no clear distinct peaks, as the signal-to-noise ratio is poor, of interest. *^{19}F NMR (300 MHz, MeOD) δ_{ppm}:* No peaks present, as expected. *IR v /* cm^{-1}*:* 3281 cm^{-1} (m-s, N-H stretch (secondary amine)); 3087 cm^{-1} (w, ar. C-H stretch); 2929 cm^{-1} (w, C-H asym. stretch); 2875 cm^{-1} (w, C-H asym. stretch); 1645 cm^{-1} (s, C=O stretch); 1554 cm^{-1} (s, N-H bend)); 1254 cm^{-1} (w, DBCO C-N stretch); 1063 cm^{-1} (s, C-O stretch).

DBCO-p(HEA)$_{125}$. *^{1}H NMR (300 MHz, MeOD) δ$_{ppm}$:* 7.80 ppm – 7.03 ppm (br. m, aromatic DBCO CH); 3.66 ppm (br. s, HN-CH_2-**CH_2**-OH); 3.33 ppm (br. s, HN-**CH_2**-CH_2-OH); 3.14 ppm (br. s, backbone CH); 2.35-1.23 ppm (multiple br. s, backbone CH_2); 0.919 ppm (br. m, CH_3). *^{13}C NMR (300 MHz, MeOD) δ$_{ppm}$:* There are no clear distinct peaks, as the signal-to-noise ratio is poor, of interest. *^{19}F NMR (300 MHz, MeOD) δ$_{ppm}$:* No peaks present, as expected. *IR ν /* cm^{-1}*:* 3287 cm^{-1} (m-s, N-H stretch (secondary amine)); 3088 cm^{-1} (w, ar. C-H stretch); 2929 cm^{-1} (w, C-H asym. stretch); 2877 cm^{-1} (w, C-H asym. stretch); 1646 cm^{-1} (s, C=O stretch); 1552 cm^{-1} (s, N-H bend)); 1254 cm^{-1} (w, DBCO C-N stretch); 1063 cm^{-1} (s, C-O stretch).

DBCO-p(HEA)$_{150}$. *^{1}H NMR (300 MHz, MeOD) δ$_{ppm}$:* 7.80 ppm – 7.08 ppm (br. m, aromatic DBCO CH); 3.66 ppm (br. s, HN-CH_2-**CH_2**-OH); 3.33 ppm (br. s, HN-**CH_2**-CH_2-OH); 3.14 ppm (br. s, backbone CH); 2.38-1.22 ppm (multiple br. s, backbone CH_2); 0.920 ppm (br. m, CH_3). *^{13}C NMR (300 MHz, MeOD) δ$_{ppm}$:* There are no clear distinct peaks, as the signal-to-noise ratio is poor, of interest. *^{19}F NMR (300 MHz, MeOD) δ$_{ppm}$:* No peaks present, as expected. *IR ν /* cm^{-1}*:* 3287 cm^{-1} (m-s, N-H stretch (secondary amine)); 3086 cm^{-1} (w, ar. C-H stretch); 2930 cm^{-1} (w, C-H asym. stretch); 2875 cm^{-1} (w, C-H asym. stretch); 1646 cm^{-1} (s, C=O stretch); 1554 cm^{-1} (s, N-H bend)); 1254 cm^{-1} (w, DBCO C-N stretch); 1063 cm^{-1} (s, C-O stretch).

Fluorophore labeled DBCO-p(HEA)$_n$

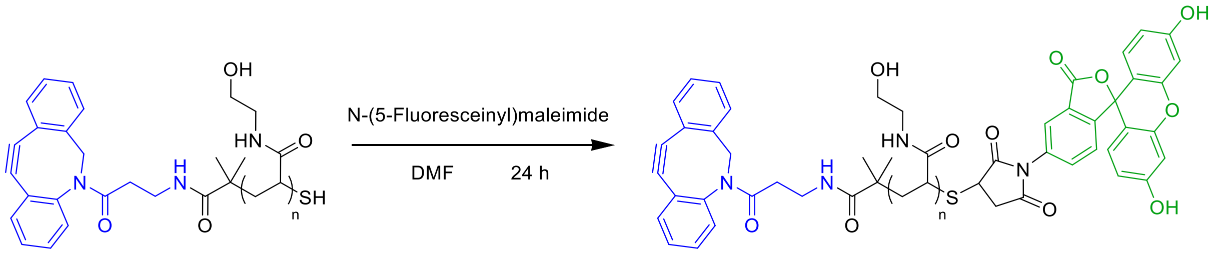

Figure S5. *Labelling DBCO-p(HEA)$_n$ with fluorescein.*

DBCO-p(HEA)$_n$ (0.10 g, 1 Eq), and *N*-(5-fluoresceinyl)maleimide (1.3 Eq) were dissolved in DMF (1.279 mL), degassed and left to stir for 24 h. The yellow mixture was reprecipitated into diethyl ether from methanol three times, yielding a yellow fluorescent polymer product. Fluorescein conjugation was confirmed using fluorimetry following exhaustive dialysis. DMF SEC analysis was completed with the UV-Vis detector set at 494 nm to demonstrate size separation and absorbance overlap, Table S3 and Fig. S12. Percentage dye functionalisation was determined using UV-Vis spectroscopy, Table S3 and Fig. S14 and S15. Deviations from expected Polymer: dye ratio (i.e. 1:1) is expected to be because of error in determining exact molecular weight using SEC.

Table S3. *SEC and fluorimetry results of DBCO-pHEA$_n$-Fl.*

Polymer	$M_{n(theo)}$[a] (g.mol^{-1})	$M_{n(SEC)}$[b] (g.mol^{-1})	$M_{W(SEC)}$[b] (g.mol^{-1})	$Đ$[b]	*Polymer: dye*[c]
PFP-pHEA$_{50}$	6600	17900	21800	1.23	1 : 1.28
PFP-pHEA$_{75}$	9400	22300	26600	1.19	1 : 0.94
PFP-pHEA$_{100}$	12300	25000	30400	1.22	1 : 0.99
PFP-pHEA$_{125}$	15200	31700	39400	1.24	1 : 1.58
PFP-pHEA$_{150}$	18100	34400	42300	1.23	1 : 1.31

[a] Determined by assuming 100% successful dye functionalization.
[b] Determined by SEC in DMF against PMMA standards.
[c] Determined by absorbance measurements recorded using UV-Vis spectroscopy.

1.4 Cell Culture

Cell culture. Human Caucasian lung carcinoma cells (A549) were obtained from European Collection of Authenticated Cell Cultures (Public Health England, UK) and grown in 175 cm^2 Nunc cell culture flasks (ThermoFisher, Rugby, UK). Ham's F-12K (Kaighn's) Medium (F-12K) (Gibco, Paisley, UK) was supplemented with 10% USA-origin fetal bovine serum (FBS) purchased from Sigma Aldrich (Dorset, UK), 100 units/mL penicillin, 100 µg/mL

streptomycin, and 250 ng/mL amphotericin B (PSA) (HyClone, Cramlington, UK). A549 cells were incubated in a humidified atmosphere of 95% air and 5% CO_2 at 37 °C. General maintenance of the cell line was completed by passaging every 7 days or before reaching 90% confluency and renewing culture medium every 3 – 4 days. Cells were dissociated using a balanced salt solution containing trypsin (0.25%) and EDTA (1 mM) (Gibsco) and reseeded at a density of 1.87 x 10^5 cells per 175 cm^2 cell culture flasks.

1.5 Optimum peracetylated N-azidoacetylmannosamine ($Ac_4ManNAz$) concentration

Cytotoxicity. A549 cells were seeded in a 96 well plate at a density of 6.25k cell.mL^{-1} (1.25k cells per well) and incubated with media supplemented with varying concentrations of $Ac_4ManNAz$ (10 – 150 µM) in a humidified atmosphere of 95% air and 5% CO_2 at 37 °C for 96 h. Following three washes with DPBS, cell viability was determined by adding Alamar blue reagent (10% v/v in cell media) to $Ac_4ManNAz$ treated and untreated (control) cells. Absorbance measurements were obtained at 570 nm and 600 nm using a BioTek Synergy HT microplate reader to monitor the reduction of resazurin to resorufin by viable cells. Cells were incubated for 4 h at 37 ºC and 5% CO_2 with readings obtained every 30 min / 1 h. Total cell viability was reported relative to control cells grown solely in cell culture media alone.

Flow Cytometry. A549 cells were plated in a 12 well plate at a density of 2 x 10^5 cells.mL^{-1} with media supplemented with varying concentrations of $Ac_4ManNAz$ (10 – 150 µM) and incubated in a humidified atmosphere of 95% air and 5% CO_2 at 37 °C for 96 h. Following this, cells were incubated with DBCO-Cy3 (50 µM, 2.5 h), in cell media, and imaged using an Olympus CX41 microscope. Cell detachment was completed utilizing Accutase® solution and strained using nylon cell strainers (40 µm) to ensure single cell analysis. BD Influx™ cell sorter (BD Biosciences) operating parameters were as described above. Voltage settings were selected to ensure all concentrations were present within the detection limits.

Confocal Imaging. A549 cells were seeded in a 12 well plate containing coverslips at a density of 2 x 10^5 cells.mL^{-1} with media supplemented with varying concentrations of $Ac_4ManNAz$ (10, 50 and 100 μM) and incubated in a humidified atmosphere of 95% air and 5% CO_2 at 37 °C for 96 h. Following this, cells were incubated with DBCO-Cy3 (50 μM, 2.5 h), in fresh cell media, and stained with NucBlue® Live Cell ReadyProbes® Reagent. Confocal images were obtained of fixed samples after mounting the coverslips onto glass slides using ProLong® Gold Antifade Mountant.

1.6 Optimum DBCO-pHEA$_n$-Fl concentration and degrafting assessment

Cytotoxicity. A549 cells were seeded in a 96 well plate at a density of 6.25k cell.mL^{-1} (1.25k cells per well) and incubated with media supplemented with $Ac_4ManNAz$ (40 μM) in a humidified atmosphere of 95% air and 5% CO_2 at 37 °C for 96 h. Cells were subsequently incubated with DBCO-pHEA$_n$-Fl polymers (0.156 - 10 mg.mL^{-1}, 2.5 h), washed three times with DBPS and incubated with fresh media for 24 h. Cell viability of DBCO-pHEA$_n$-Fl treated and untreated (control) cells were measured using Alamar blue (10% v/v in cell media) as described previously.

Flow Cytometry. A549 cells were plated in a 12 well plate at a density of 2 x 10^5 cells.mL^{-1} with media supplemented with $Ac_4ManNAz$ (40 μM) and incubated in a humidified atmosphere of 95% air and 5% CO_2 at 37 °C for 96 h. Following this, cells were incubated with DBCO-pHEA$_n$-Fl polymers (1.25 - 10 mg.mL^{-1}, 2.5 h), washed three times with DPBS and imaged using an Olympus CX41 microscope. Cell detachment was completed utilizing Accutase® solution and cells were strained using nylon cell strainer (40 μm) to ensure single cell analysis. BD Influx™ cell sorter (BD Biosciences) operating parameters were as described above. Control cells, untreated with $Ac_4ManNAz$, were also incubated with DBCO-pHEA$_n$-Fl

polymers (5 and 10 mg.mL^{-1}, 2.5 h) and analyzed using flow cytometry and microscopy to determine the extent of non-specific binding.

Confocal Imaging. A549 cells were seeded in 8 well Nunc™ Lab-Tek™ II Chamber Slides™ at a density of 6.25k cell.mL^{-1} (1.25k cells per well) and incubated with media supplemented with Ac_4ManNAz (40 µM) in a humidified atmosphere of 95% air and 5% CO_2 at 37 °C for 96 h. Following three washes with DPBS, cells were incubated with DBCO-pHEA$_n$-Fl polymers (10 mg.mL^{-1}, 2.5 h). Control cells, untreated with Ac_4ManNAz, were also incubated with DBCO-pHEA$_n$-Fl polymers (10 mg.mL^{-1}, 2.5 h) to determine the extent of non-specific binding. Cells were subsequently stained with NucBlue® Live Cell ReadyProbes® Reagent, fixed with 4% paraformaldehyde and sealed with ProLong® Gold Antifade Mountant.

1.7 DBCO-pHEA$_n$-Fl cell surface degrafting

Flow Cytometry. A549 cells were plated in a 12 well plate at a density of 2 x 10^5 cells.mL^{-1} with media supplemented with Ac_4ManNAz (40 µM) and incubated in a humidified atmosphere of 95% air and 5% CO_2 at 37 °C for 96 h. Following this, cells were incubated with DBCO-pHEA$_n$-Fl polymers (5 or 10 mg.mL^{-1}, 2.5 h) in fresh cell media, washed three times with DPBS and imaged using an Olympus CX41 microscope 0 - 72 h post conjugation. Flow sample preparation and operating parameters were completed as previously described above. Control cells, untreated with Ac_4ManNAz, were also incubated with DBCO-pHEA$_n$-Fl polymers (5 and 10 mg.mL^{-1}, 2.5 h) and analyzed using flow cytometry and microscopy at 0 h and 8 h post conjugation to determine the extent of degrafting of non-specific binding.

Confocal Imaging. A549 cells were seeded in 8 well Nunc™ Lab-Tek™ II Chamber Slides™ at a density of 6.25k cell.mL^{-1} (1.25k cells per well) and incubated with media supplemented with Ac_4ManNAz (40 µM) in a humidified atmosphere of 95% air and 5% CO_2 at 37 °C for 96 h. Following three washes with DPBS, cells were incubated with DBCO-pHEA$_n$-Fl polymers

(10 mg.mL^{-1}, 2.5 h) in cell media. Confocal imaging was completed on cells treated 0 – 72 h post conjugation. To complete this, cells were stained with NucBlue® Live Cell ReadyProbes® Reagent, fixed with 4% paraformaldehyde and sealed with ProLong® Gold Antifade Mountant.

1.8 Neuraminidase assay

Flow Cytometry. A549 cells were plated in a 12 well plate at a density of 2 x 10^5 cells.mL^{-1} with media supplemented with $Ac_4ManNAz$ (40 μM) and incubated in a humidified atmosphere of 95% air and 5% CO_2 at 37 °C for 96 h. Following this, cells were incubated with α 2-3,6,8,9 Neuraminidase A (25 units.mL^{-1}, 1.5 h) in DPBS either before or after treatment with DBCO-pHEA$_n$-Fl polymers (10 mg.mL^{-1}, 2.5 h) in cell media. Finally, treated cells were washed three times with DPBS and imaged using an Olympus CX41 microscope. Flow sample preparation and operating parameters were completed as previously described above.

2 Additional Results

2.1 Polymer Characterisation

2.1.1 DMP and PFP-DMP

DMP conversion to PFP-DMP was evident by the presence of peaks in the ^{19}F NMR spectrum of PFP-DMP corresponding to the addition of a pentafluorophenol moiety, which were absent for DMP, Figure S7. Furthermore, the disappearance of a broad carboxylic acid O-H stretch (3253-2375 cm^{-1} (w, broad)) and a shift in C=O stretch confirmed the conversion of a carboxylic acid to an ester (1778 cm^{-1}, m-s), Figure S6. Finally, a C-F stretch (1518 cm^{-1}, s) was noted in the IR of PFP-DMP.

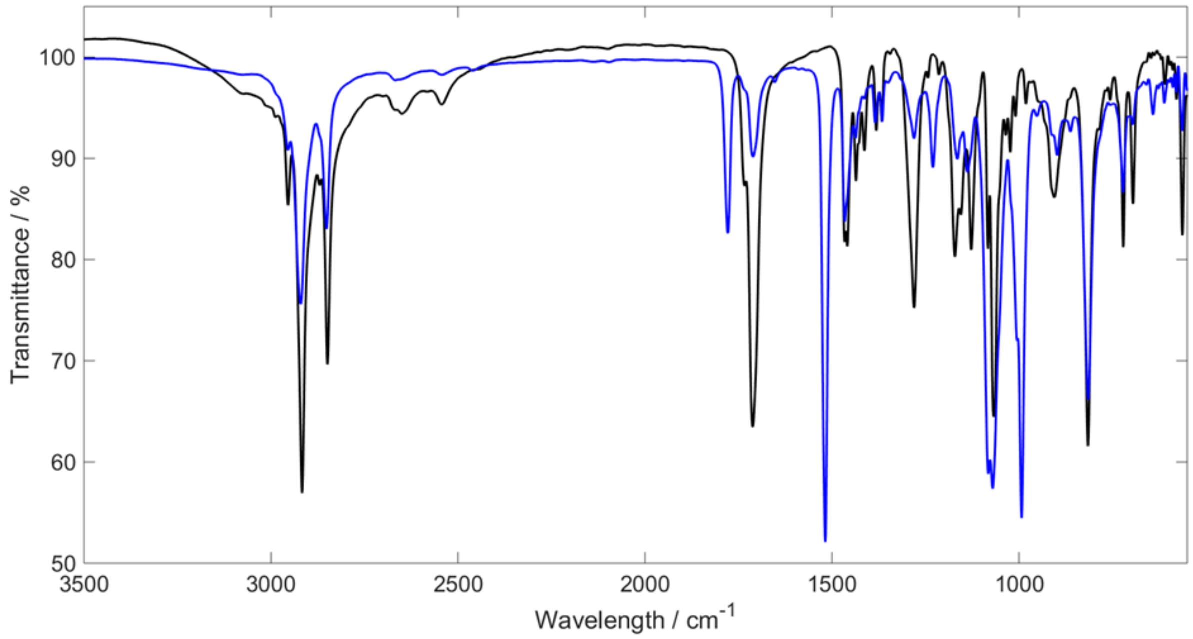

Figure S6. *Infrared Spectrum of DMP (black) and PFP-DMP (blue).*

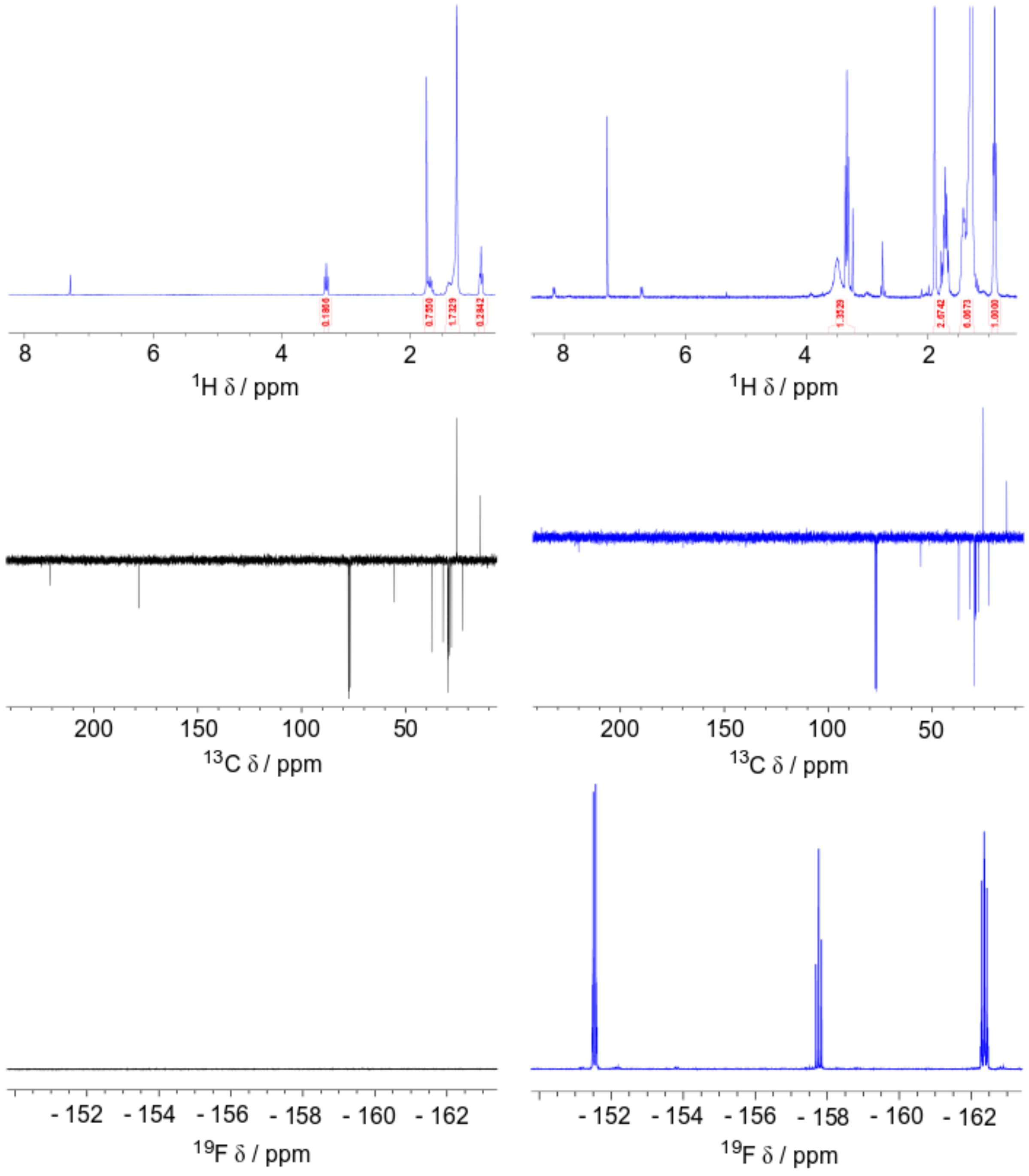

Figure S7. *^{1}H, APT ^{13}C and ^{19}F NMR spectra of DMP (black) and PFP-DMP (blue).*

2.1.2 *p(HEA)$_n$-PFP and p(HEA)$_n$-DBCO*

p(HEA)$_n$-PFP and p(HEA)$_n$-DBCO polymers were fully characterized using ^{1}H and ^{19}F NMR spectroscopy (Figure S8 and S9), IR spectroscopy (Figure S10) and SEC (Figure S10). SEC confirmed the synthesis of polymers of five different chain lengths. Thiocarbonate conversion to thiol was evident by removal of ^{1}H NMR peaks associated to the connected $(CH_2)_{11}CH_3$ chain (1.20 ppm (16H, t, H^{2-9}); 0.924 ppm (3H, t, J_{1-2} = 6.88 Hz, H^1). Removal of a S-(C=S)-S stretch (1030 cm^{-1}, s) in IR spectra of p(HEA)$_n$-DBCO provided further confirmation. Removal of PFP was established via the absence of peaks in ^{19}F NMR and C-F stretch (1520 cm^{-1}, m) in IR. Addition of a DBCO moiety was confirmed by the presence of aromatic DBCO CH peaks in ^{1}H NMR (7.80 ppm – 7.00 ppm) and DBCO C-N stretch (1255 cm^{-1}, w) in IR spectroscopy. The hydrophobic nature of DBCO results in increases to MW and dispersity readings due to non-specific hydrophobic secondary interaction with the SEC stationary phase, providing further confirmation of functionalization.

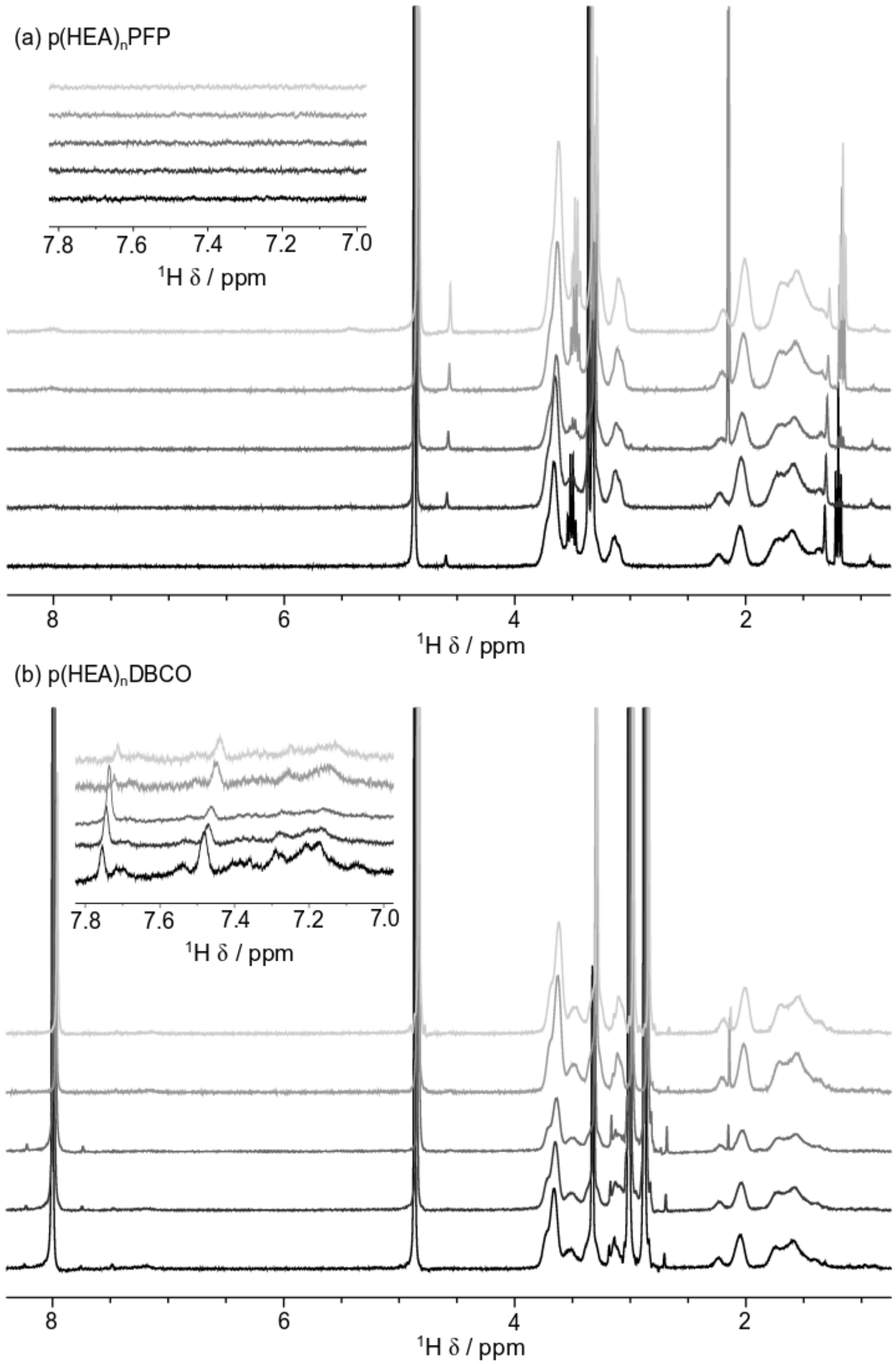

Figure S8. *^1H NMR of p(HEA)$_n$ before and after functionalization with DBCO. Five different polymer lengths were synthesized (DP50, DP75, DP100, DP125, and DP150) represented by increasing grayscale.*

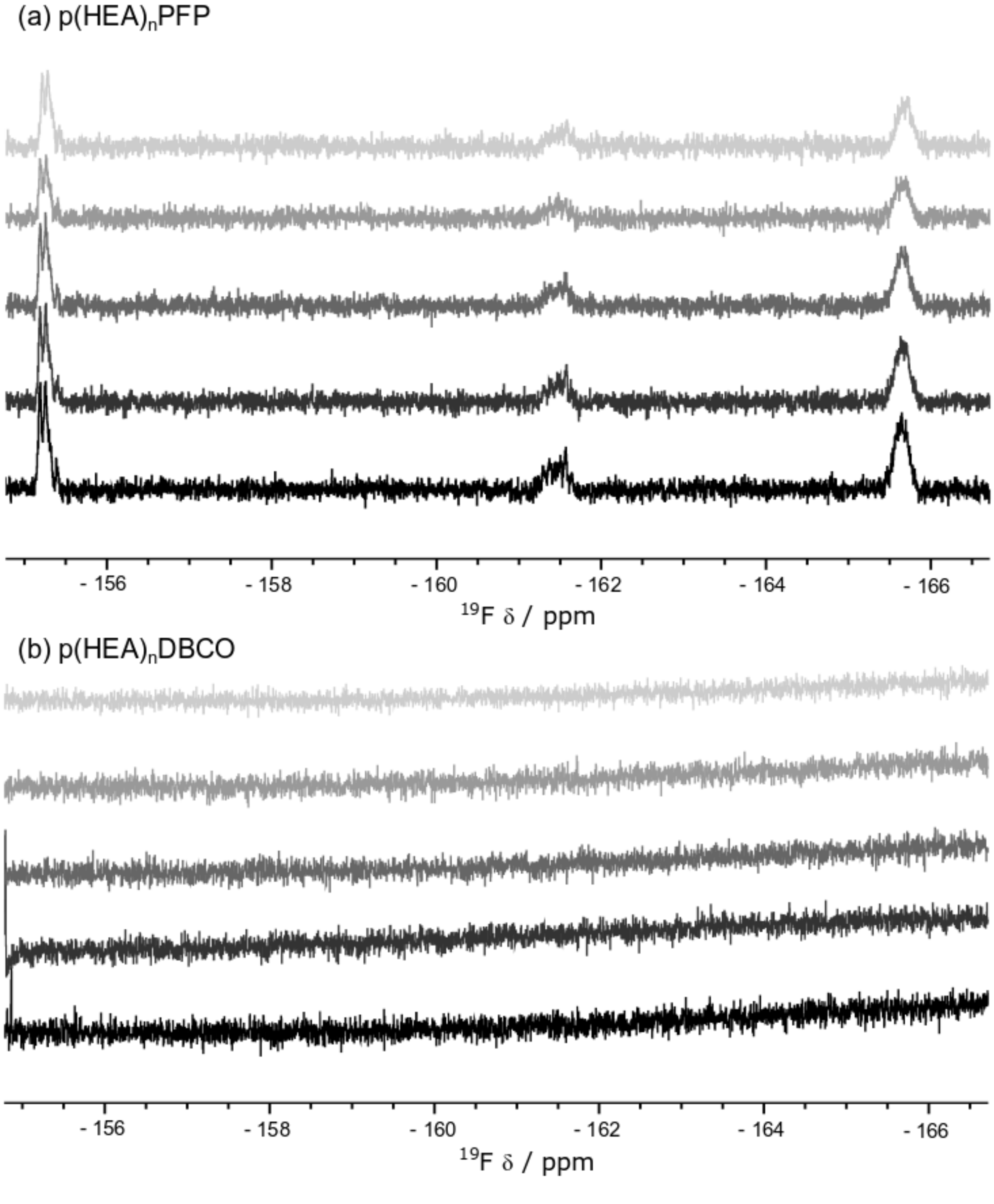

Figure S9. *^{19}F NMR of p(HEA)$_n$ before and after functionalization with DBCO. Five different polymer lengths were synthesized (DP50, DP75, DP100, DP125, and DP150) represented by increasing grayscale.*

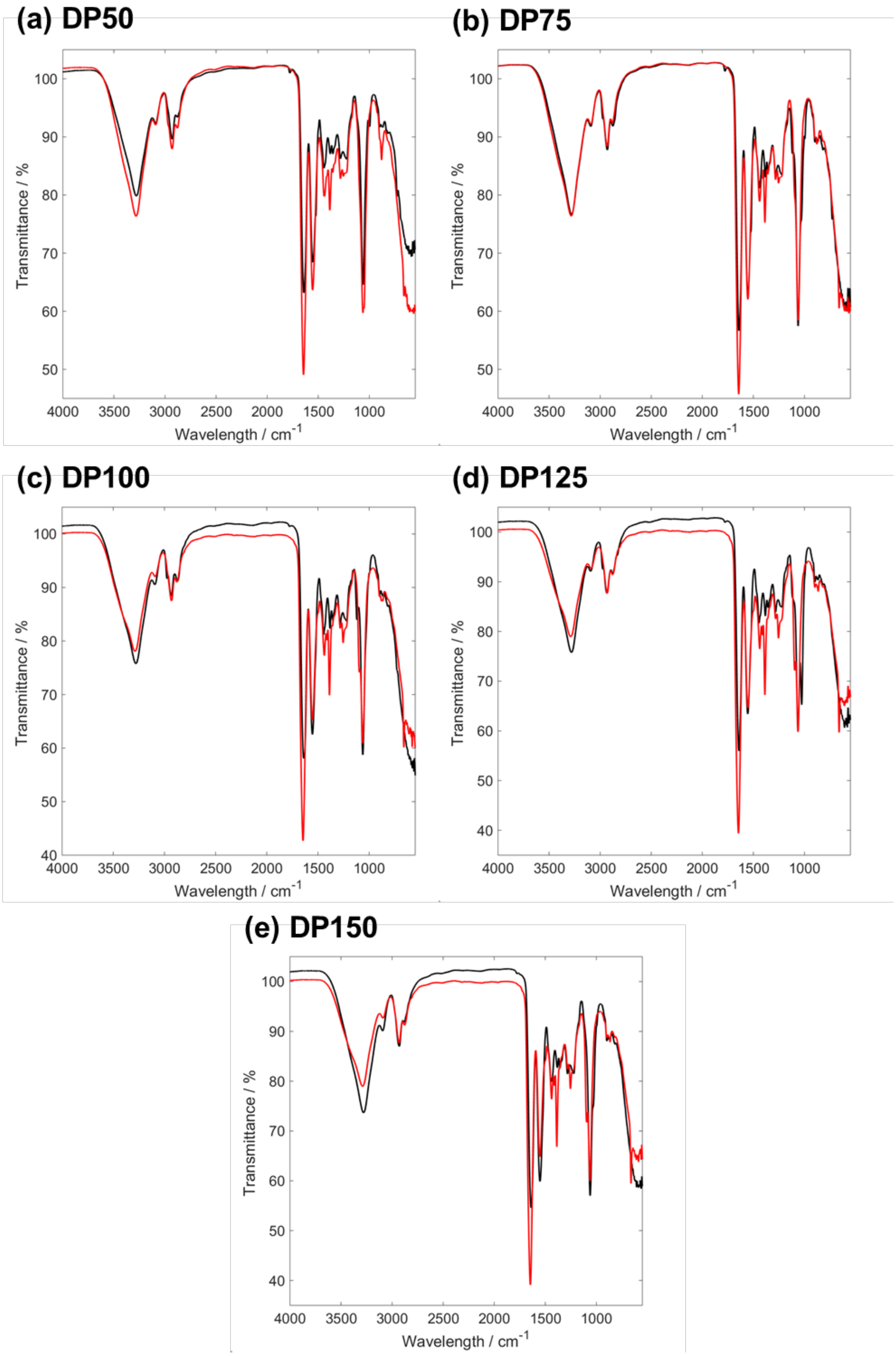

Figure S10. *Infrared spectroscopy of p(HEA)$_n$ before (black) and after (red) functionalization with DBCO.*

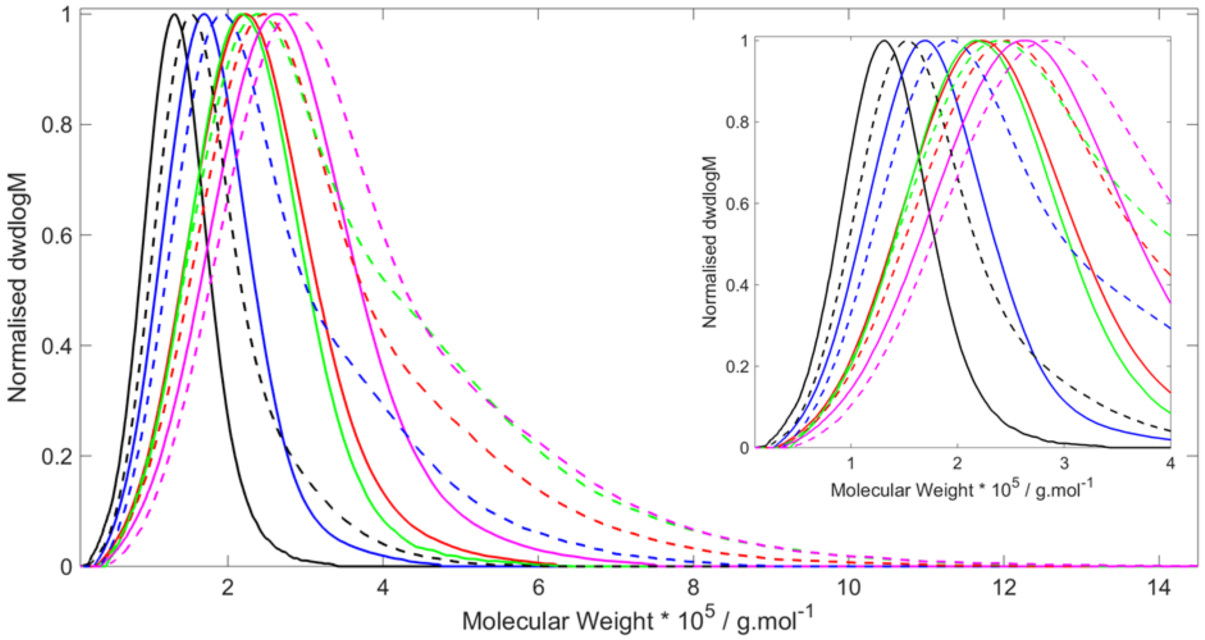

Figure S11. *SEC of p(HEA)$_n$ before (solid line) and after (dashed line) DBCO functionalization. P(HEA)$_n$ of five different sizes were synthesised, DP50 (black), DP75 (blue) DP100 (green), DP125 (red) and DP150 (magenta).*

2.1.3 *DBCO-p(HEA)$_n$-Fl*

Confirmation of fluorescein conjugation was made evident by: (1) overlap between DMF SEC results using both an RI and photodiode array detector indicating that both size and absorbance readings correlate (i.e. polymer and dye conjugation was successful) and (2) the increase in fluorescence intensity compared to the negligible fluorescence intensity found in p(HEA)$_n$-DBCO polymers, Figure S14. Polymer: dye ratio was calculated using UV-Vis spectroscopy, Fig. S15 and S16. Deviation from expected 1:1 ratio is likely due to deviations in determining the exact molecular weight of polymers using SEC.

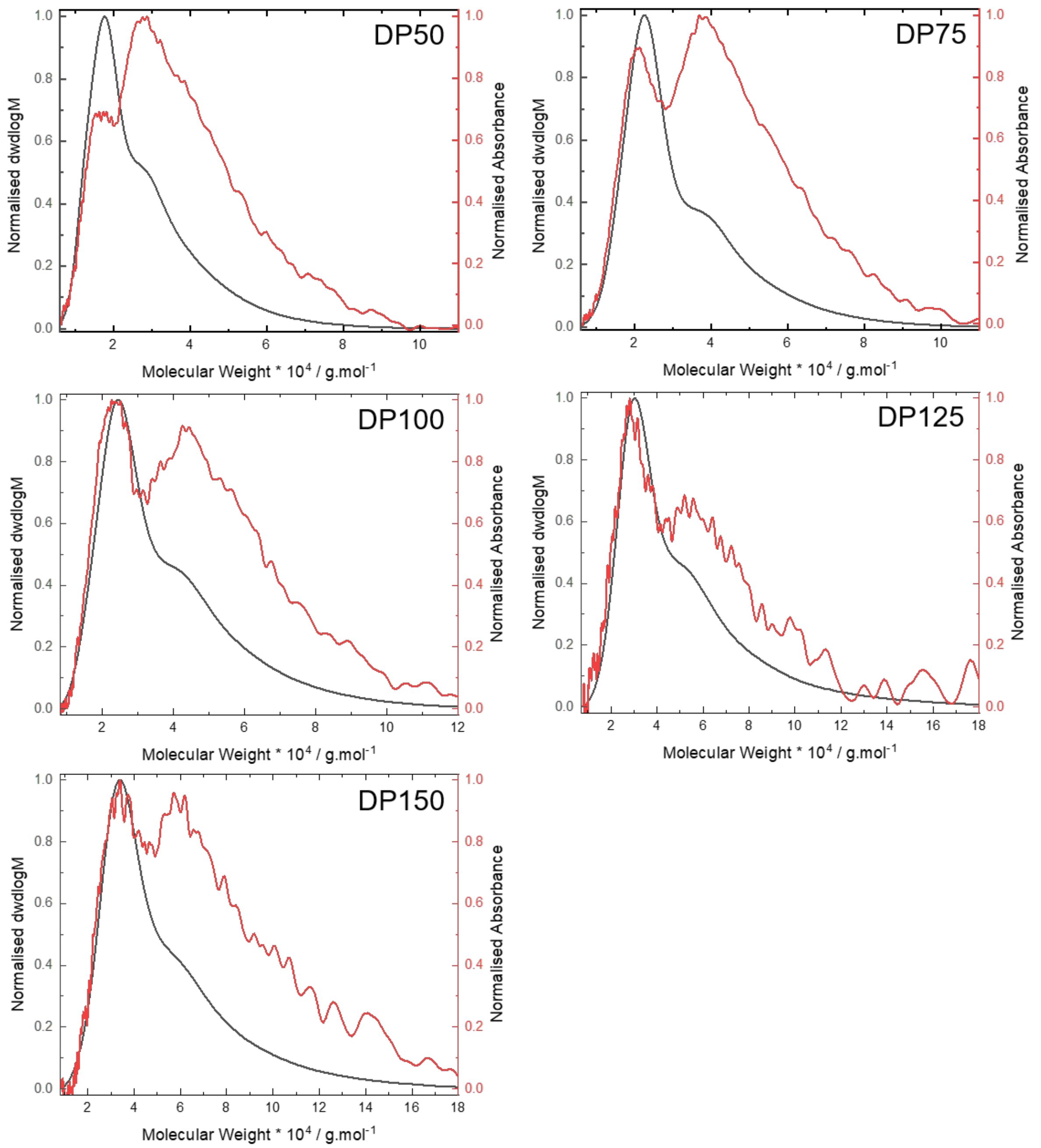

Figure S12. *DMF SEC results of DBCO-p(HEA)$_n$-Fl with molecular weight separation represented using an RI (Black) and photodiode array detector set at 494 nm (red).*

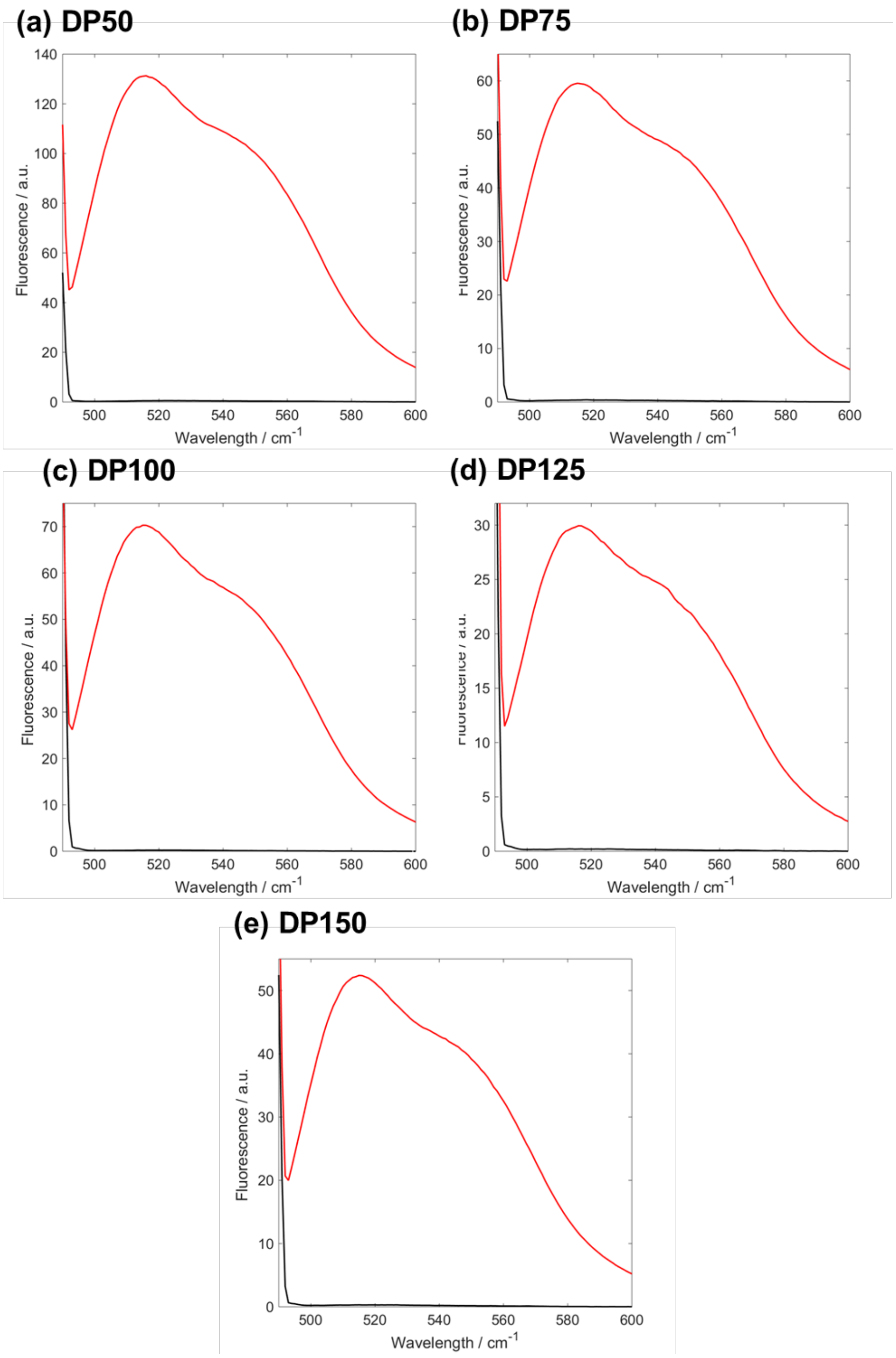

Figure S13. *The fluorescence spectrum of p(HEA)$_n$-DBCO before (black) and after (red) functionalization with fluorescein.*

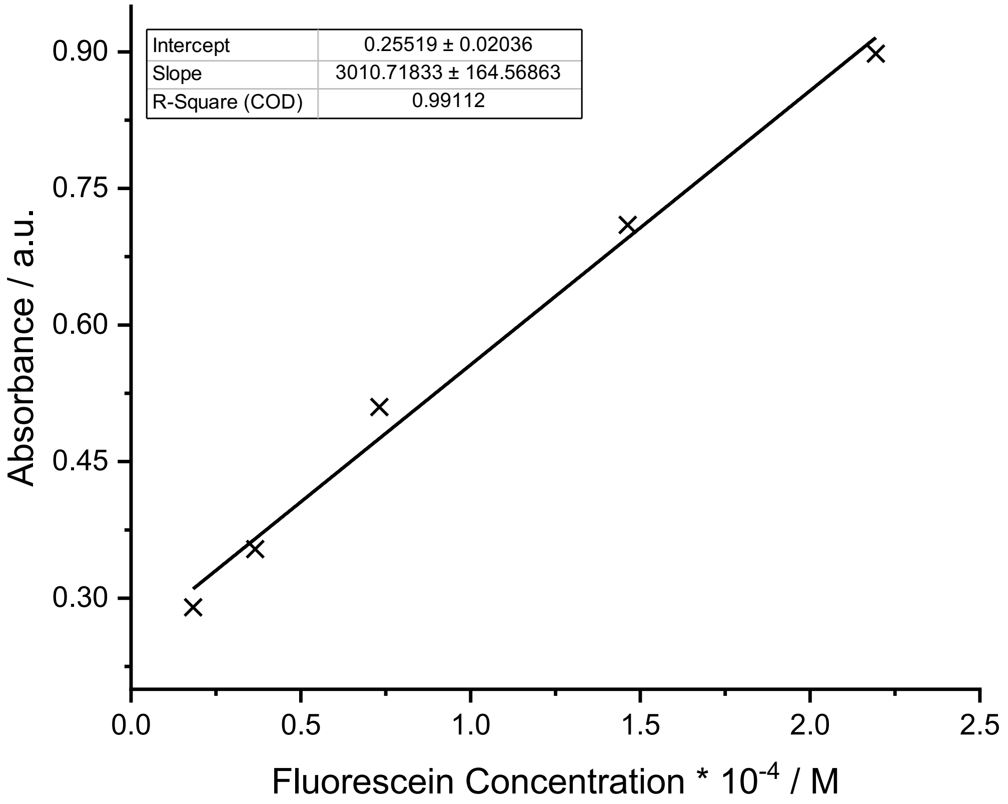

Figure S14. *UV-Vis calibration graph of Fluorescein-5-maleimide.*

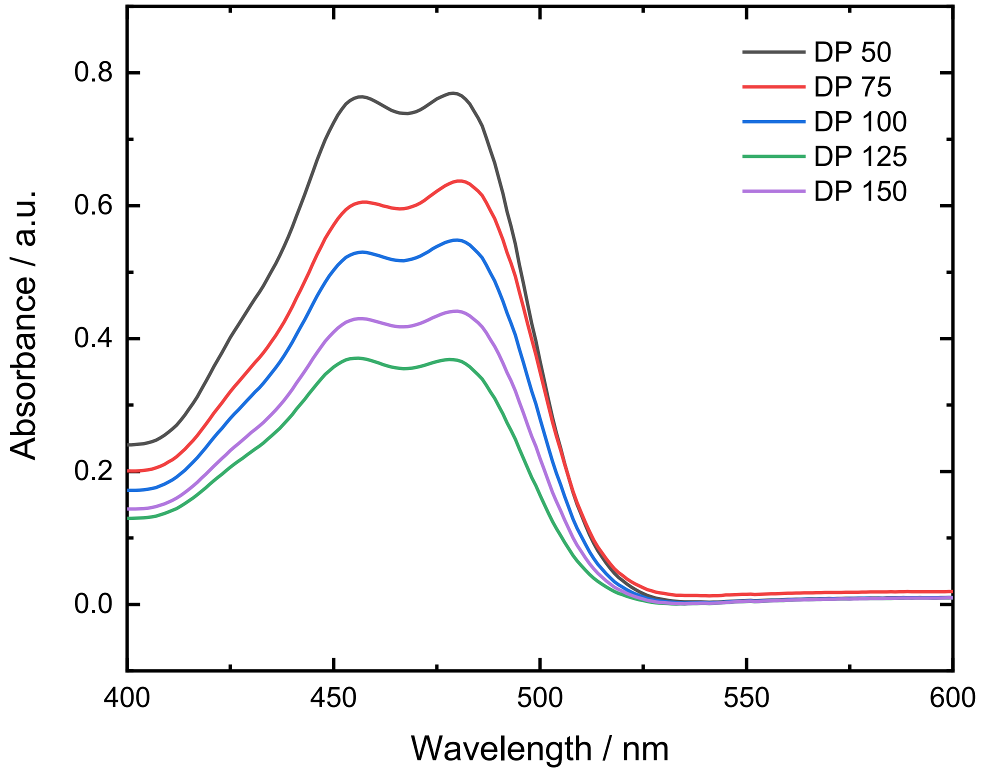

Figure S15. *UV-Vis spectrum of p(HEA)$_n$-DBCO-Fl polymers.*

2.2 Optimum $Ac_4ManNAz$ Concentration

2.2.1 DMSO Cytotoxicity

Table S4. *A549 cells treated with different concentrations of DMSO (0 - 1.50 %, 4 days) to determine average total cell viability relative to control cells using the resazurin reduction assay.*

DMSO Concentration / %	Cell Viability Relative to Control Cells / %
0	100.0 ± 4.1
0.1	97.4 ± 4.4
0.25	97.4 ± 11.5
0.4	98.4 ± 4.9
0.5	96.6 ± 2.4
0.75	92.1 ± 1.8
1.00	57.8 ± 2.0
1.25	44.0 ± 4.4
1.50	37.6 ± 4.9

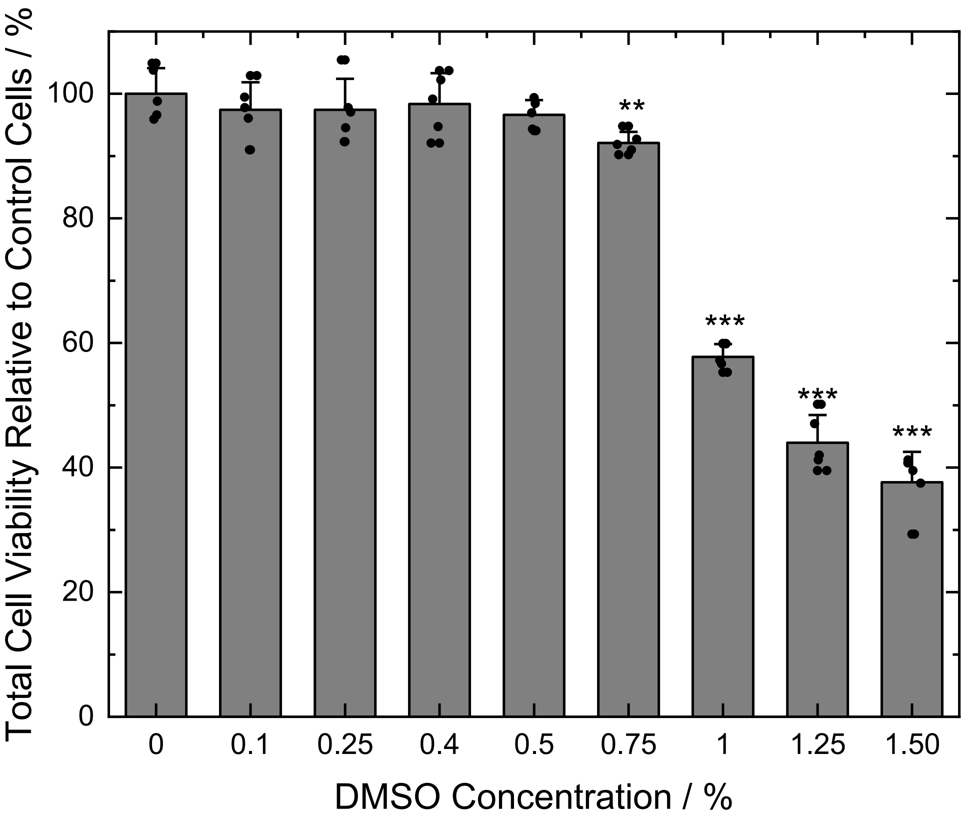

Figure S16. *Cytotoxicity of A549 cells incubated with DMSO at various concentration (0 – 1.5 %, 4 days) determined using Resazurin reduction assay. Cell viability has been documented relative to control cells. n = 5.*

2.2.2 Live cell imaging before flow cytometry

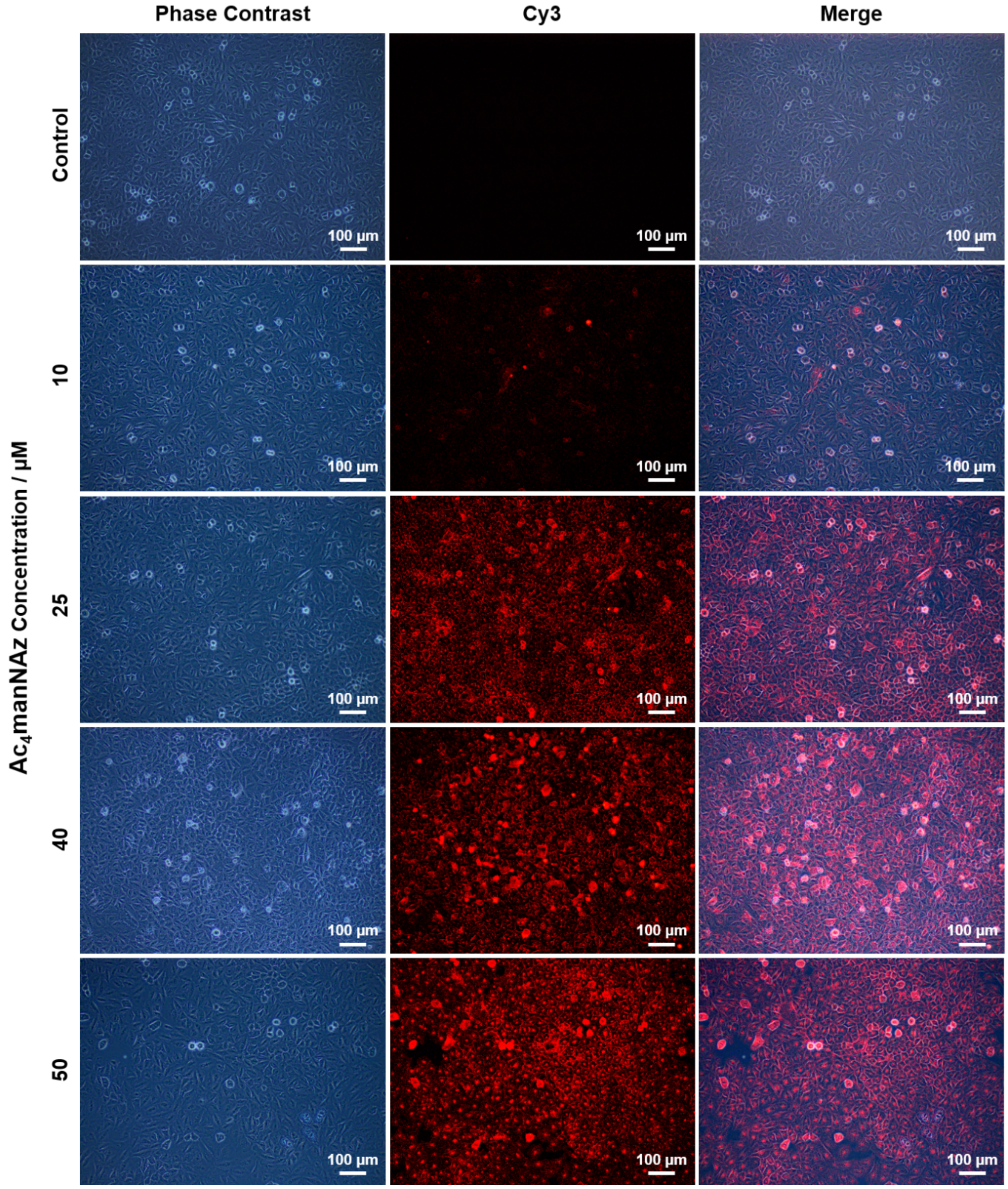

Figure S17. *Live cell imaging of A549 cells treated with varying concentrations of* $Ac_4ManNAz$ *(0-50 μM) for 96 h and DBCO-Cy3 (2.5 h, 50 μM) prior to flow cytometry. Scale bar = 100 μm.*

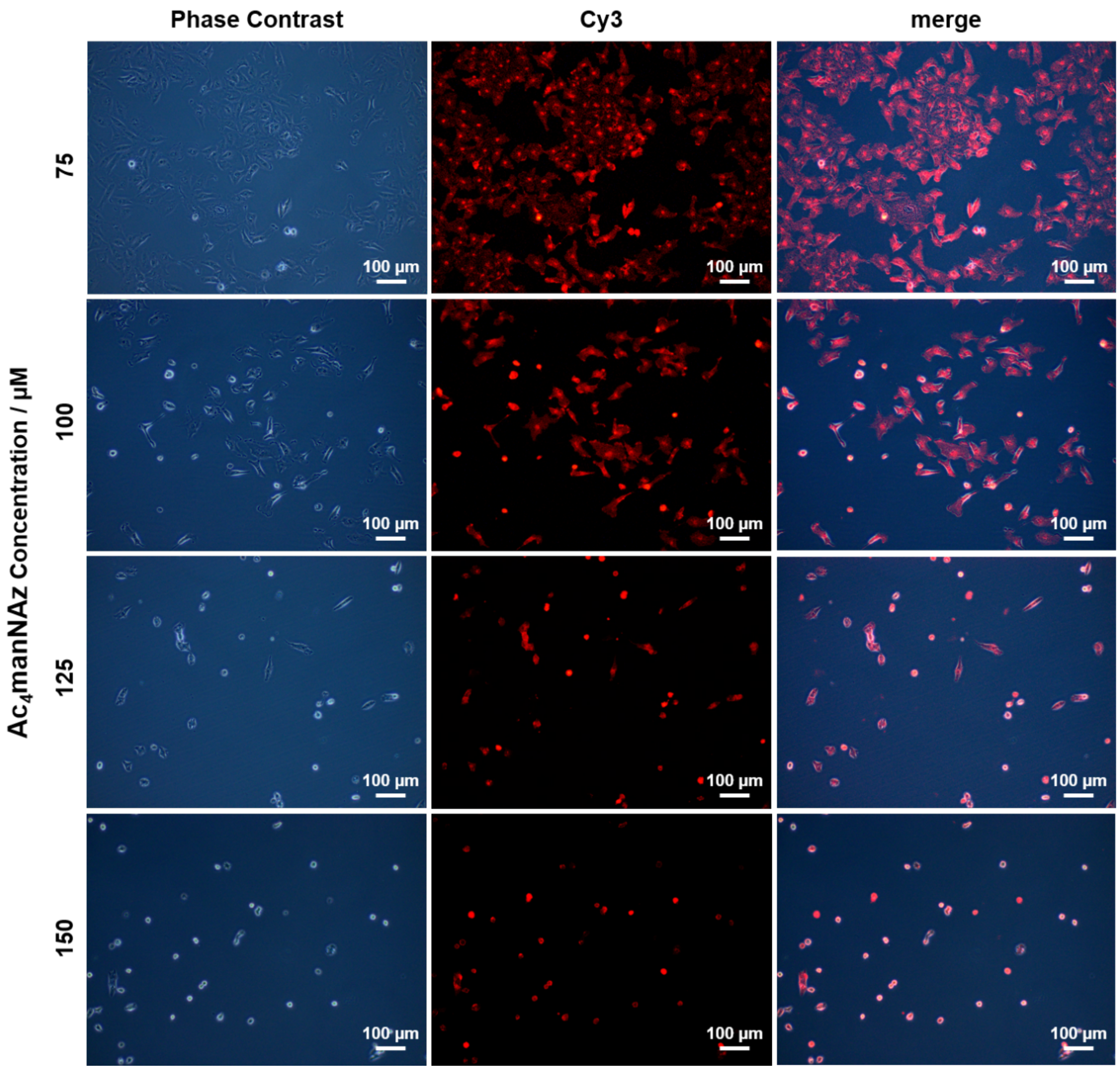

Figure S18. *Live cell imaging of A549 cells treated with varying concentrations of Ac*$_4$*ManNAz (75-150 μM) for 96 h and DBCO-Cy3 (2.5 h, 50 μM) prior to flow cytometry. Scale bar = 100 μm.*

2.2.3 Flow cytometry

Table S5*. Average fluorescence intensity measurements obtained from (live) A549 cells treated with different concentrations of Ac_4ManNAz (10-100 µM, 96 h) and DBCO-Cy3 (50 µM, 2.5) using flow cytometry.*

Ac_4ManNAz concentration / µM	Average Fluorescence Intensity / a.u.
10	19.7 ± 0.5
25	50.5 ± 4.9
40	105.2 ± 11.5
50	106.1 ± 8.5
75	233.7 ± 15.5
100	256 ± 67.7

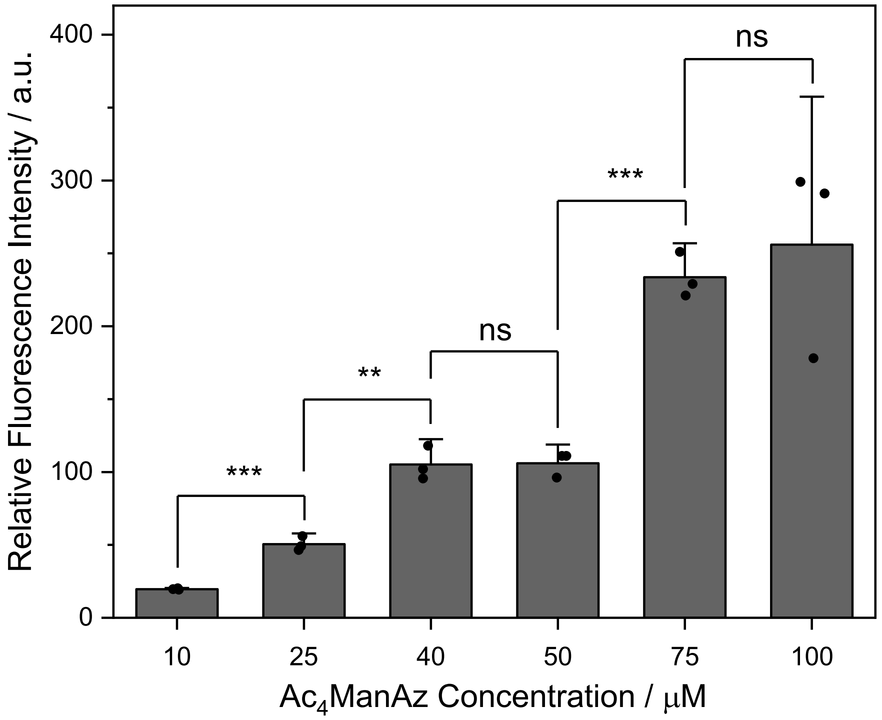

Figure S19. *Mean Cy3 fluorescence intensity measurements of (live) A549 cells treated with different concentrations of Ac_4ManNAz (96 h) and DBCO-Cy3 (50 µM, 2.5 h) determined by flow cytometry (n=3).*

2.2.4 Confocal Imaging

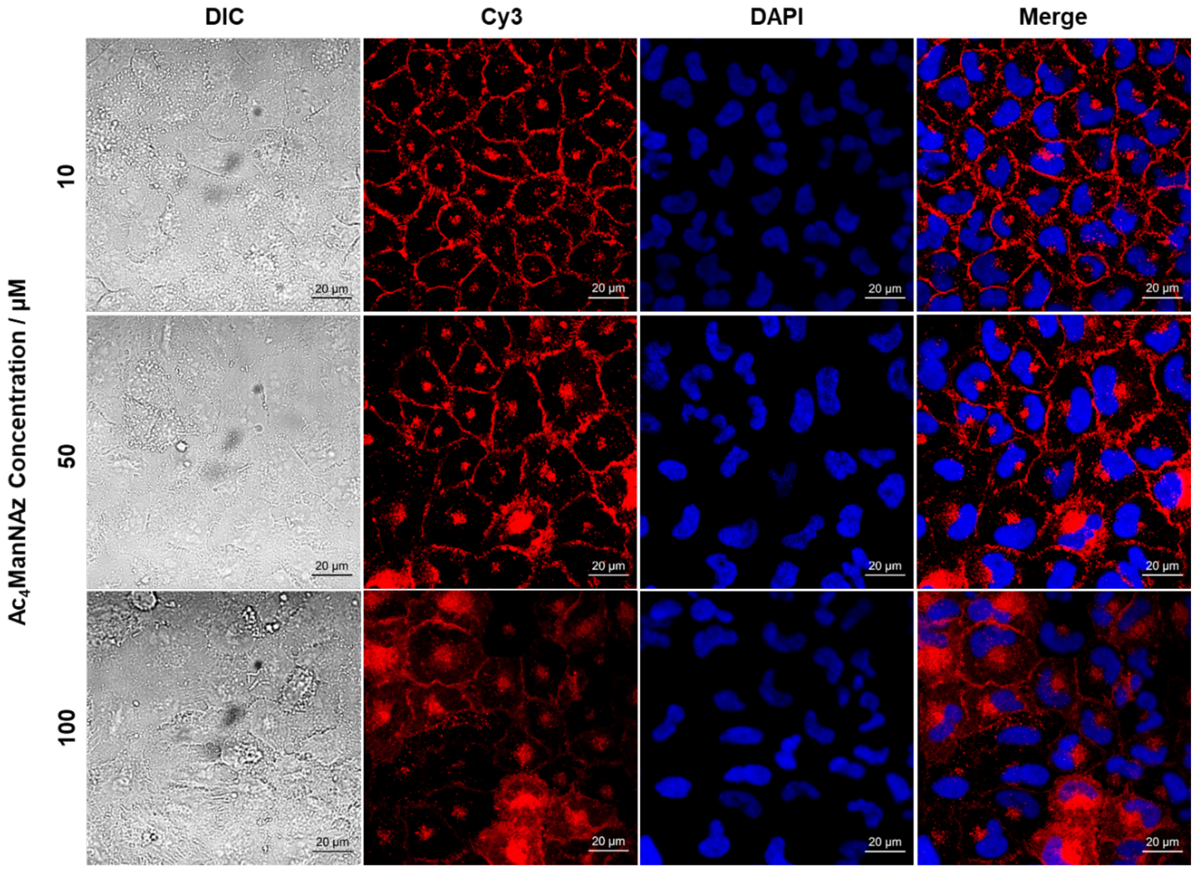

Figure S20. *Confocal images of fixed A549 cells treated with varying concentrations of $Ac_4ManNAz$ (96 h) and DBCO-Cy3 (2.5 h, 50 µM). Scale bar = 20 µm.*

2.3 Optimum pHEA$_n$DBCOFITC Concentration

2.3.1 Cytotoxicity

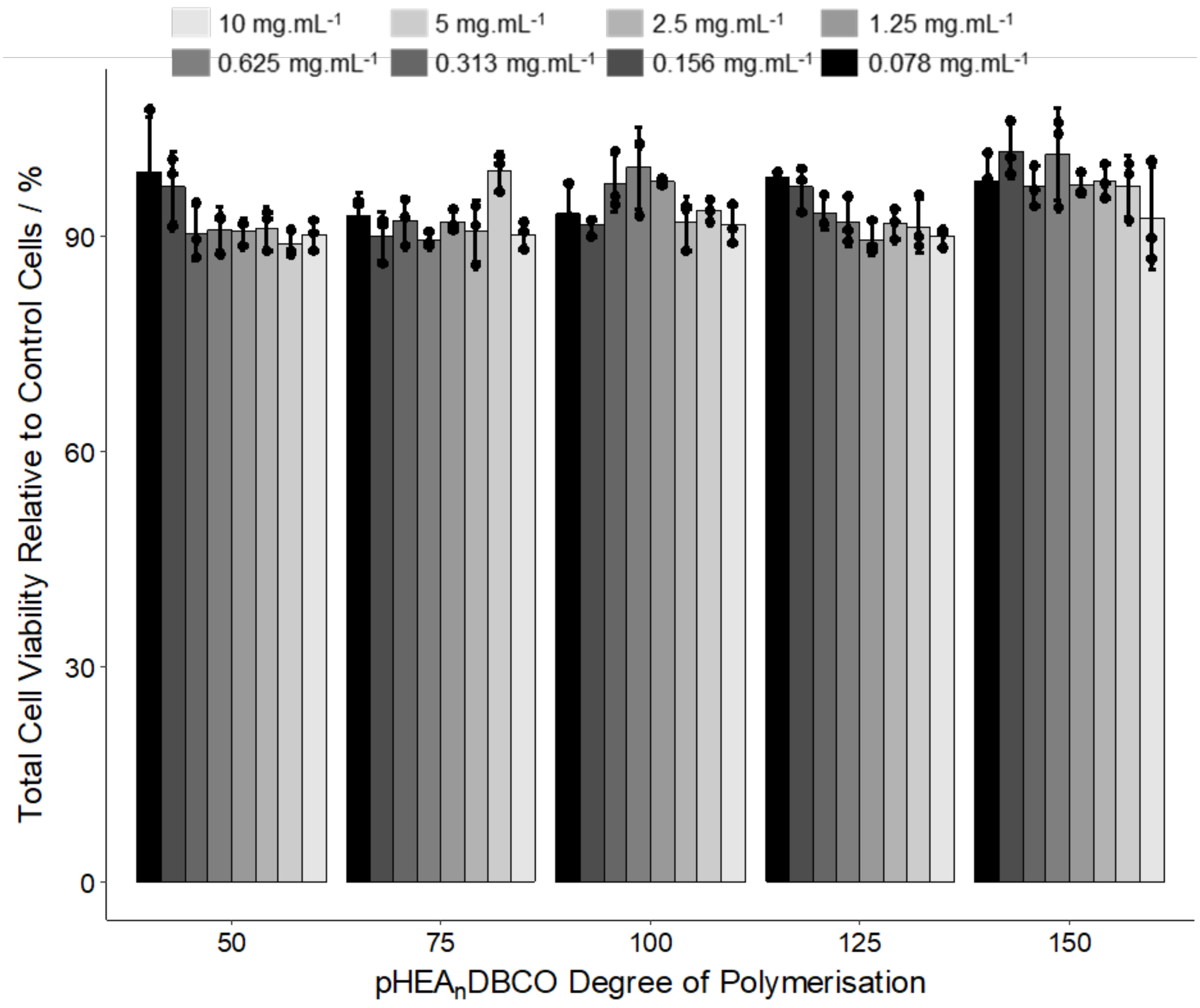

Figure S21. *Cell viability of A549 cells 24 hours following incubation with Ac$_4$ManNAz (40 µM, 96 h) and pHEA$_n$DBCO polymers (2.5 h) of different chain lengths, relative to untreated control A549 cells, determined using the Alamar Blue Resazurin assay. Polymer concentrations ranging from 0.156 mg.mL^{-1} to 10 mg.mL^{-1} were tested represented by increasing grayscale (n=3).*

Table S6. *Percentage cell viability of A549 cells incubated with $Ac_4ManNAz$ (40 µM, 96 h) and $pHEA_nDBCO$ polymers (2.5 h) of different chain lengths, relative to untreated control A549 cells, determined using the Alamar Blue Resazurin assay. Polymer concentrations ranged from 0.156 $mg.mL^{-1}$ to 10 $mg.mL^{-1}$ (n=3).*

Polymer	Concentration / $mg.mL^{-1}$							
	0.078	0.156	0.313	0.625	1.25	2.5	5	10
DBCO-$pHEA_{50}$	98.7 ± 7.7	96.8 ± 4.9	90.4 ± 3.8	90.8 ± 3.0	90.6 ± 1.8	91.1 ± 2.8	88.8 ± 1.8	90.1 ± 2.1
DBCO-$pHEA_{75}$	92.9 ± 3.1	89.9 ± 3.3	92.1 ± 3.2	89.4 ± 1.0	92.0 ± 1.5	90.5 ± 4.2	99.1 ± 2.6	90.2 ± 4.2
DBCO-$pHEA_{100}$	93.0 ± 4.2	91.4 ± 1.2	97.3 ± 4.0	99.4 ± 5.8	97.5 ± 3.8	92.0 ± 3.5	93.4 ± 1.6	91.5 ± 2.7
DBCO-$pHEA_{125}$	98.1 ± 0.7	96.8 ± 3.1	93.0 ± 2.3	91.8 ± 3.2	89.5 ± 2.4	91.6 ± 2.1	91.4 ± 3.8	89.8 ± 1.3
DBCO-$pHEA_{150}$	97.5 ± 4.3	102 ± 4	96.8 ± 2.8	101 ± 6	97.1 ± 1.6	97.5 ± 2.4	96.9 ± 4.2	92.3 ± 7.2

2.3.2 Flow Cytometry

Table S7. *Average fluorescence intensity of (live) A549 cells treated with $Ac_4ManNAz$ (40 µM, 96 h) and polymers of different chain lengths (2.5 h) using flow cytometry (n = 3).*

Polymer	Concentration / $mg.mL^{-1}$				
	20	10	5	2.5	1.25
DBCO-$pHEA_{50}$	3368 ± 511	3064 ± 131	2028 ± 122	998 ± 183	706 ± 60
DBCO-$pHEA_{75}$	1567 ± 76	1550 ± 19	798 ± 142	427 ± 84	277.± 34
DBCO-$pHEA_{100}$	1388 ± 67	1109 ± 23	517 ± 57	277 ± 38	164 ± 10
DBCO-$pHEA_{125}$	1066 ± 109	708 ± 60	332 ± 113	189 ± 69	107 ± 39
DBCO-$pHEA_{150}$	769 ± 125	588 ± 13	239 ± 95	129 ± 34	75 ± 23

2.3.3 Confocal Microscopy

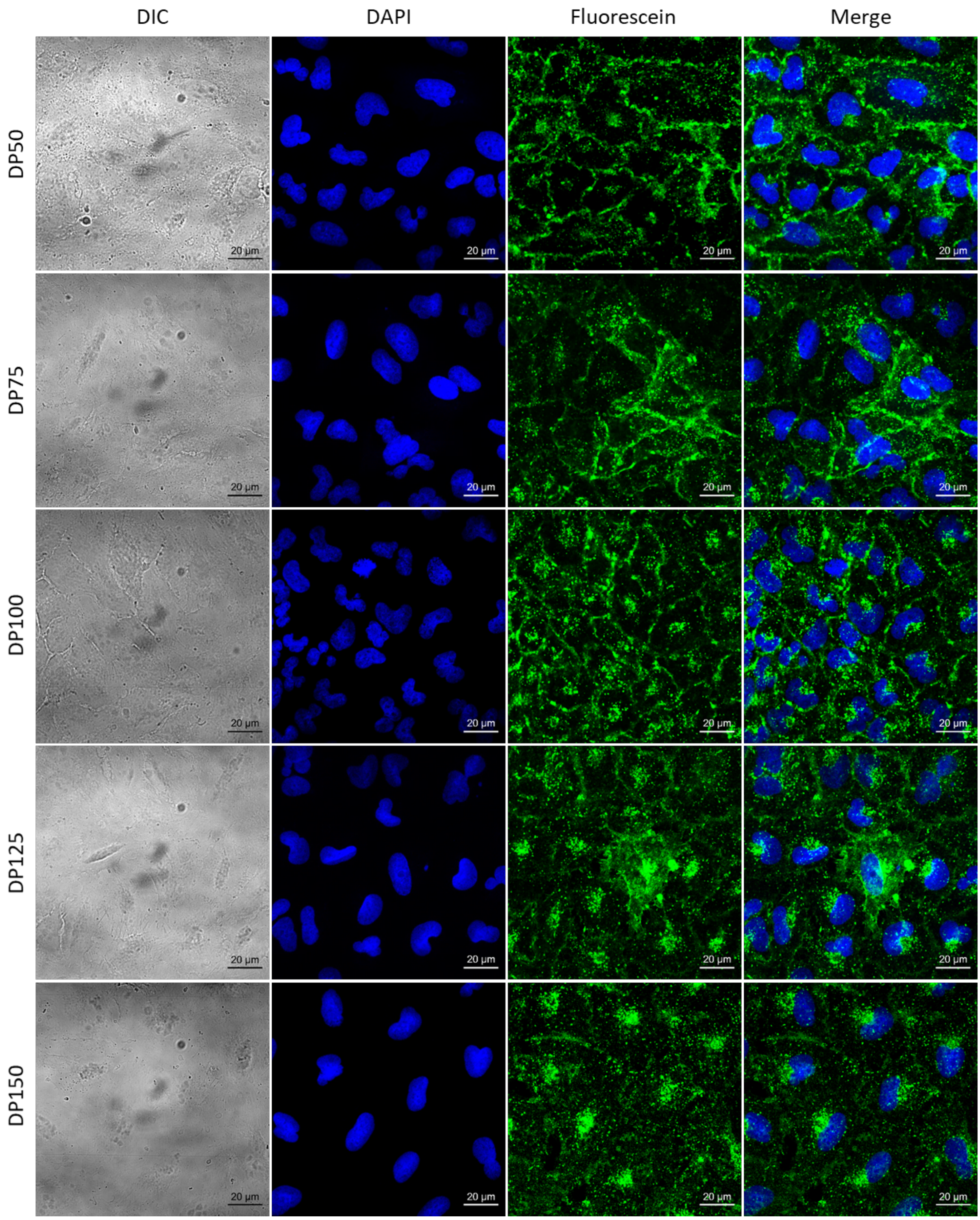

Figure S22. *Confocal imaging of (fixed) A549 cells treated with $Ac_4ManNAz$ (40 µM, 96 h) and $DBCOpHEA_nFl$ polymers (2.5 h, 10 mg.mL^{-1}) to visualize polymer coverage at the optimum concentration. Scale bar = 20 µm.*

2.4 Extent of Non-Specific Binding

2.4.1 Flow Cytometry

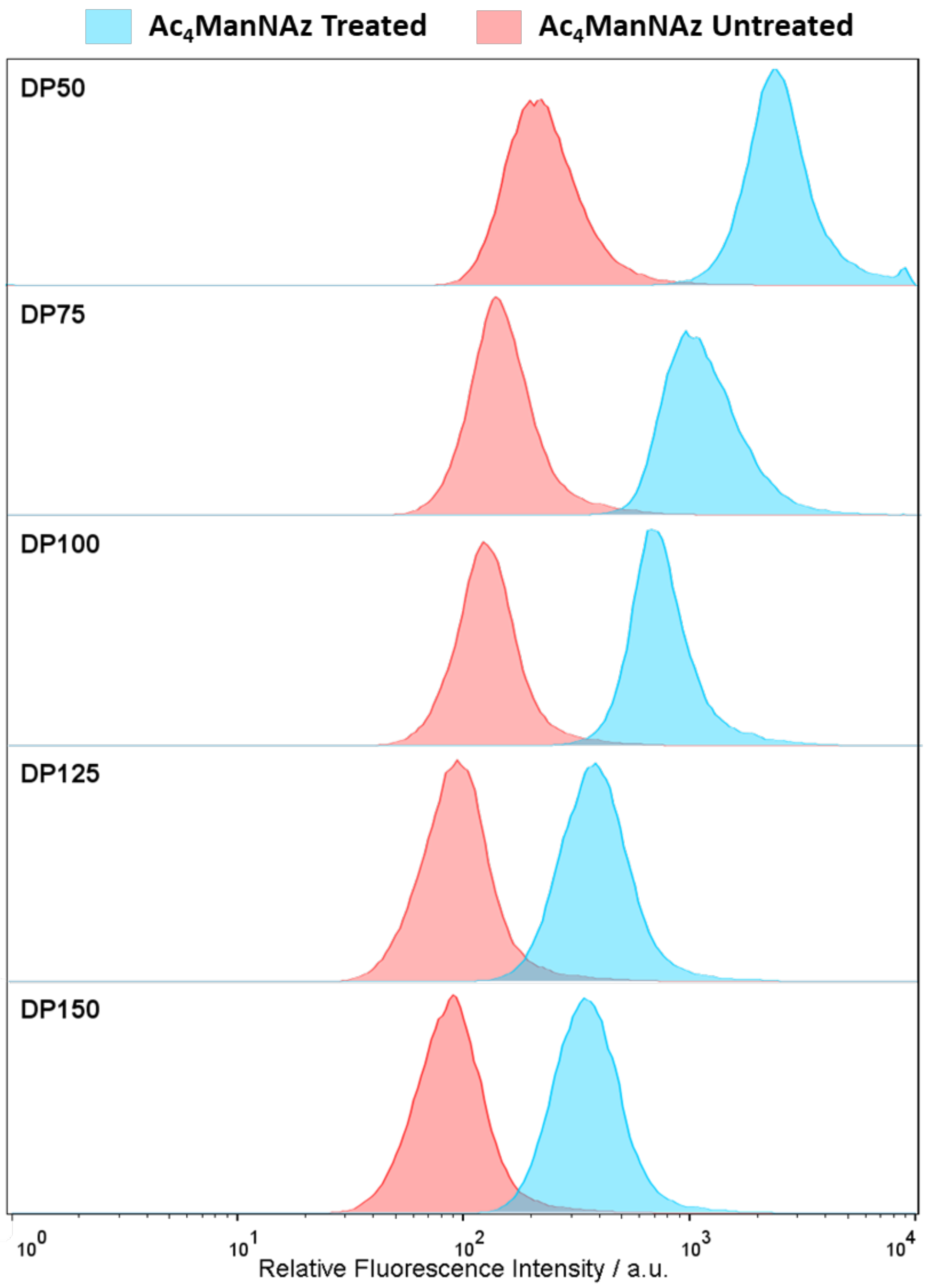

Figure S23. *Flow cytometry of (live) A549 cells untreated (red) or treated (blue) with $Ac_4ManNAz$ (40 µM, 96 h) and incubated with polymers of different chain lengths (5 mg.mL^{-1}, 2.5 h) to investigate the extent of non-specific binding at lower polymer concentrations.*

Table S8. *Average fluorescence intensity of (live) A549 cells untreated and treated with Ac$_4$ManNAz (40 µM, 96 h) and polymers of different chain lengths (2.5 h) using flow cytometry. (n = 5).*

	Concentration / mg.mL^{-1}			
	5		10	
Polymer	Untreated	Untreated	Untreated	treated
DBCO-pHEA$_{50}$	239 ± 13	2178 ± 61	283 ± 58	2996 ± 221
DBCO-pHEA$_{75}$	152 ± 7	1020 ± 65	179 ± 38	1531 ± 177
DBCO-pHEA$_{100}$	132 ± 4.5	630 ± 105	136 ± 27	1057 ± 79
DBCO-pHEA$_{125}$	84 ± 19	373 ± 13	101 ± 29	654 ± 87
DBCO-pHEA$_{150}$	79 ± 23	363 ± 12	104 ± 23	638 ± 45

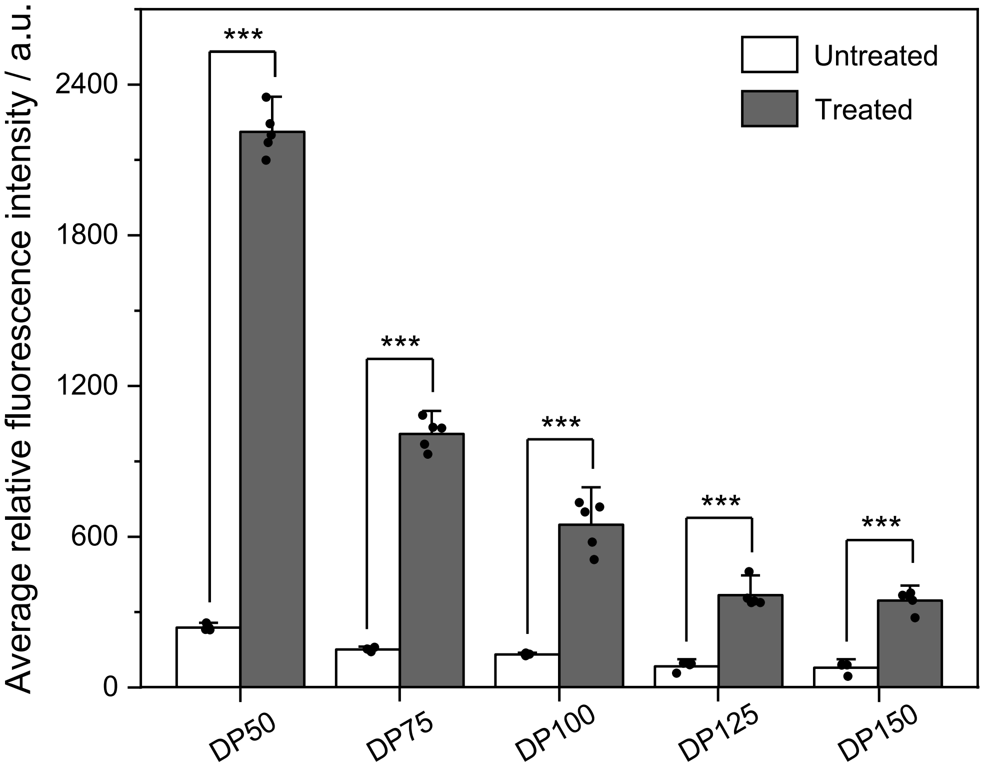

Figure S24. *Average fluorescence intensity of (live) A549 cells untreated and treated with Ac$_4$ManNAz (40 µM, 96 h) and polymers of different chain lengths (5 mg.mL^{-1}, 2.5 h) using flow cytometry (n = 5).*

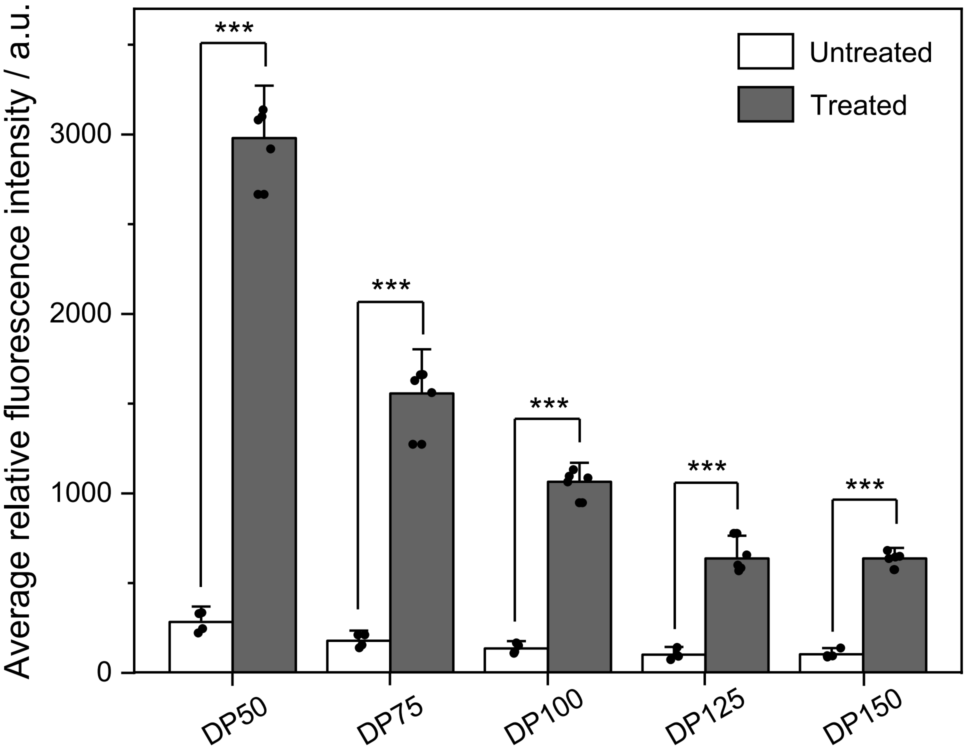

Figure S25. *Average fluorescence intensity of (live) A549 cells untreated and treated with Ac$_4$ManNAz (40 µM, 96 h) and polymers of different chain lengths (10 mg.mL^{-1}, 2.5 h) using flow cytometry (n = 5).*

Table S9. *Percentage of cells treated with both $Ac_4ManNAz$ (40 µM, 96 h) and polymers of different chain lengths (2.5 h) with fluorescence intensity above that of 99% of cells untreated with both $Ac_4ManNAz$ and $DBCOpHEA_nFl$ and 95% or 99% of cells untreated with $Ac_4ManNAz$ but treated with $DBCOpHEA_nFl$ (non-specific binding threshold) (n = 5).*

	Polymer concentration / $mg.mL^{-1}$					
	5			10		
Polymer	Control	95%	99%	Control	95%	99%
DBCO-$pHEA_{50}$	99.5 ± 0.2	99.2 ± 0.2	98.4 ± 0.2	99.6 ± 0.3	98.9 ± 0.5	98.3 ± 0.8
DBCO-$pHEA_{75}$	99.3 ± 0.5	98.7 ± 0.8	95.8 ± 0.8	99.5 ± 0.3	98.9 ± 0.4	98.4 ± 0.4
DBCO-$pHEA_{100}$	99.6 ± 0.4	98.1 ± 2.7	79.5 ± 16.1	99.2 ± 0.5	98.7 ± 0.7	97.9 ± 0.7
DBCO-$pHEA_{125}$	99.4 ± 0.2	96.2 ± 5.1	47.1 ± 10	99.4 ± 0.3	98.9 ± 0.2	97.2 ± 0.5
DBCO-$pHEA_{150}$	99.7 ± 0.1	99.1 ± 0.2	49.7 ± 18.5	99.5 ± 0.2	98.9 ± 0.5	95.6 ± 1.9

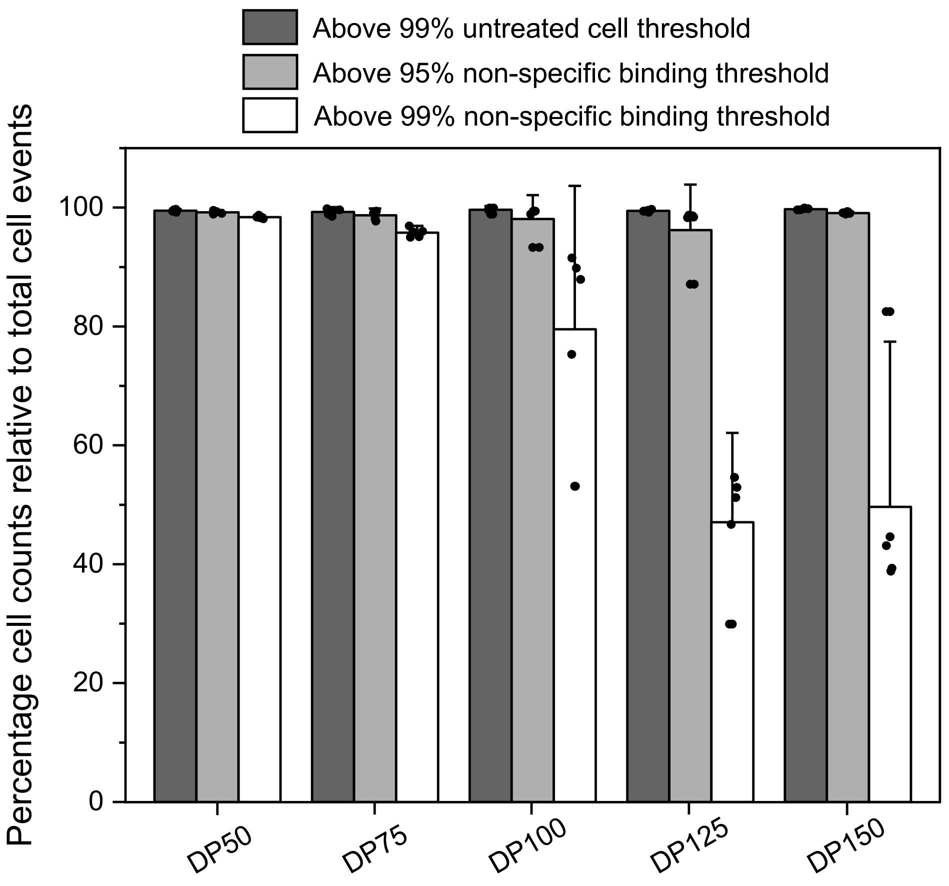

Figure S26. *Percentage of cells treated with both* $Ac_4ManNAz$ *(40 µM, 96 h) and polymers of different chain lengths (5 mg.ml*$^{-1}$*, 2.5 h) with fluorescence intensity above that of 99% of cells untreated with both* $Ac_4ManNAz$ *and* $DBCOpHEA_nFl$ *and 95% or 99% of cells untreated with* $Ac_4ManNAz$ *(non-specific binding threshold) (n = 5).*

2.4.2 Live Cell imaging before Flow Cytometry

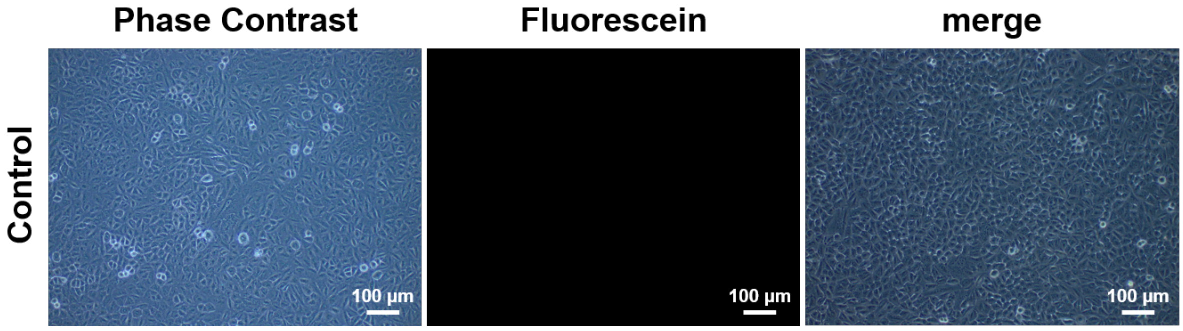

Figure S27. *A549 cells untreated with both Ac_4ManNAz and DBCOpHEA$_n$Fl polymers to visualize background fluorescence. Scale bar = 100 µm.*

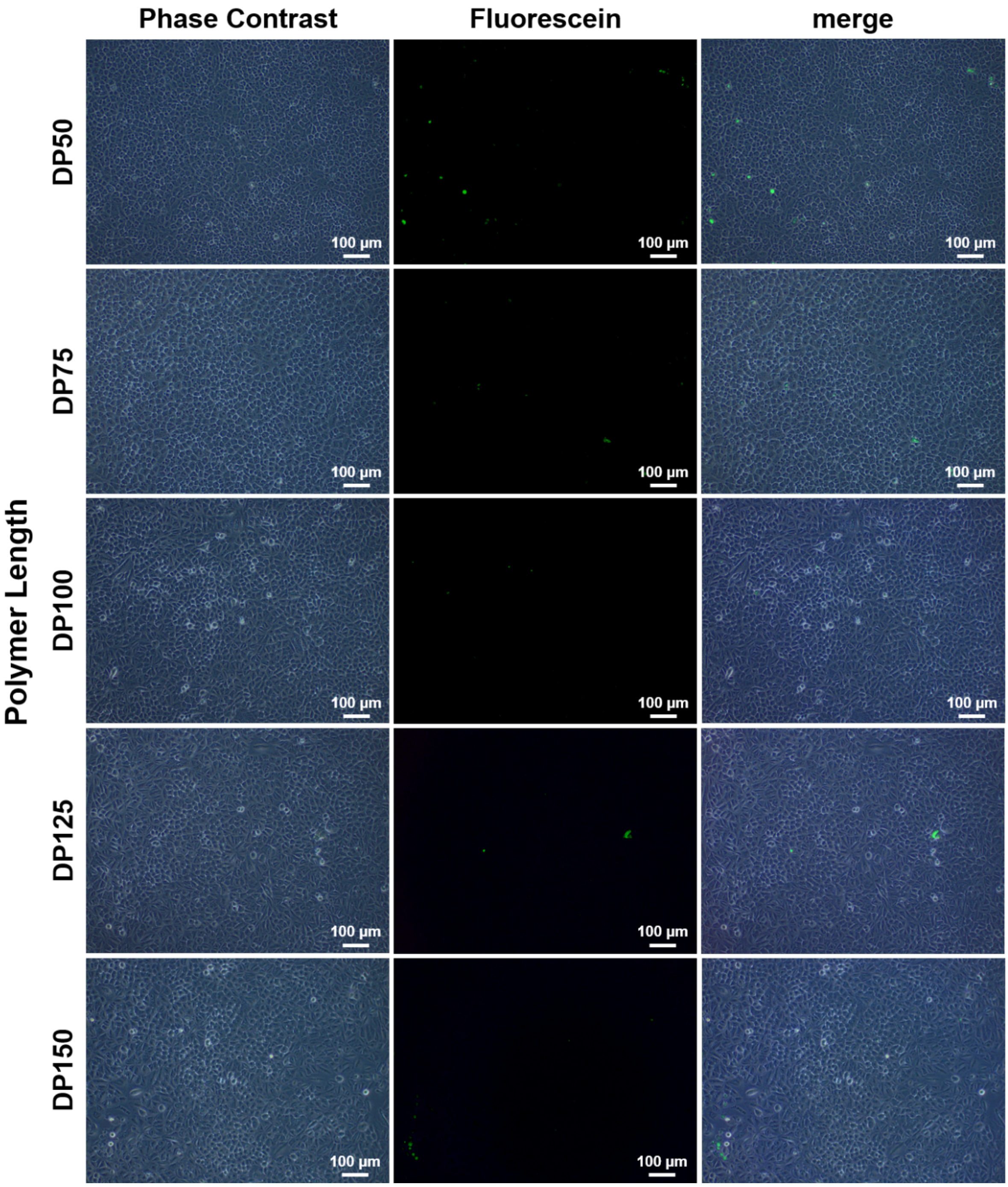

Figure S28. *Imaging of (live) A549 cells untreated with $Ac_4ManNAz$ but treated with $DBCOpHEA_nFl$ polymers varying in chain lengths (2.5 h, 10 mg.mL^{-1}) to determine the extent of non-specific binding. Images were taken immediately before flow cytometry. Scale bar = 100 µm.*

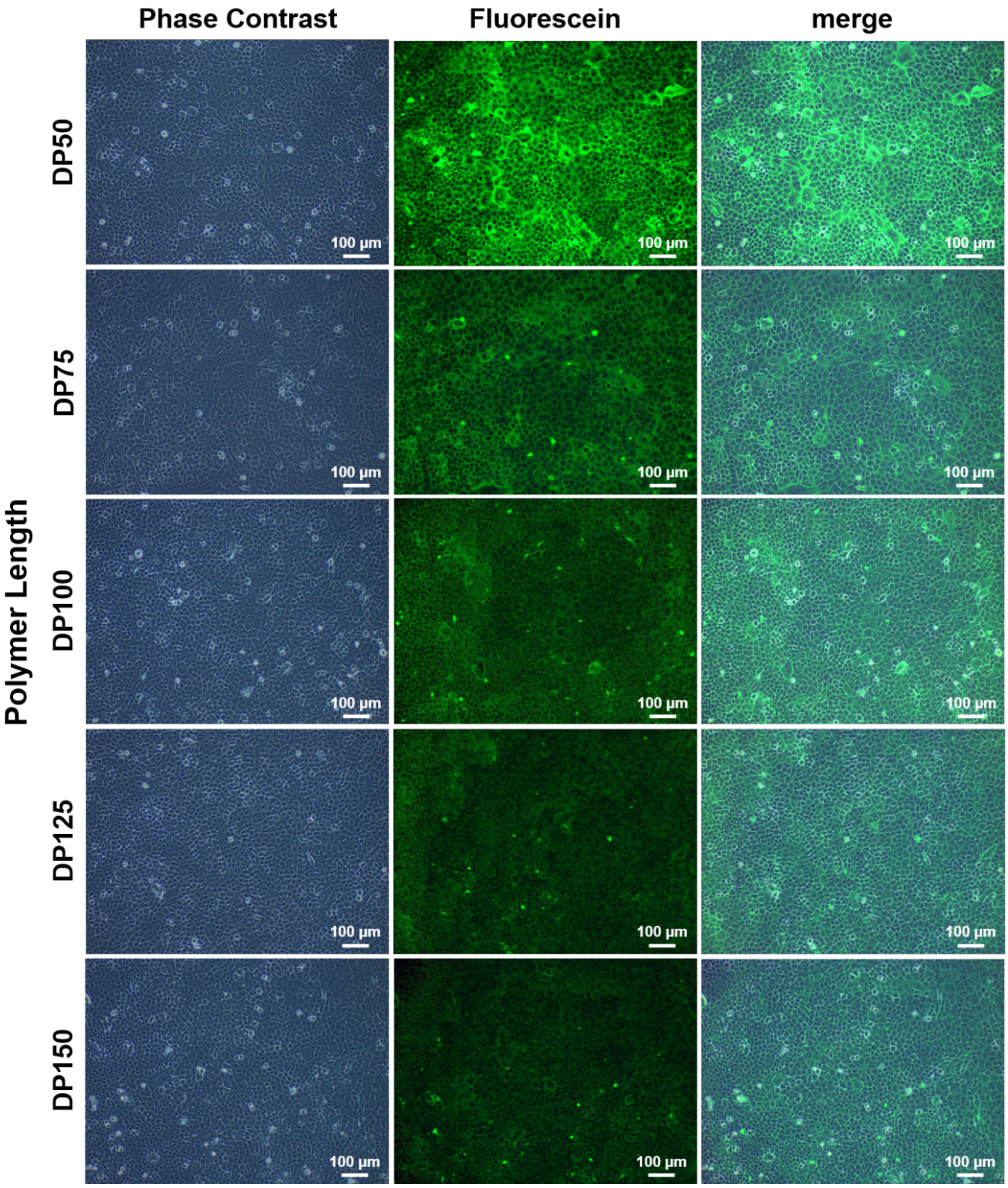

Figure S29. *Imaging of (live) A549 cells immediately following treatment with* $Ac_4ManNAz$ *(40 µM, 96 h) and* $DBCOpHEA_nFl$ *polymers varying in chain lengths (2.5 h, 10 mg.mL*$^{-1}$*) for comparison with control cells (Fig. 30) to determine the extent of nonspecific binding. Scale bar = 100 µm.*

2.4.3 Confocal Microscopy

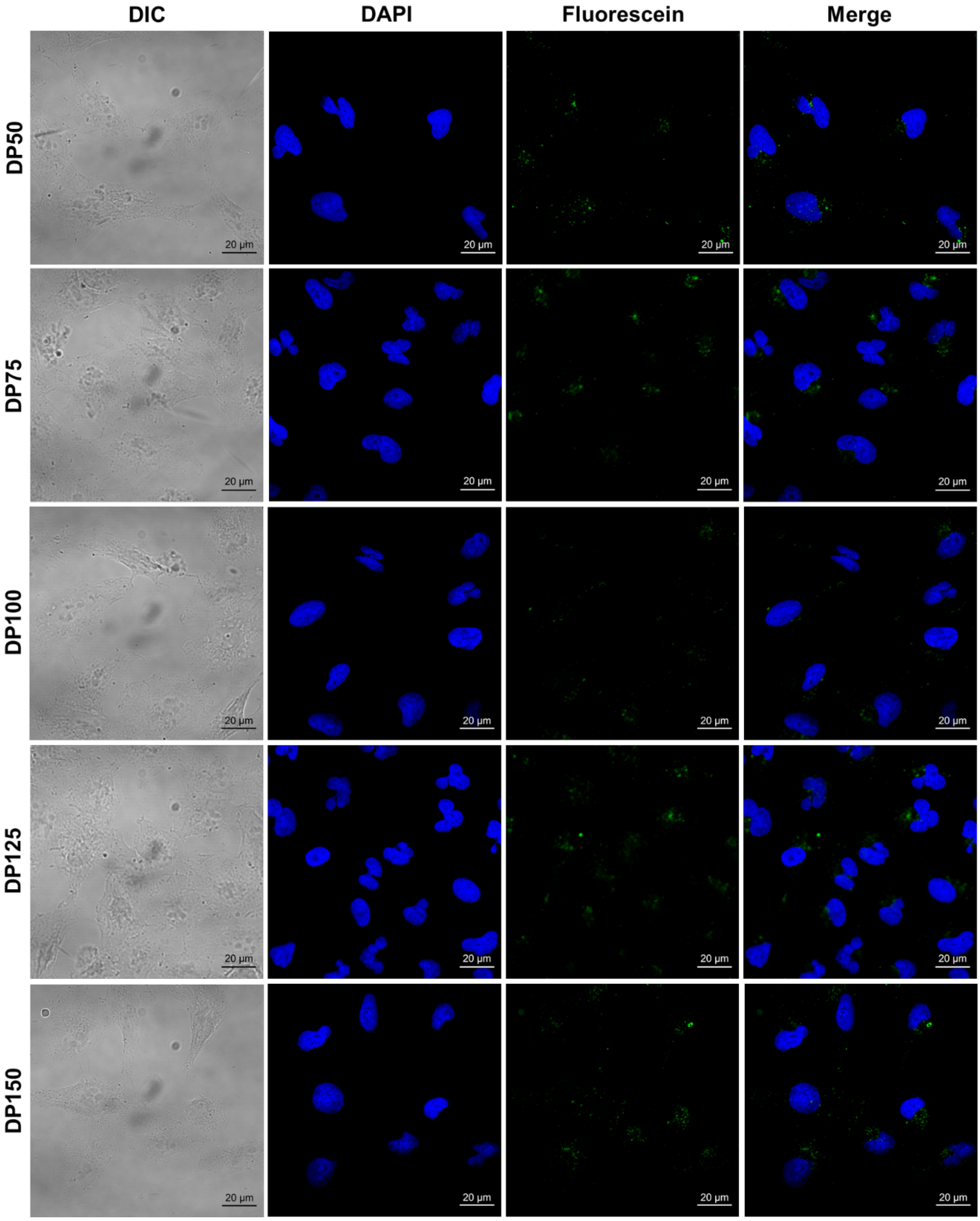

***Figure S30**. Confocal imaging of control (fixed) A549 cells untreated with $Ac_4ManNAz$ but treated with $DBCOpHEA_nFl$ polymers (10 mg.mL^{-1}, 2.5 h) to visualize non-specific binding. Scale bar = 20 µm.*

2.5 Cell Surface Degrafting of pHEA$_n$-DBCO-Fl

2.5.1 Flow Cytometry

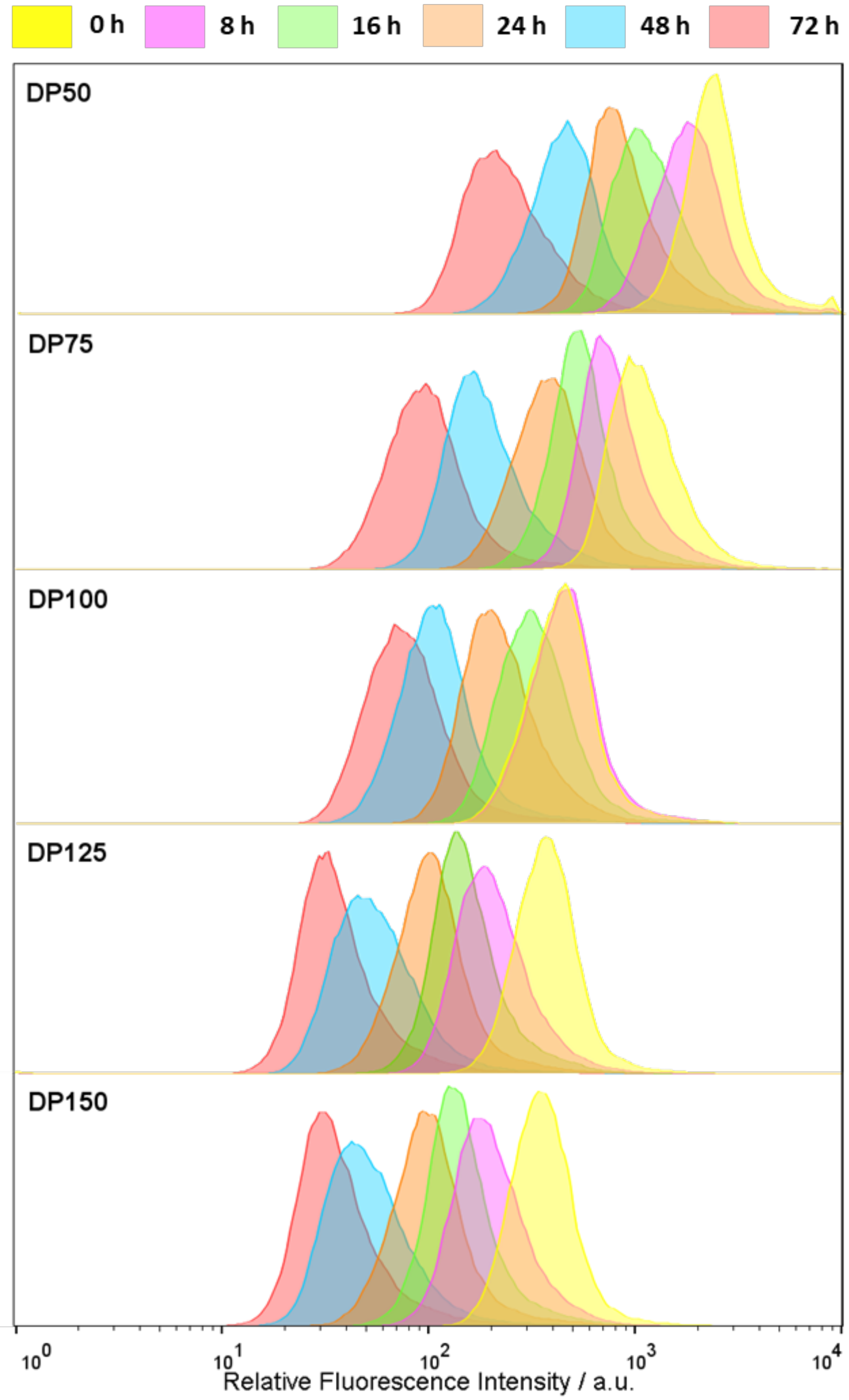

Figure S31. *Flow cytometry of (live) A549 cells treated with Ac$_4$ManNAz (40 µM, 96 h) and DBCOpHEA$_n$Fl polymers (5 mg.mL^{-1}, 2.5 h) over time.*

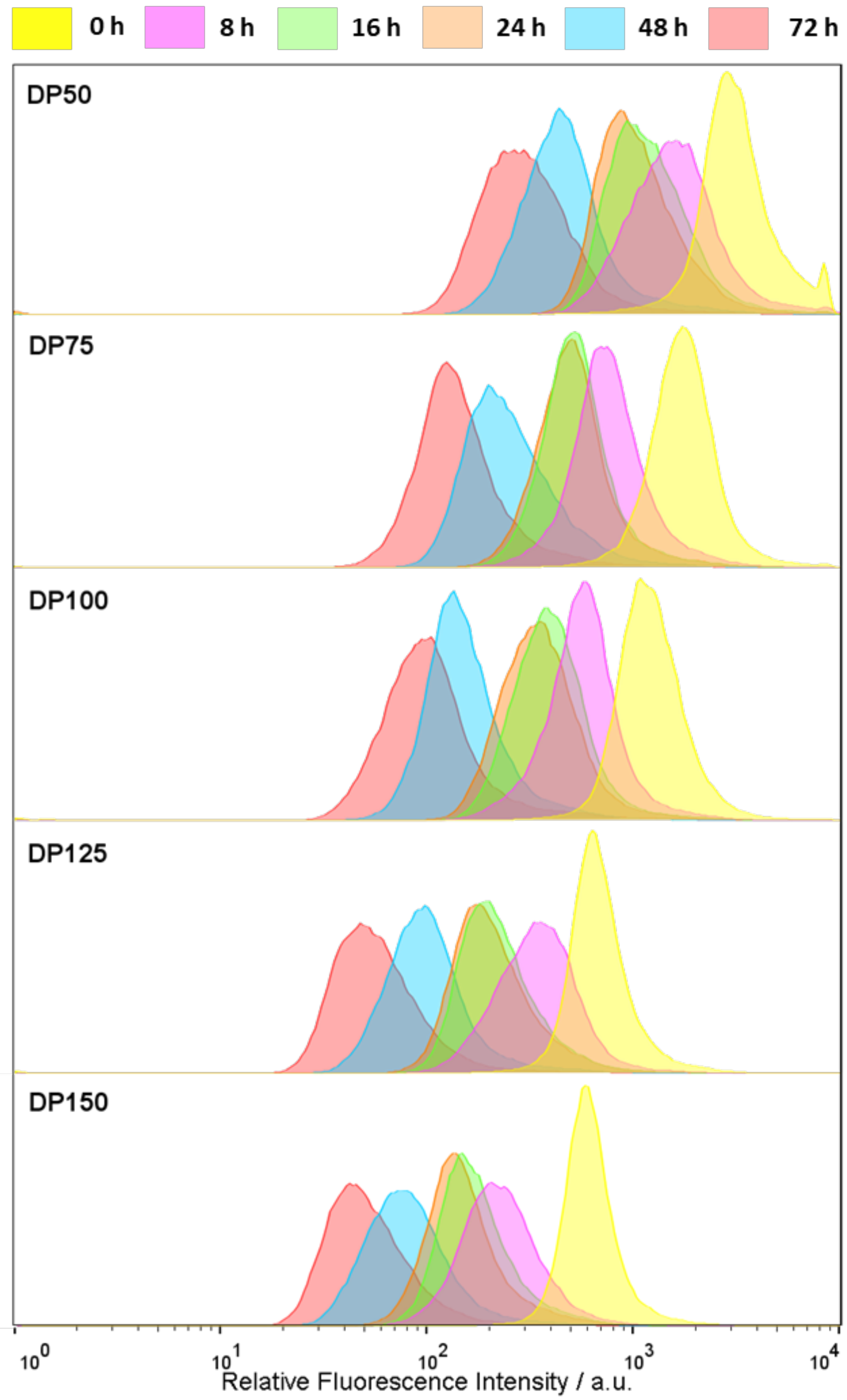

Figure S32. *Flow cytometry of (live) A549 cells treated with* $Ac_4ManNAz$ *(40 µM, 96 h) and* $DBCOpHEA_nFl$ *polymers (10 mg.mL*$^{-1}$*, 2.5 h) over time.*

Table S10. *Average fluorescence intensity measurements of A549 cells treated with $Ac_4ManNAz$ (40 µM, 96 h) and DBCOpHEA$_n$Fl polymers (5 mg.mL^{-1}, 2.5 h) post polymer conjugation. (n = 4).*

Polymer	Time post polymer conjugation / h					
	0	8	16	24	48	72
DBCO-pHEA$_{50}$	2212 ± 93	1768 ± 88	1291 ± 189	889 ± 53	475 ± 30	227 ± 47
DBCO-pHEA$_{75}$	1009 ± 61	747 ± 29	560 ± 76	383 ± 10	187 ± 3	107 ± 11
DBCO-pHEA$_{100}$	648 ± 99	468 ± 61	365 ± 117	231 ± 35	125 ± 26	85 ± 22
DBCO-pHEA$_{125}$	367 ± 40	240 ± 58	177 ± 36	114 ± 23	60 ± 9	42 ± 13
DBCO-pHEA$_{150}$	346 ± 52	234 ± 96	186 ± 81	115 ± 29	58 ± 20	42 ± 21

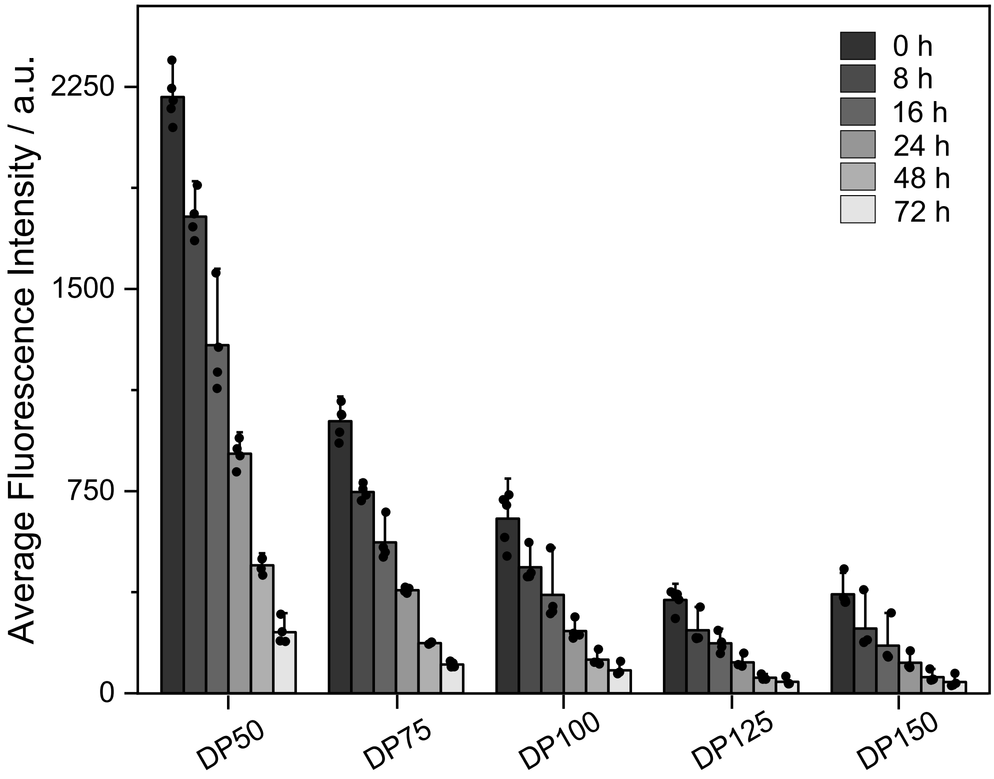

Figure S33. *Average fluorescence intensity measurements decrease of A549 cells treated with $Ac_4ManNAz$ (40 µM, 96 h) and pHEA$_n$DBCO polymers (5 mg.mL^{-1}, 2.5 h) over time (n = 4).*

Table S11. *Average fluorescence intensity measurements of A549 cells treated with Ac$_4$ManNAz (40 µM, 96 h) and DBCOpHEA$_n$Fl polymers (10 mg.mL^{-1}, 2.5 h) post polymer conjugation (n = 4).*

Polymer	Time post polymer conjugation / h					
	0	8	16	24	48	72
DBCO-pHEA$_{50}$	2980 ± 194	1695 ± 221	1365 ± 373	1191 ± 389	478 ± 69	354 ± 96
DBCO-pHEA$_{75}$	1557 ± 163	815 ± 81	613 ± 133	603 ± 229	244 ± 30	150 ± 20
DBCO-pHEA$_{100}$	1065 ± 70	607 ± 138	450 ± 194	359 ± 49	172 ± 31	127 ± 47
DBCO-pHEA$_{125}$	638 ± 39	340 ± 61	247 ± 94	205 ± 19	99 ± 13	73 ± 18
DBCO-pHEA$_{150}$	637 ± 85	316 ± 139	183 ± 43	149 ± 15	74 ± 16	64 ± 35

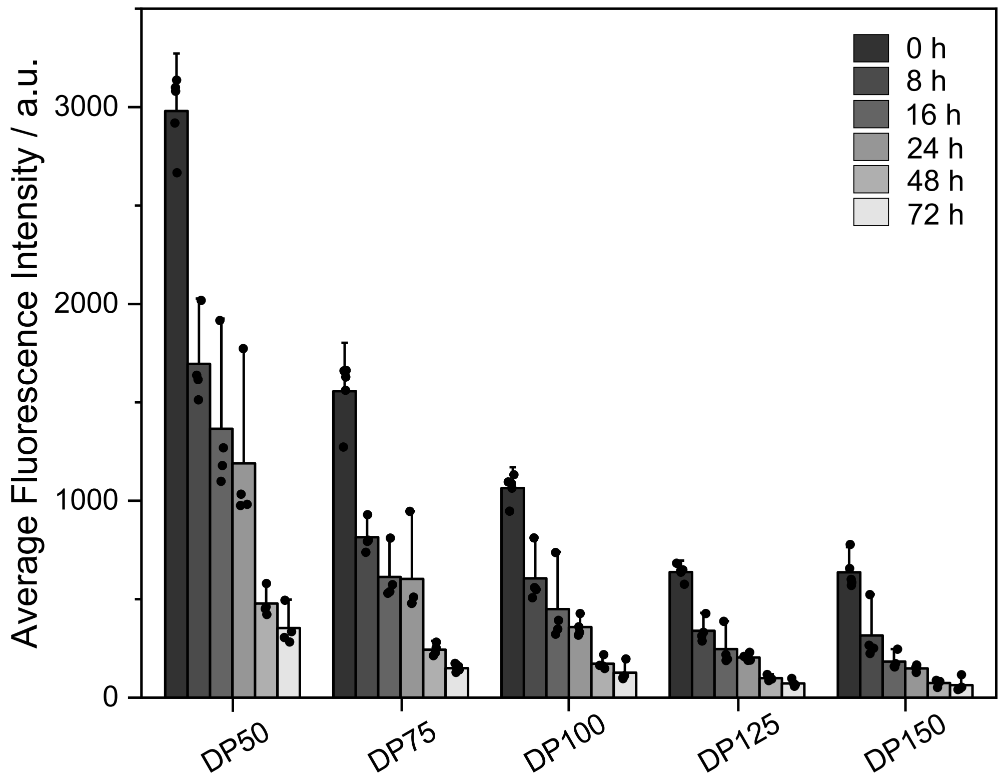

Figure S34. *Average fluorescence intensity measurements decrease of A549 cells treated with Ac$_4$ManNAz (40 µM, 96 h) and pHEA$_n$DBCO polymers (10 mg.mL^{-1}, 2.5 h) over time (n = 4).*

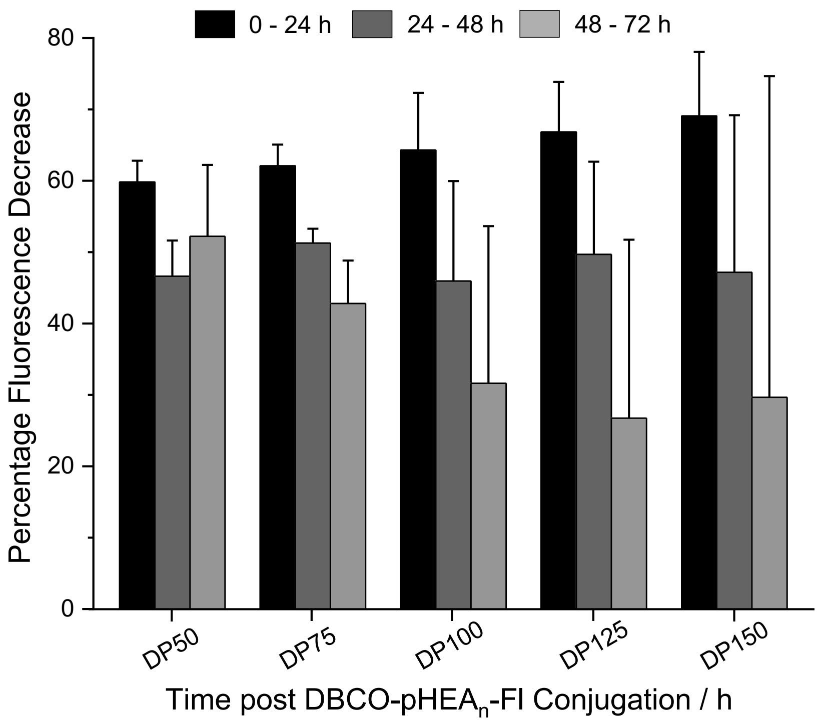

Figure S35. *Percentage decrease in fluorescence of A549 cells treated with $Ac_4ManNAz$ (40 µM, 96 h) and polymers of different chain lengths (5 mg.mL^{-1}, 2.5 h) measured over 72 h using flow cytometry (n = 4).*

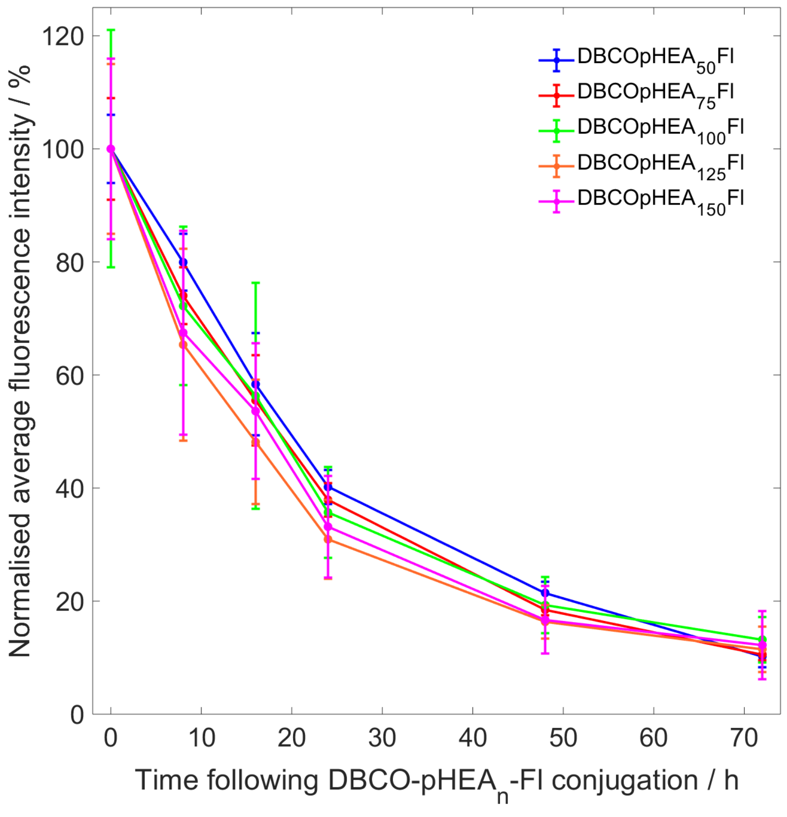

Figure S36. *Normalized average fluorescence intensity of A549 cells treated with $Ac_4ManNAz$ (40 µM, 96 h) and pHEA$_n$DBCO polymers (5 mg.mL^{-1}, 2.5 h) over time (n = 4).*

2.5.2 Confocal Microscopy

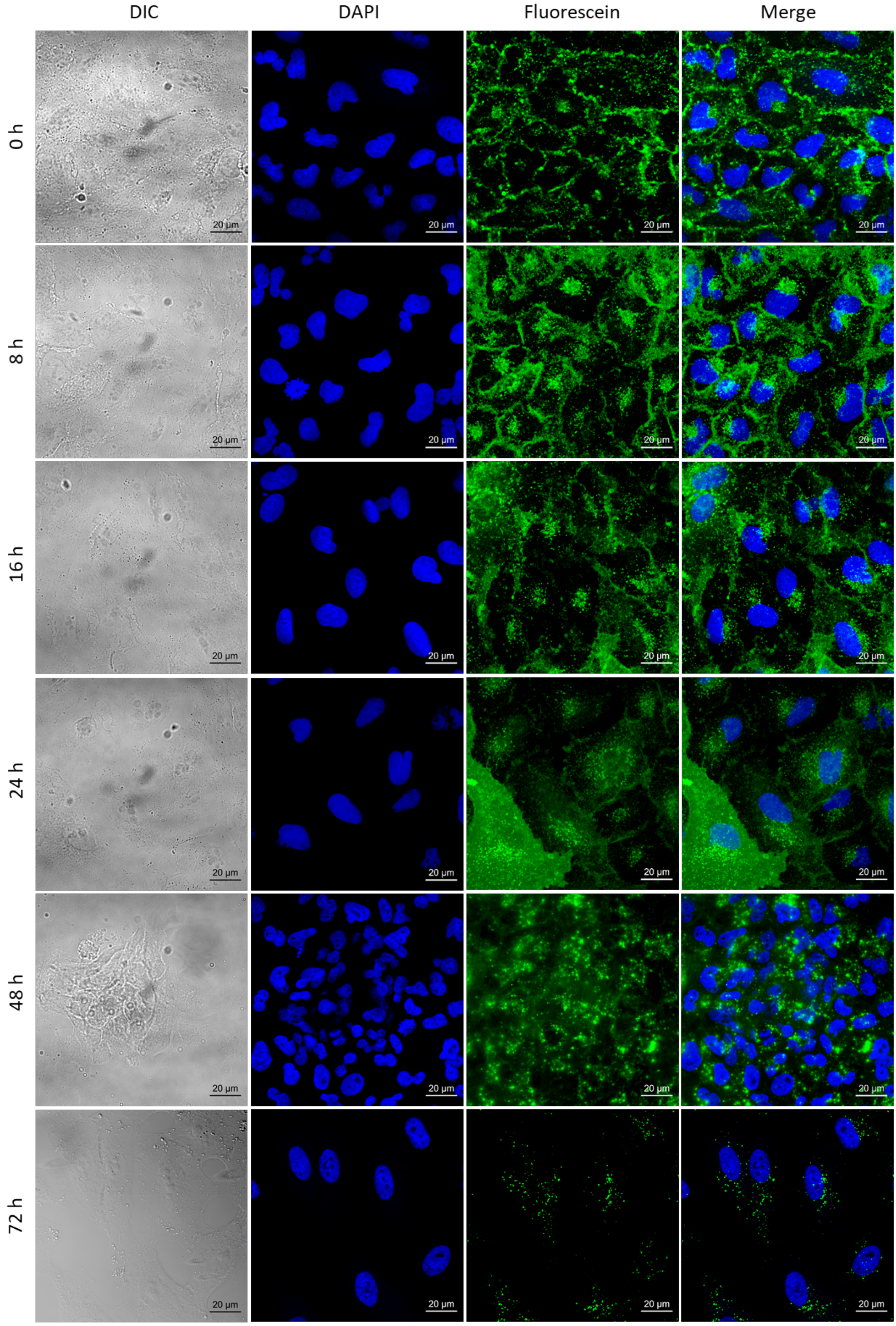

Figure S37. *Confocal imaging of control (fixed) A549 cells treated with $Ac_4ManNAz$ (40 µM, 96 h) and DBCOpHEA$_{50}$Fl polymers (2.5 h, 10 mg.mL^{-1}) over time. Scale bar = 20 µm.*

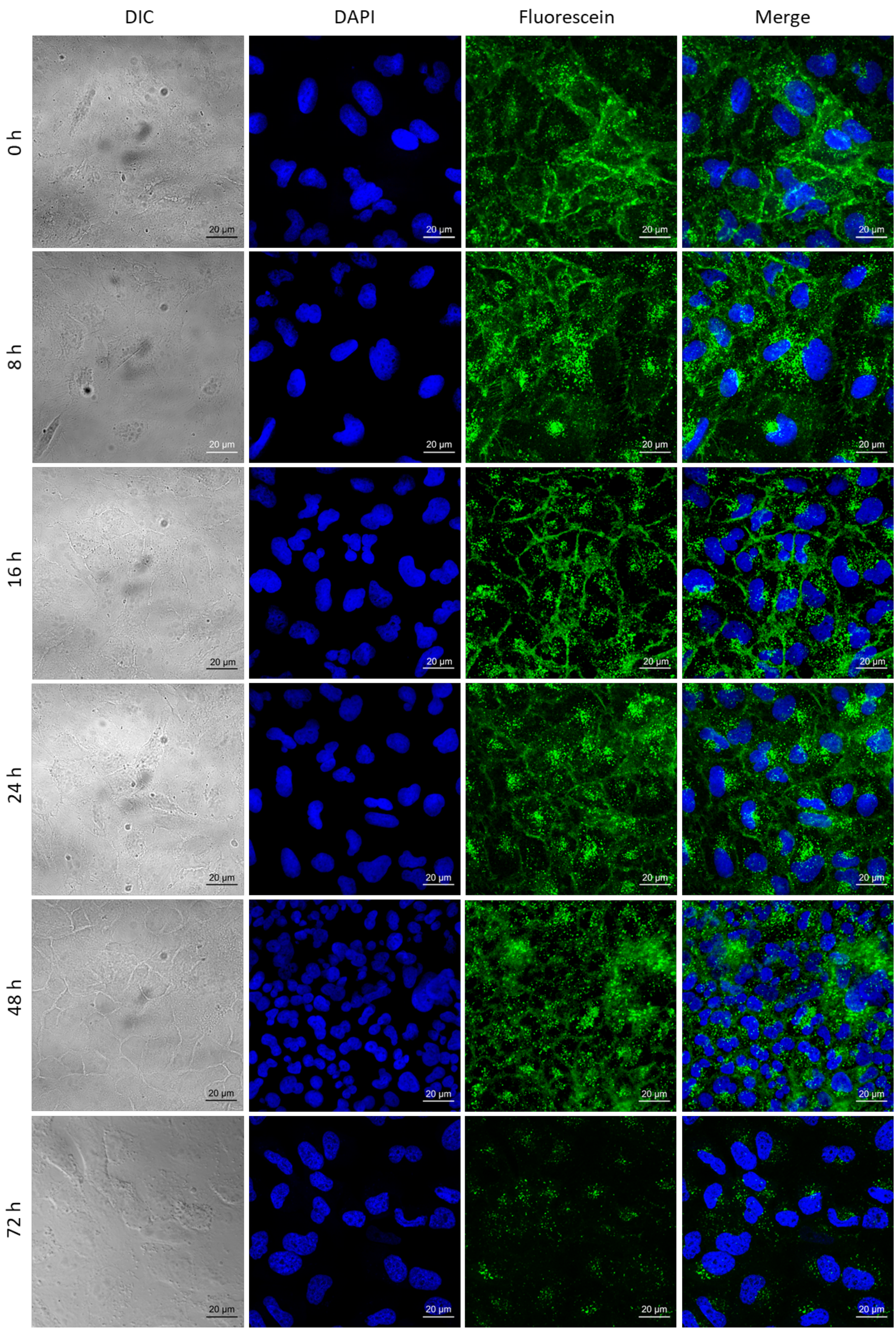

Figure S38. *Confocal imaging of control (fixed) A549 cells treated with Ac_4ManNAz (40 µM, 96 h) and DBCOpHEA$_{75}$Fl polymers (2.5 h, 10 mg.mL^{-1}) over time. Scale bar = 20 µm.*

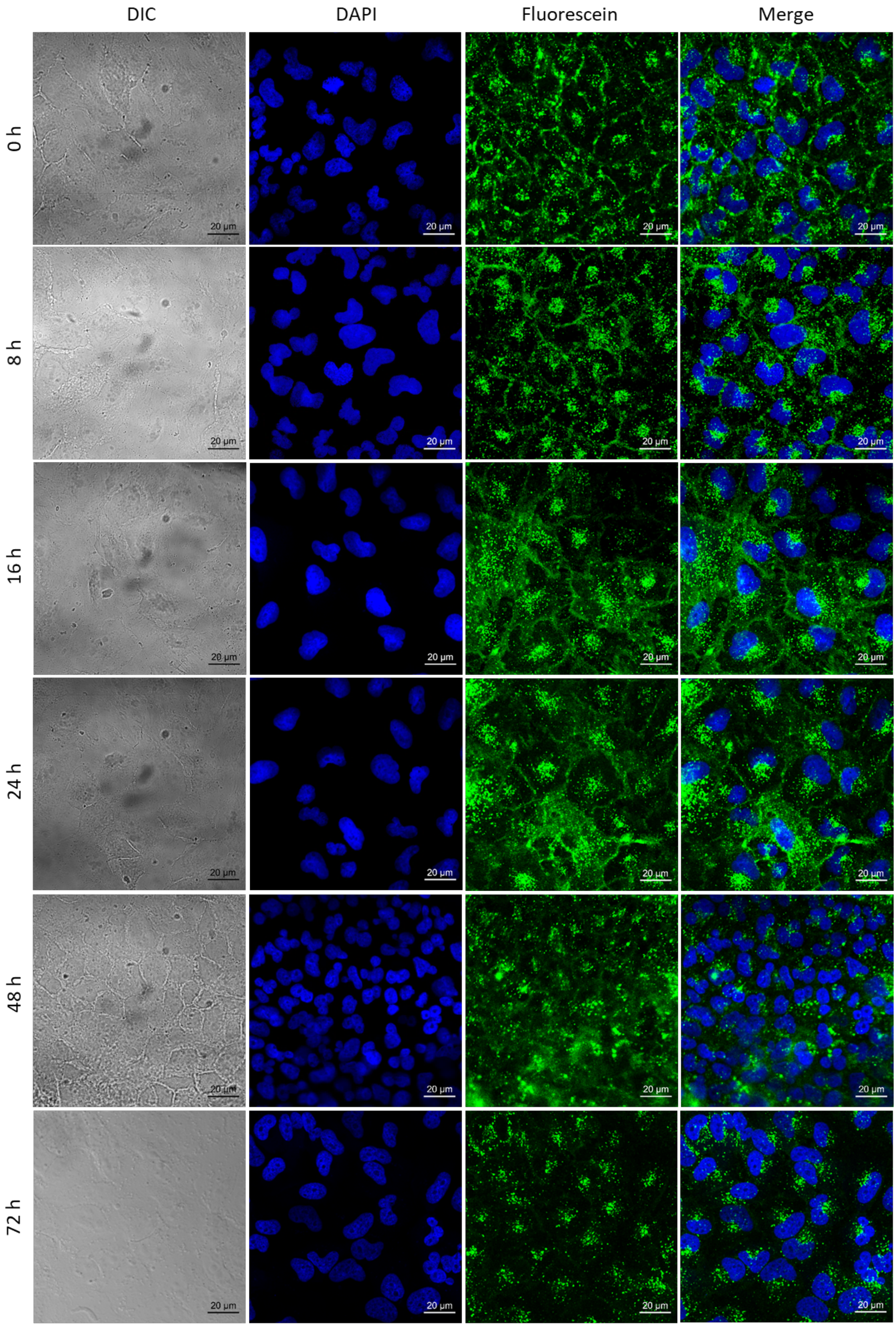

Figure S39. *Confocal imaging of control (fixed) A549 cells treated with* Ac_4*ManNAz (40 µM, 96 h) and DBCOpHEA*$_{100}$*Fl polymers (2.5 h, 10 mg.mL*$^{-1}$*) over time. Scale bar = 20 µm.*

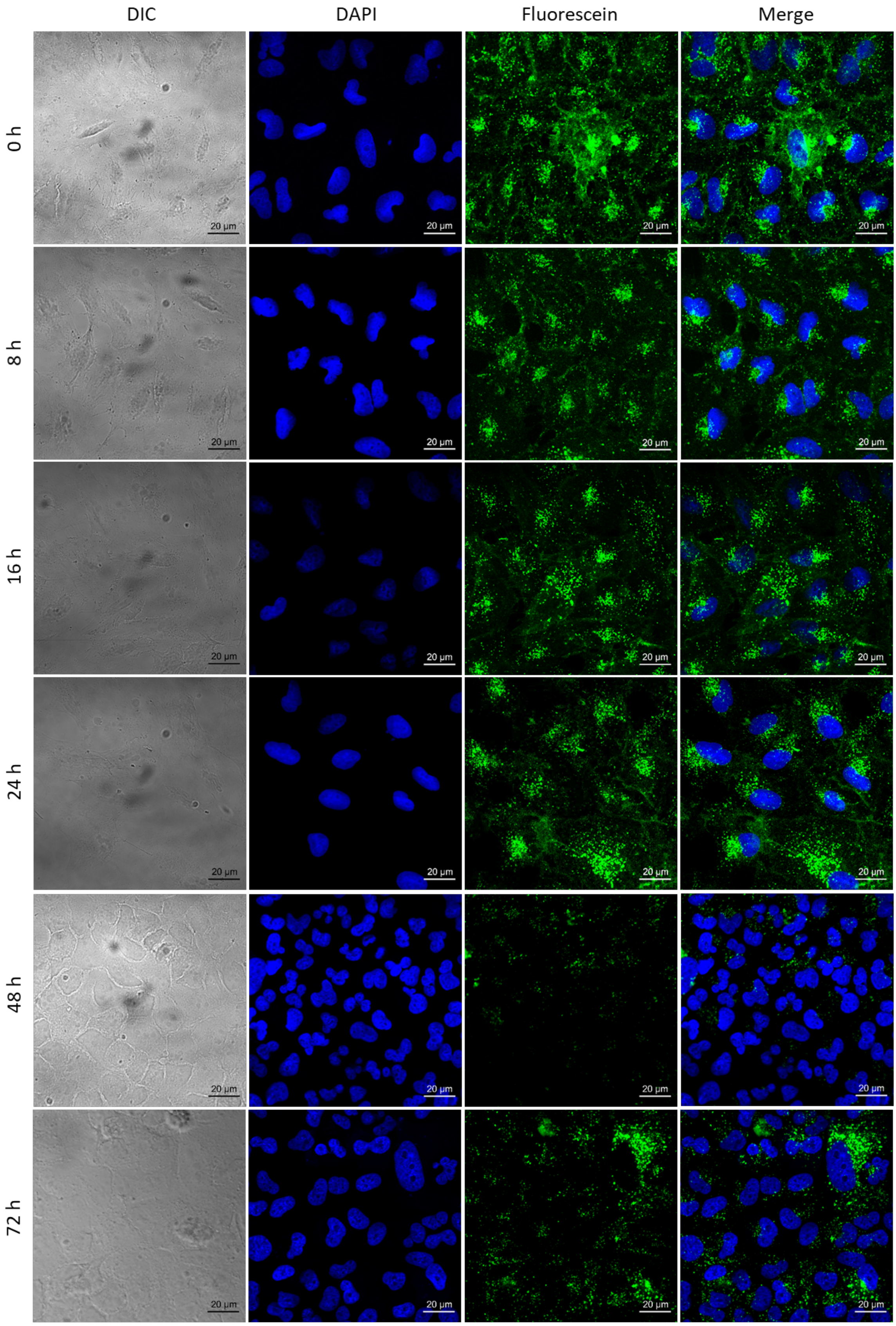

Figure S40. *Confocal imaging of control (fixed) A549 cells treated with $Ac_4ManNAz$ (40 µM, 96 h) and $DBCOpHEA_{125}Fl$ polymers (2.5 h, 10 mg.mL^{-1}) over time. Scale bar = 20 µm.*

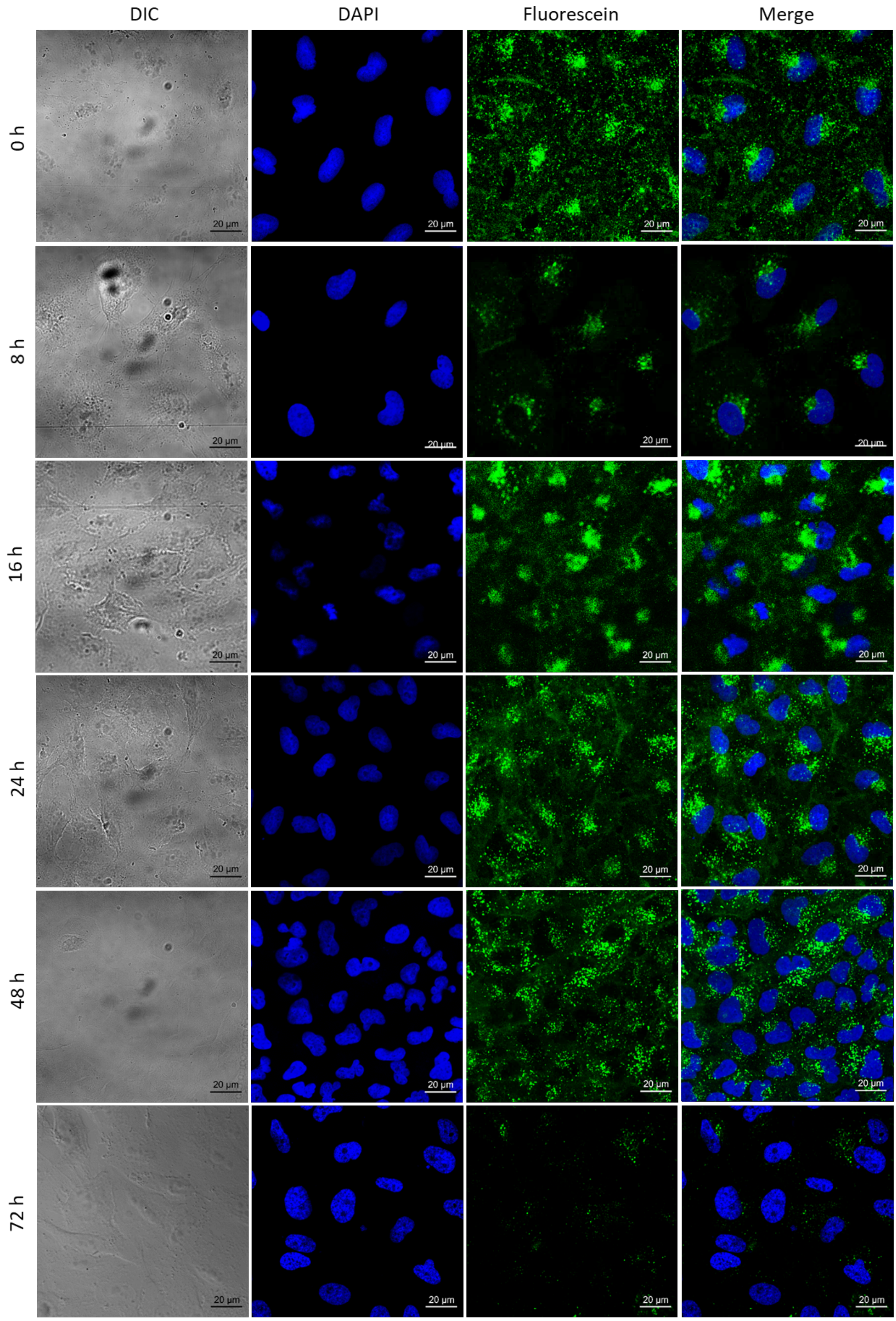

Figure S41. *Confocal imaging of control (fixed) A549 cells treated with* $Ac_4ManNAz$ *(40 µM, 96 h) and* $DBCOpHEA_{150}Fl$ *polymers (2.5 h, 10 mg.mL*$^{-1}$*) over time. Scale bar = 20 µm.*

2.5 Removal of non-specific bound pHEA$_n$DBCOFl over time

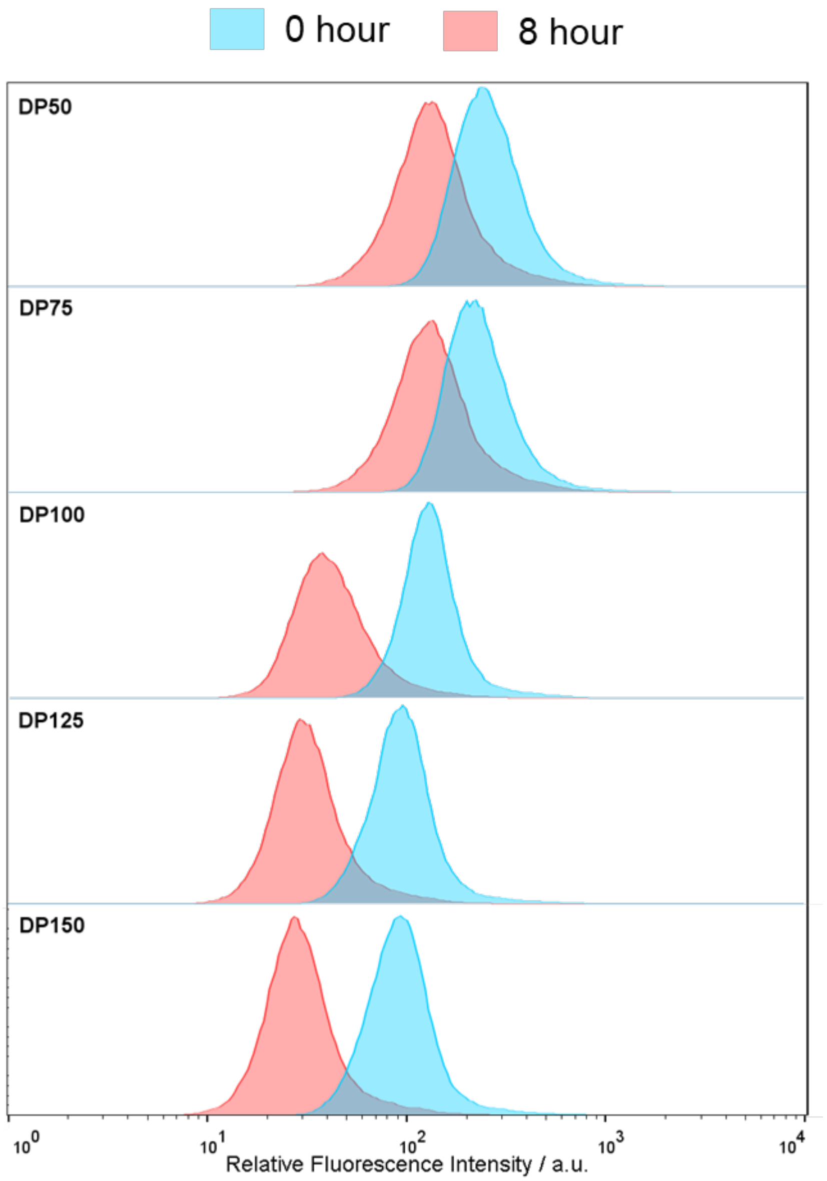

Figure S42. *Flow cytometry of (live) A549 cells untreated with $Ac_4ManNAz$ but incubated with polymers of different chain lengths (5 mg.mL^{-1}, 2.5 h) to investigate the loss of non-specifically bound polymer over time.*

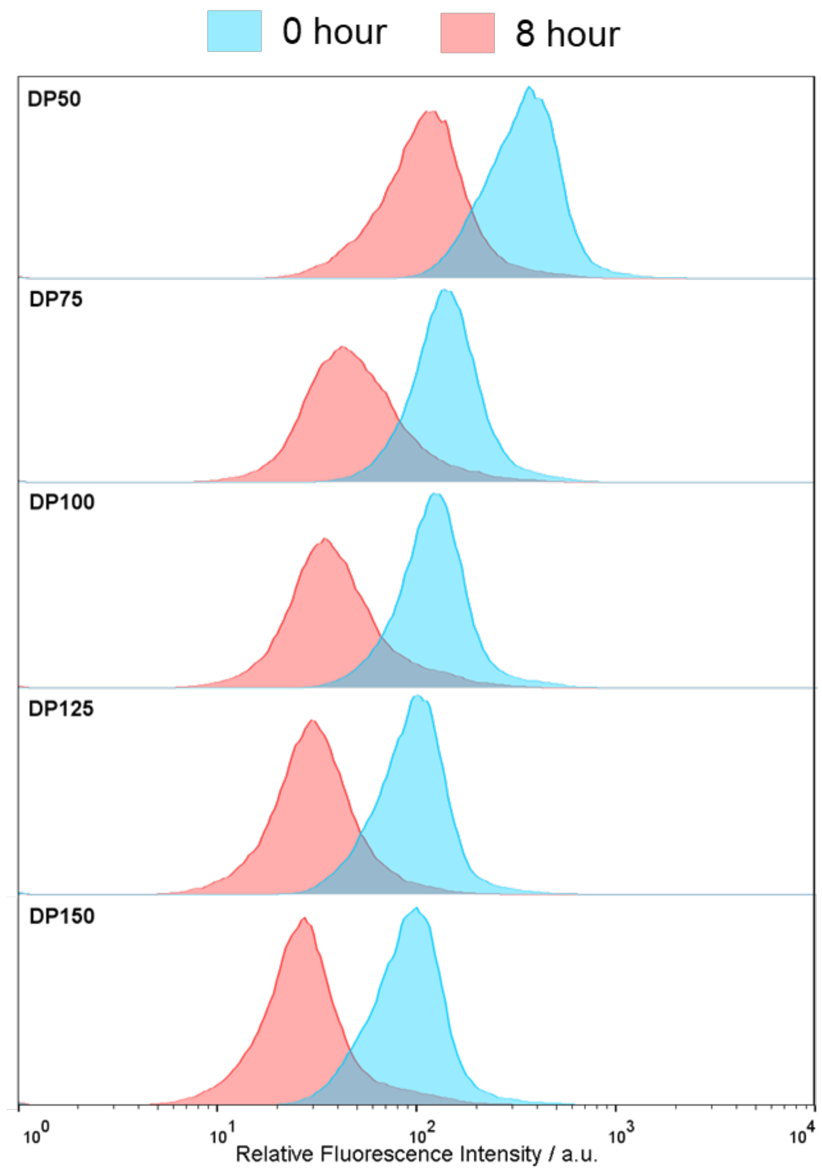

Figure S43. *Flow cytometry of (live) A549 cells untreated with Ac4ManNAz but incubated with polymers of different chain lengths (10 mg.mL^{-1}, 2.5 h) to investigate the loss of non-specifically bound polymer over time.*

Table S12. *Average fluorescence intensity of (live) A549 cells untreated with Ac4ManNAz but incubated with polymers of different chain lengths (2.5 h) to investigate the loss of non-specifically bound polymer over time using flow cytometry (n = 3).*

	Polymer concentration / $mg.mL^{-1}$			
	5		10	
Polymer	0 h	8 h	0 h	8 h
DBCO-$pHEA_{50}$	232 ± 4	110 ± 33	304 ± 50	111 ± 4
DBCO-$pHEA_{75}$	151 ± 1	68.8 ± 1.9	193 ± 33	53.7 ± 1.0
DBCO-$pHEA_{100}$	130 ± 4	41.9 ± 0.5	145 ± 25	32.6 ± 3.5
DBCO-$pHEA_{125}$	93 .2 ± 3.5	32.0 ± 0.7	111 ± 27	27.7 ± 3.0
DBCO-$pHEA_{150}$	90.3 ± 2.5	30.1 ± 0.4	107 ± 27	22.4 ± 2.5

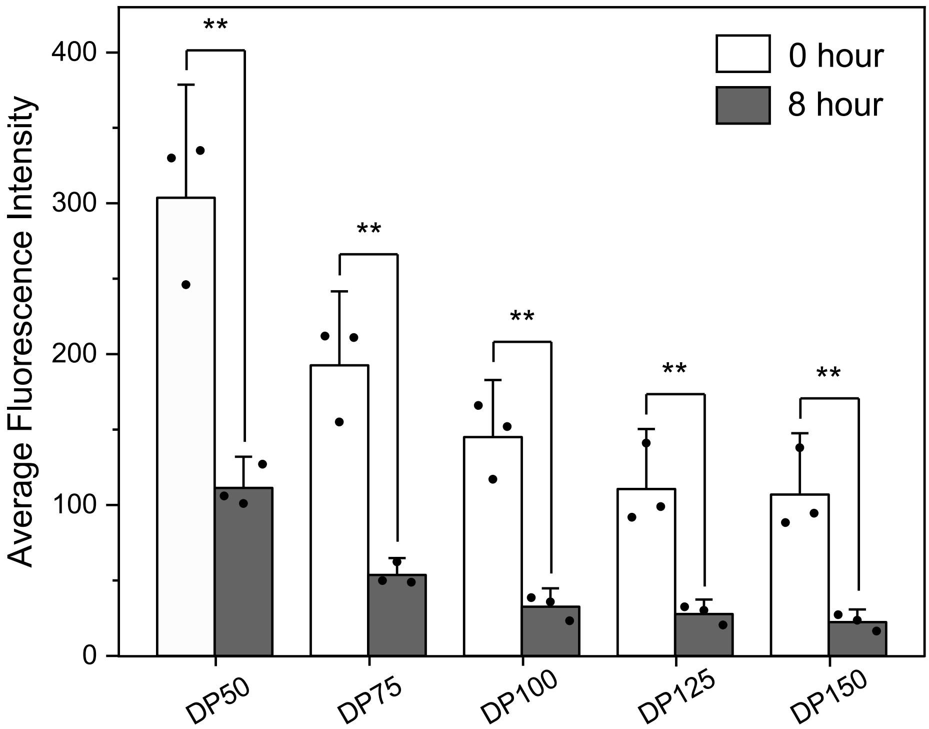

Figure S44. *Average fluorescence intensity of (live) A549 cells untreated with Ac4ManNAz but incubated with polymers of different chain lengths (5 $mg.mL^{-1}$, 2.5 h) to investigate the loss of non-specifically bound polymer over time using flow cytometry (n = 3).*

2.6 Neuraminidase Cleavage of Azido Sialic Acids

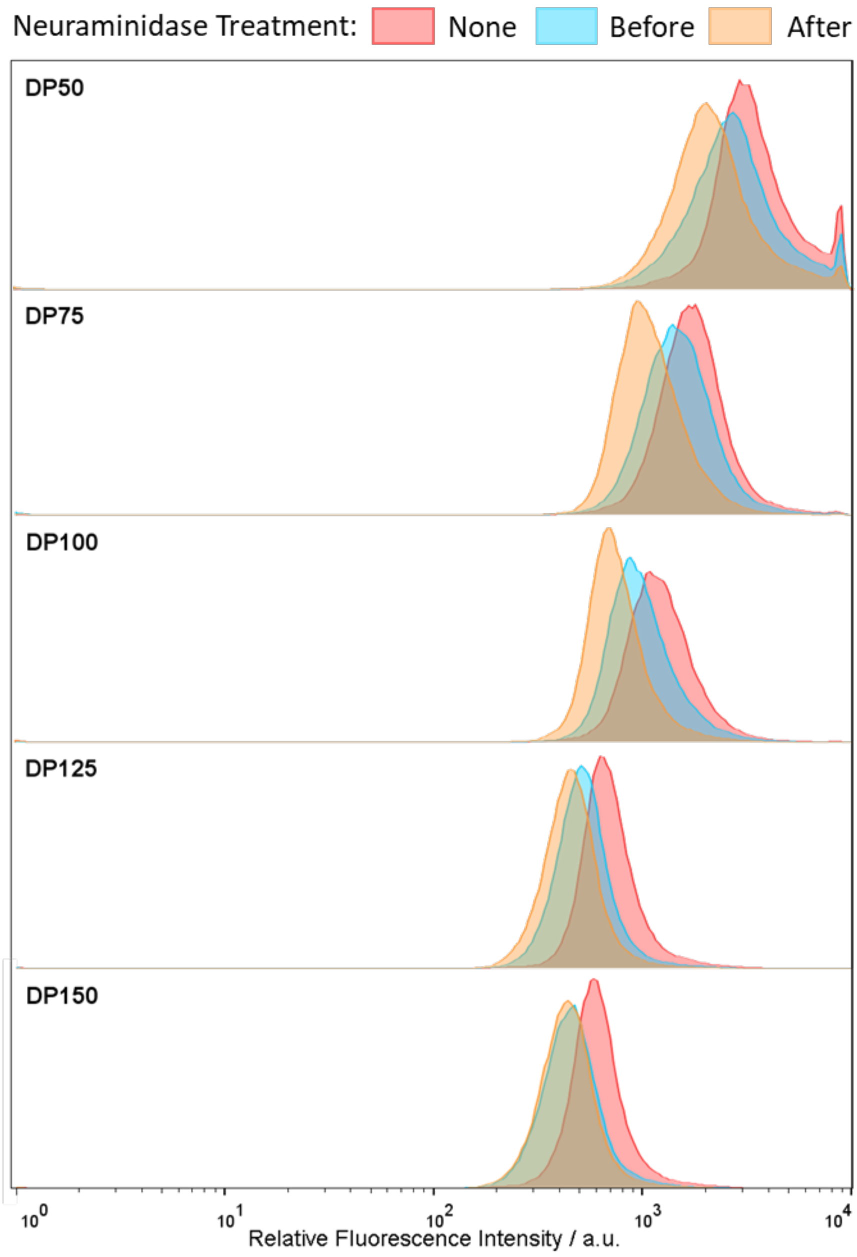

Figure S45. *Flow cytometry results of (live) A549 cells treated with $Ac_4ManNAz$ (40 µM, 96 h) and neuraminidase (25 u.mL^{-1}, 1.5 h) either before or after treatment with DBCO-pHEA$_n$-Fl (10 mg.mL^{-1}, 2.5 h) of different chain lengths.*

Table S13. *Average fluorescence intensity measurements obtained from flow cytometry of (live) A549 cells treated with $Ac_4ManNAz$ (40 µM, 96 h) and neuraminidase (25 $u.mL^{-1}$, 1.5 h) either before or after treatment with DBCO-$pHEA_n$-Fl (10 $mg.mL^{-1}$, 2.5 h) of different chain lengths (n = 4).*

Polymer	Neuraminidase Treatment		
	None	After	Before
DBCO-$pHEA_{50}$	3060 ± 98	2520 ± 252	2032 ± 74
DBCO-$pHEA_{75}$	1628 ± 47	1416 ± 41	1037 ± 16
DBCO-$pHEA_{100}$	1106 ± 20	950 ± 21	720 ± 11
DBCO-$pHEA_{125}$	659 ± 20	520 ± 20	452 ± 9
DBCO-$pHEA_{150}$	604 ± 37	461 ± 16	431 ± 10

Table S14. *Normalised Average fluorescence intensity measurements obtained from flow cytometry of (live) A549 cells treated with $Ac_4ManNAz$ (40 µM, 96 h) and neuraminidase (25 $u.mL^{-1}$, 1.5 h) either before or after treatment with DBCO-$pHEA_n$-Fl (10 $mg.mL^{-1}$, 2.5 h) of different chain lengths (n = 4).*

Polymer	Neuraminidase Treatment		
	None	After	Before
DBCO-$pHEA_{50}$	100 ± 4.5	82.4 ± 8.7	66.4 ± 3.2
DBCO-$pHEA_{75}$	100 ± 4.1	87.0 ± 3.6	63.7 ± 2.1
DBCO-$pHEA_{100}$	100 ± 2.6	85.9 ± 2.5	65.1 ± 1.6
DBCO-$pHEA_{125}$	100 ± 4.2	78.9 ± 3.8	68.6 ± 2.5
DBCO-$pHEA_{150}$	100 ± 8.7	76.4 ± 5.4	71.4 ± 4.7

3.0 References

(1) Richards, S. J.; Otten, L.; Gibson, M. I. Glycosylated Gold Nanoparticle Libraries for Label-Free Multiplexed Lectin Biosensing. *J. Mater. Chem. B* **2016**, *4* (18), 3046–3053.

(2) Richards, S. J.; Gibson, M. I. Optimization of the Polymer Coating for Glycosylated Gold Nanoparticle Biosensors to Ensure Stability and Rapid Optical Readouts. *ACS Macro Lett.* **2014**, *3* (10), 1004–1008.

(3) Tomás, R. M. F.; Martyn, B.; Bailey, T. L.; Gibson, M. I. Engineering Cell Surfaces by Covalent Grafting of Synthetic Polymers to Metabolically-Labeled Glycans. *ACS Macro Lett.* **2018**, *7* (11), 1289–1294.
